# Leveraging Administrative Health Databases to Address Health Challenges in Farming Populations: Scoping Review and Bibliometric Analysis (1975-2024)

**DOI:** 10.2196/62939

**Published:** 2025-01-09

**Authors:** Pascal Petit, Nicolas Vuillerme

**Affiliations:** 1 Laboratoire AGEIS Université Grenoble Alpes La Tronche Cedex France; 2 Institut Universitaire de France Paris France

**Keywords:** farming population, digital public health, digital epidemiology, administrative health database, farming exposome, review, bibliometric analysis, data reuse

## Abstract

**Background:**

Although agricultural health has gained importance, to date, much of the existing research relies on traditional epidemiological approaches that often face limitations related to sample size, geographic scope, temporal coverage, and the range of health events examined. To address these challenges, a complementary approach involves leveraging and reusing data beyond its original purpose. Administrative health databases (AHDs) are increasingly reused in population-based research and digital public health, especially for populations such as farmers, who face distinct environmental risks.

**Objective:**

We aimed to explore the reuse of AHDs in addressing health issues within farming populations by summarizing the current landscape of AHD-based research and identifying key areas of interest, research gaps, and unmet needs.

**Methods:**

We conducted a scoping review and bibliometric analysis using PubMed and Web of Science. Building upon previous reviews of AHD-based public health research, we conducted a comprehensive literature search using 72 terms related to the farming population and AHDs. To identify research hot spots, directions, and gaps, we used keyword frequency, co-occurrence, and thematic mapping. We also explored the bibliometric profile of the farming exposome by mapping keyword co-occurrences between environmental factors and health outcomes.

**Results:**

Between 1975 and April 2024, 296 publications across 118 journals, predominantly from high-income countries, were identified. Nearly one-third of these publications were associated with well-established cohorts, such as Agriculture and Cancer and Agricultural Health Study. The most frequently used AHDs included disease registers (158/296, 53.4%), electronic health records (124/296, 41.9%), insurance claims (106/296, 35.8%), population registers (95/296, 32.1%), and hospital discharge databases (41/296, 13.9%). Fifty (16.9%) of 296 studies involved >1 million participants. Although a broad range of exposure proxies were used, most studies (254/296, 85.8%) relied on broad proxies, which failed to capture the specifics of farming tasks. Research on the farming exposome remains underexplored, with a predominant focus on the specific external exposome, particularly pesticide exposure. A limited range of health events have been examined, primarily cancer, mortality, and injuries.

**Conclusions:**

The increasing use of AHDs holds major potential to advance public health research within farming populations. However, substantial research gaps persist, particularly in low-income regions and among underrepresented farming subgroups, such as women, children, and contingent workers. Emerging issues, including exposure to per- and polyfluoroalkyl substances, biological agents, microbiome, microplastics, and climate change, warrant further research. Major gaps also persist in understanding various health conditions, including cardiovascular, reproductive, ocular, sleep-related, age-related, and autoimmune diseases. Addressing these overlooked areas is essential for comprehending the health risks faced by farming communities and guiding public health policies. Within this context, promoting AHD-based research, in conjunction with other digital data sources (eg, mobile health, social health data, and wearables) and artificial intelligence approaches, represents a promising avenue for future exploration.

## Introduction

### Background

Public health research seeks to identify and understand the factors that influence population health to effectively prevent diseases and promote health and well-being for all [1,2]. A broad range of environmental determinants can impact health across the life span. One of the core areas of public health research, known as the exposome, investigates how cumulative environmental influences contribute to disease etiology and pathogenesis [3-18]. The exposome, which complements genomic research, refers to the comprehensive examination of all environmental exposures experienced throughout an individual’s lifetime, including physical, chemical, biological, psychosocial, and behavioral factors, from conception to death [3-18]. The exposome classically includes 3 overlapping domains: the general external exposome (eg, climate and built environment); the specific external exposome (eg, chemical exposure, lifestyle, and occupations); and the internal exposome (eg, aging, oxidative stress, metabolism, and gut microbiome) [8,14,16,17,19,20]. Understanding the exposome is crucial for enabling both population-wide and precision prevention [3,21-23]. However, fully describing the exposome is challenging due to the vast diversity and the temporal and spatial variability of environmental factors [3]. Public health research in this area requires data on both risk factors and adverse health outcomes to progress effectively [3,14,24,25].

The volume of data collected has grown exponentially as the world becomes increasingly reliant on technology and digitization [26,27]. Data are omnipresent in our everyday lives, leading science toward data-driven research [27,28], in particular in the health field. The digital transformation in health care has enabled unprecedented data availability, collection, storage, and analysis capabilities, leading to a paradigm shift in health care systems, with entire care pathways becoming digitized [29,30]. Health-related data now represent approximately 6% of all digital data globally, a figure that continues to rise [31]. This explosion of data has transformed research, providing new opportunities, especially in public health, to enhance disease understanding and evaluate intervention effectiveness [27,28,32-35]. The integration of digital technologies and digital data in public health has led to the emergence of “digital public health,” an evolving field focused on using digital data to achieve public health goals [33,36-41]. Public health research is moving from isolated data systems to more integrated, accessible, and reusable data resources [42]. Reusing data allows researchers to explore various health determinants, including environmental, occupational, behavioral, and organizational factors, fostering a holistic approach to disease prevention and health promotion strategies [14].

Within the digital public health framework, 2 main types of data are being used, namely primary and secondary data. Primary data are tailor-made, designed for a specific purpose, and often used once or repeatedly for the same goal [43-45]. Primary data are the cornerstone of traditional public health policy and decision-making. These data are derived from several types of studies [46-50], in particular observational cohorts (eg, the Framingham Cohort study [51,52]) [46-50,53,54], case-control studies [46-50], cross-sectional surveys (eg, the China Health and Retirement Longitudinal Study [55,56]) [46-50,53], and experimental studies [46-50]. Primary data have many advantages [46-50]. They are rich, of high quality, and are designed to answer specific research questions for public health and epidemiological purposes. Primary data are usually available at the individual level and are derived from studies that control for certain biases. By contrast, they are cumbersome, time-consuming, and costly to set up and maintain [53,54,57]. The representativeness of primary data is also limited in size, geographic scope, and temporal coverage and can erode with time [46-50,53]. Primary data are not free from bias, such as selection, healthy worker, recall, or prevarication biases [53,58].

Unlike traditional public health, digital public health does not rely solely on primary data but takes advantage of the myriad of existing digital data that have not been generated originally for research purposes (ie, secondary data) to overcome some limitations intrinsic to primary data and complement them [28,43,44,53,59-68]. Indeed, some data can have an additional impact when used beyond the context for which they were originally created [68,69]. Secondary data are collected for purposes other than public health or epidemiology and include contextual data (eg, air quality and climate data) [14,24,26,29,70-74], person-generated data (eg, social media, crowdsourcing, and mobile health) [2,24,26,31,43,61,62,73,75-86], synthetic data (eg, digital twin) [87-91], and administrative health databases (AHDs) [26,64,68,81,92-102].

AHD is a broad term encompassing a wide range of routinely collected data on individuals’ health and sociodemographic information collected for registration, billing, record keeping, and other administrative purposes [26,64,81,93,95,98,100-102]. For this review, based on previous works [93,95,96,103-105], AHDs included population registers, claims databases, disease registers, electronic health or medical records, and hospital discharge databases that were collected at a local, regional, national, or international level (Table 1) [26,61,62,93,95,96,100,103-110].

**Table 1:** Definition and characteristics of administrative health databases included in this review.

	Population register	Claims database	Disease register	Electronic health or medical record	Hospital discharge database
Definition	Digital sociodemographic information on the residents of a country	Routinely collected digital information on individual data regarding reimbursement, records of health services, medical procedures, prescriptions, and medical diagnoses	A continuous and exhaustive digital collection of individual data regarding 1 or more health events in a geographically defined population	Systematized digital record of a patient’s medical information collected in real time	Digital records of service use with information about patients, their care, and their stay in the hospital
Source	Local or national authorities	Insurance programs or schemes and health care providers	Health care institutions (eg, hospitals)	Hospitals, physicians, health care centers, and institutions	Hospitals
Population	All individuals residing in a country	All individuals covered by an insurance program or scheme	All individuals diagnosed with a specific health event in a population on a geographically defined scale	All patients using the health care system	All patients from a hospital
Purpose or finality	For the administrative purposes of government: to provide reliable information	To store financial and administrative information for medical insurers’ and providers’ use	For clinical and research purposes: to collect information about people diagnosed with a specific health event	For clinical and billing purposes: to document patients’ clinical condition	For billing or accounting purposes
Health event	None	Health events covered by insurance or a health care provider	Specific health events (eg, cancer)	Health events requiring care that are reported in medical records	Health events from hospital admission

AHDs offer many advantages for research. Such data are collected as part of routine administrative processes, reducing additional costs for researchers. Therefore, AHDs offer relatively inexpensive access to a large number of individuals who can be tracked with time for several years, guaranteeing the representativeness of the populations studied [26,54,78,93,95,104,105,111-114]. Data recorded within AHDs are structured, coded in a standardized way, and less affected by participation and recall biases [54,58,95,106,113,115]. AHDs enable the study of rare events and populations underrepresented in studies using only primary data [95,111-113]. AHDs have limitations inherent to their nature, such as the absence of some confounding factors, the limited granularity of certain information, the data complexity, and confidentiality issues [73,78,93,95,115-125].

### Rationale

AHDs are increasingly used in population-based health research due to their complementarity with traditional sources of public health and epidemiological data (ie, primary data) [42,59,64,87,93,95,96,126-128]. The reuse of AHDs, referring to their application beyond their original or intended purpose, holds major potential to advance public health and epidemiological research, offering insights that can guide public health decision-making [42,59,64,87,93,95,96,105,126-131]. Although several reviews have previously explored the general use of AHDs in research [42,95,107,129,132-135], others have focused on their application within specific countries [96,136], examined individual AHDs [108,137], or investigated their role in studying specific diseases and adverse health outcomes [44,93,104,138-142]. However, to the best of our knowledge, no study has synthesized how AHDs are reused for epidemiological and public health research within a specific population group.

To address this gap, we conducted a comprehensive scoping review and bibliometric analysis aimed at identifying how AHDs are used to address health issues in a specific population. We selected farming populations as an illustrative example because they present unique health and disease patterns [143-147]. Globally, approximately 27% of the workforce is engaged in occupational farming, and this group is exposed to numerous risk factors (ie, exposomes), including pesticides, biological agents, and limited access to health care [148]. These exposures put them at heightened risk for a wide range of adverse health outcomes [143,145,147,149]. Although agricultural safety and health have become a major public health issue in recent decades, most research on the health of farming populations has relied on traditional epidemiological and community-based studies, which often face limitations in terms of sample size, geographic scope, temporal coverage, and the range of health events examined [145,150,151].

In this context, AHDs offer valuable opportunities to enhance public health and epidemiological research in farming populations by providing broader insights, identifying at-risk subgroups, and informing health services and policy development [152]. The primary objectives of this scoping review were two-fold: (1) to summarize the current state of AHD-based research in farming populations by examining which types of AHDs are used and why, whether AHDs are integrated with other data sources, which farming populations have been studied, and what exposures and health outcomes have been explored and (2) to identify key areas of interest and potential research gaps and unmet needs in this field.

## Methods

### Overview

This scoping review was conducted and reported according to the PRISMA-ScR (Preferred Reporting Items for Systematic Reviews and Meta-Analyses extension for Scoping Reviews) and evidence maps guidelines (Table S1 in Multimedia Appendix 1) [153] following a single screening approach. The protocol of this study was not registered. A 7-step procedure was used: research question formulation, identifying relevant publications, title review, abstract review, full-text review, data extraction, and data analysis.

To formulate our research question, we followed the Joanna Briggs Institute guidelines, using the population, concept, and context criteria framework [154]. Our population included all individuals engaged in farming and all individuals exposed to farming-related exposures. The concepts included all possible public health and epidemiological research works that involved the study of a health outcome of interest. The context was the use, in any setting, of at least one of the AHDs, as defined in Table 1.

### Search Strategy and Selection Criteria

To develop and validate the search strategy, previous reviews that examined the reuse of AHDs for population-based research were identified and refined [93,96,103,104]. Our initial search revealed that electronic health records (EHRs) are often interchangeably referred to as electronic medical records (EMRs). A distinction between EHR and EMR is sometimes made, with EMR describing patients’ care from only 1 practice (eg, specific encounters in hospitals), which is contrary to EHR [105]. In that case, EMR serves as a data source for EHR. This distinction was not considered in this paper. In addition, to ensure comprehensiveness, the search terms were broadened by searching for their synonyms. For example, search terms such as “electronic health record,” “digital health record,” “electronic medical record,” “EHR,” or “EMR” were used as synonyms for electronic health or medical records. A total of 72 terms pertaining to 2 categories (farming and AHDs) were used (Textbox 1). The search terms were reflective of our research topic and question.

**Search terms.**

**Farming**

- husbandry* OR agriculture* OR farming OR farm* OR agricultural* OR farmworker*

**Administrative health databases (combined using AND)**

- “health record” OR “health records” OR “digital record” OR “digital records” OR “health administrative register” OR “health administrative registry” OR “health register” OR “health registry” OR “medical register” OR “medical registry” OR “electronic health record” OR “electronic health records” OR “EHR” OR “EMR” OR “electronic medical record” OR “electronic medical records” OR “digital medical record” OR “digital medical records” OR “digital health record” OR “digital health records” OR “health administrative data” OR “health administrative database” OR “ health administrative dataset” OR “ health administrative datasets” OR “health administrative databases” OR “administrative health data” OR “administrative health database” OR “administrative health dataset” OR “administrative health datasets” OR “administrative health databases” OR “insurance data” OR “insurance database” OR “insurance databases” OR “insurance dataset” OR “insurance claim” OR “insurance claims” OR “cancer registry” OR “cancer register” OR “health insurance” OR “health surveillance program” OR “health surveillance programs” OR “Mutualite Sociale Agricole” OR “MSA” OR “health insurance system” OR “record-linkage” OR “population register” OR “population registry” OR “insurance scheme” OR “social security scheme” OR “hospital discharge” OR “administrative claim” OR “administrative claims” OR “medical claims” OR “medical claim” OR “electronic claim” OR “electronic claims” OR “mortality register” OR “mortality registry” OR “mortality record” OR “mortality records” OR “disease register” OR “disease registry” OR “illness register” OR “illness registry” OR “disorder register” OR “disorder registry”

To develop the eligibility criteria, an initial search of the literature was conducted on PubMed, with a review of the first 100 articles that used AHDs for public health and epidemiological research. In our pilot run, disease and morbidity registers were initially not considered as AHD because they were created for clinical and research purposes [47-50,53,76,155,156]. However, because disease registers contain some information derived from medical records, we decided to consider them as AHD for this review. The eligibility criteria are presented in Textbox 2. The search was restricted to original peer-reviewed records (all types were included) written in English or French but not constrained by the year of publication [93,106,157]. Publications that examined partly farming populations, with, for instance, studies reporting health risks for various sectors of activity, were included.

**Eligibility criteria for selection of publications.**

**Inclusion criteria for articles**

- Data had to originate at least partly from the administrative health database (AHD)
- The study had to pertain at least partly to the farming population
- The study had to relate to public health or epidemiological research
- Original peer-reviewed publications
- Publications in English or French

**Exclusion criteria for articles**

- Publications not describing the use of an AHD
- Animal or in vitro studies
- Publications not in English or French

The final literature search was conducted on both PubMed and Web of Science Core Collection databases. Regarding the Web of Science Core Collection, a topic search was performed. To reduce the bias induced by daily database changes, all data collection (literature retrieval and data download) was conducted and completed on the same day, that is, April 15, 2024. Titles, abstracts, and full-text publications were screened based on pre-established inclusion and exclusion criteria. The inclusion criteria for each phase of the literature search are provided in Table S1 in Multimedia Appendix 1. When abstracts did not contain enough information about correspondence to inclusion or exclusion criteria, the article was considered for full-text review. Reference lists of included publications were not searched, although they might have also yielded new relevant studies.

### Data Collection and Processing

A total of 29 metadata were extracted from each publication included in the scoping review (Table 2).

The data underwent rigorous manual validation, cleaning, and harmonization following a structured 5-step process. First, duplicate items (eg, keywords and institutions) were removed. Second, leading and trailing white spaces were eliminated. Third, items were standardized by converting text to lower case, with only the first letter capitalized. In the fourth step, items were harmonized to either singular or plural forms consistently. Finally, synonyms or terms with similar meanings (eg, “illness” and “disease”) were unified under a single term. For instance, “Pesticide,” “Pesticide exposure,” and “Pesticide use” were standardized to “Pesticide,” while “Pulmonary disease copd,” “Copd,” and “Chronic obstructive pulmonary disease” were unified as “COPD.” For cancer-related keywords, the International Classification of Diseases, eleventh revision, was used to consolidate varied terms (eg, “lung cancer,” “lung cancer risk,” “lung and bronchus cancer,” “lung tumor,” “lung tumour,” “lung neoplasm,” and “basal cell carcinoma of the lung”) into standard categories (eg, lung cancer). Quality appraisals were not performed because they were beyond the aim of this review [106,157].

**Table 2:** List of metadata of interest to collect from the literature search.

Metadata	Fictional example
Publication year	2024
Publication type	Article
Study name	Project X
Goal of the study	To study the association between farming and health outcome
Study type	Ecological study
Is the study nationwide?	Yes
Digital data used	Insurance claims
Goal of the digital data used	To identify farmers
Is active data used?	Yes
Active data used	Clinical examination
Farming exposure considered	Farming activity and pesticide compounds
Farming activities studied, n	10
Pesticide compounds studied, n	29
Population	Adults
Sex	Female
Participants included, n	100 to 1000
Country	France
Oldest data used (year)	1991
Most recent data used (year)	2020
Data follow-up period (years)	4
Years between the most recent data used and publication year, n	7
Disease or health events	Parkinson disease
Authors’ names	Gauthier J
Authors’ keywords	Pesticide
Authors’ country	France
Authors’ institution	Université Grenoble Alpes
Journal	Environmental Health Perspectives
Funding body	MIAI@Grenoble Alpes^a^
Citations, n	14

^a^MIAI@Grenoble Alpes: Multidisciplinary Institute in Artificial Intelligence at the Université Grenoble Alpes.

### Data Analysis

To analyze the research directions (ie, hot spots and gaps) on the use of AHDs for public health and epidemiological research in farming populations, a bibliometric approach was conducted [158-160]. This analysis examined the number of publications, countries of publications, most active journals, institutions, authors, funding bodies, subject areas, citations of publications, and keywords of publications. Seven bibliometric metrics were computed, including the h-, g-, m-, and Y-indices; dominance factor; annual growth rate (AGR); and fractionalized frequency (Table S3 in Multimedia Appendix 1). The h-index attempts to measure both the productivity and citation impact of the published body of work of an entity (eg, author, institution, and journal) [161,162]. It refers to the total number of publications by a particular entity with at least the same number of citations. The m-index is calculated by dividing the h-index by the number of years of an entity’s productive life (eg, researcher) [161]. The g-index of an entity corresponds to the largest number g such that the top g publications have at least ≥g^2^ citations together [162]. The Y-index refers to the sum of both the total number of first-authored publications and the total number of corresponding author publications [163]. The dominance factor refers, for a particular researcher, to the proportion of multiauthored publications as a specific author’s rank to the total number of multiauthored publications [164]. The fractionalized frequency intends to reflect an author’s contribution. The AGR refers to the variable’s change in percentage as a year-over-year statistic [165]. The most up-to-date journals’ impact factors and ranks were retrieved manually using the Journal Citation Report in April 2024.

Spearman correlations were calculated to examine the association between the number of publications and gross domestic product (GDP); population size [166]; and the total labor force, the number of researchers in research and development (per million people), fertilizer consumption (in both % of fertilizer production and kilograms per hectare of arable land), agricultural land (km^2^), agricultural land (% of land area), land under cereal production (hectares), permanent cropland (% of land area), cereal production (metric tons), crop production index, food production index, livestock production index, cereal yield (kilogram per hectare), female individual employment in agriculture (% of female employment), male individual employment in agriculture (% of male employment), employment in agriculture (% of total employment); and agriculture, forestry, and fishing, value added (% of GDP). These country characteristics were obtained from the World Bank. The most recent country characteristic (eg, GDP) was used when available.

Research directions, including hot spots and gaps, were investigated with keyword frequency, co-occurrence (counting of paired keywords), and thematic mapping analyses. Thematic mapping and keyword co-occurrence network are 2 complementary but distinct approaches that serve different purposes and offer different insights. In summary, thematic mapping focuses more on the strategic positioning of research themes within a field, while keyword co-occurrence networks emphasize the relationships and connections between specific keywords in the literature [158,167]. Both methods complement each other and are usually used to provide a more comprehensive understanding of research landscapes. The co-occurrence of 2 keywords was defined by the frequency with which they appear together in publications and was quantified using association strength (AS) or equivalent index, calculated as 
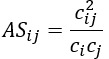
, where *c_ij_* is the number of publications in which keywords *i* and *j* co-occur, while *c_i_* and *c_j_* are the number of publications in which each keyword appears, respectively [158,167]. AS measures how close 2 keywords are to each other. An AS value of 1 indicates keywords always appear together, while 0 indicates they never co-occur. These keyword co-occurrences can be visualized using a co-occurrence network graph, where a vertex or node represents a keyword, the size of the node represents the keyword frequency, and the edge represents the association between 2 keywords [158,167]. On the basis of the keyword co-occurrence network graph, a community detection procedure can be used to identify groups of words highly associated with each other [158,167]. In other words, equivalent keywords based on AS can be grouped together to identify research themes [158,167]. A strategic diagram or thematic map is based on Callon centrality (x-axis) and Callon density (y-axis) [158,167]. Callon centrality measures the degree of interaction of a theme with other themes. It is defined as 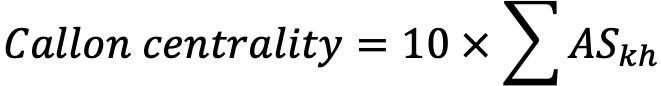, where *k* is a keyword belonging to a theme and *h* is a keyword belonging to another theme [158,167]. Callon centrality can be interpreted as an indicator of the importance of a particular topic within the broader research landscape. Callon density measures the internal strength of a theme. It is defined as 
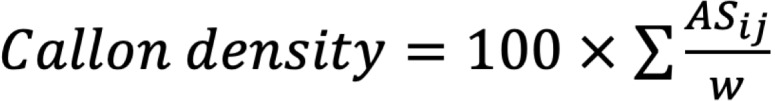
, where *i* and *j* are keywords belonging to the same theme and *w* is the total number of keywords in a theme [158,167]. Callon density serves as a metric for assessing the progression and maturation of that topic [158,167]. A strategic diagram is divided into 4 quadrants according to Callon centrality and density values, which correspond to 4 types of topics. Hot spots or hot topics are defined by both high density and high centrality values (upper-right quadrant), while basic topics are defined by high centrality but low density values (lower-right quadrant). Peripheral topics are defined by both low centrality and low density values (lower-left quadrant), while niche topics are defined by low centrality and high density values (upper-left quadrant) [158,167].

To focus on agricultural or farming exposome research, a bibliometric profile of the “farming exposome” was constructed, which restricts the exposome concept to environmental exposures specific to farming populations [152,168]. This bibliometric farming exposome picture examined co-occurrences between keywords related to potential risk factors and specific health events (eg, cancers and reproductive disorders).

The bibliometric analysis was conducted and reported according to the preliminary guideline for reporting bibliometric reviews of the biomedical literature (BIBLIO; Table S4 in Multimedia Appendix 1) [169]. All analyses were performed using R software (version 4.3.2; R Foundation for Statistical Computing) for Windows 10 (Microsoft Corporation). The bibliometric analysis was performed using the *bibliometrix* R package (version 4.1.4) [170].

## Results

### Overview

**Figure 1:**
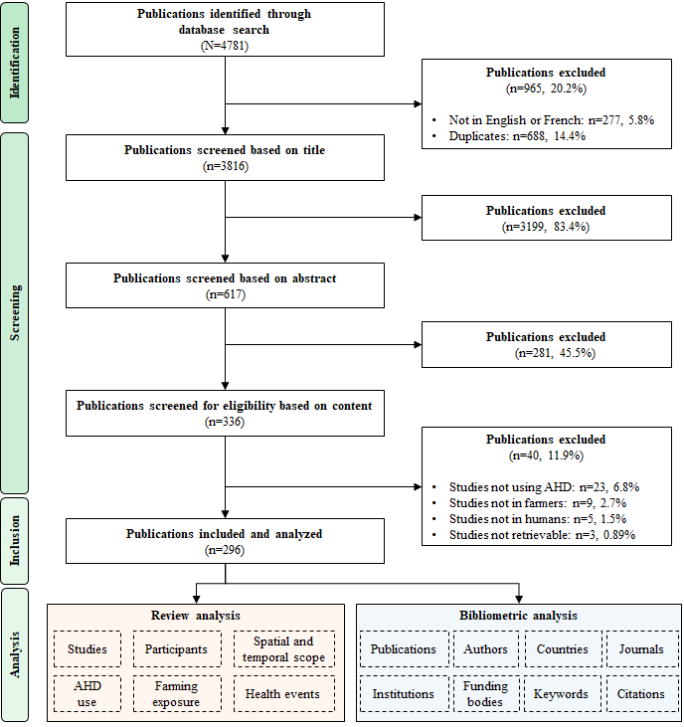
PRISMA-ScR (Preferred Reporting Items for Systematic Reviews and Meta-Analyses extension for Scoping Reviews) flowchart depicting the literature search and the evaluation process for finding relevant records. The search, conducted on April 15, 2024, in PubMed and Web of Science, had no date restrictions. AHD: administrative health database.

After excluding 4485 irrelevant records, 296 publications were analyzed (Figure 1). The majority were articles (293/296, 98.9%), with a small number of reviews (2/296, 0.7%) and editorial materials (1/296, 0.3%; Table 3). Only one-third of the publications (107/296, 36.1%) were open access (Table 3 and Figure S1 in Multimedia Appendix 1).

**Table 3:** Main characteristics of the included publications (N=296).

Description			Results
Timespan			1975 to 2024
**Publication type, n (%)**			
	Article	293 (99)	
	Review	2 (0.7)	
	Editorial material	1 (0.3)	
Open-access publications, n (%)			107 (36.1)
Document age (y), mean (SD)			14.2 (11.8)
Annual growth rate (%)			5.2
**Publication citations**			
	Total, n^a^	9379	
	Average citations per publication	31.7	
	Average citations per year per publications	2.02	
References, n			8814
**Journals**			
	Total, n	118	
	Average number of publications per journal	1.86	
	Average number of citations per journal	79.5	
**Authors**			
	Total, n	1225	
	Single-author publications, n	4	
	Author appearances, n	1882	
	Average number of coauthors per publication	6.36	
	Average number of publications per author	0.24	
	International coauthorships (%)	24.3	
	Author’s keywords, n	576	
**Author’s country**			
	Total, n	34	
	Average number of publications per country	2.86	
	Average number of citations per country	436.0	
**Author’s institution**			
	Total, n	338	
	Average number of publications per institution	3.11	
	Average number of citations per institution	101.3	
**Author’s funding body**			
	Total, n	181	
	Average number of publications per funding body	2.48	
	Average number of citations per funding body	77.7	

^a^Total, n indicates that the respective parameter has been cited n number of times, as in 296 publications have been cited 9379 times.

The average publication age was 14.2 (SD 11.8) years, ranging from the oldest in 1975 [171] to the most recent in April 2024 [152]. From 1975 onward, there has been a steady increase in publications using AHDs to address health issues in farming populations, with an AGR of 5.2%. Notably, almost one-third of these articles (91/296, 30.7%) were published in the last 5 years, highlighting the rising interest in AHD-based public health research in this population (Figure S2 in Multimedia Appendix 1). Collectively, the publications received 9379 citations, averaging 31.7 citations per publication. Figure S3 in Multimedia Appendix 1 presents the historical direct citation network. The body of work involved 1225 authors from 338 institutions, with 1882 author appearances and an average of 6 authors per paper (Table 3). Four (1.4%) out of the 296 publications were single-author publications. On average, each paper cited 30 references.

Studies were led by authors from 34 countries, predominantly high-income nations, with 24.3% (72/296) of studies involving multicountry collaborations (Figure S4 in Multimedia Appendix 1). US-based authors contributed the most publications (91/296, 30.7%), followed by authors based in France (71/296, 24%) and Finland (35/296, 11.8%). US authors also had the most citations (3495/9379, 37.2%), with France and Finland ranking second and third, respectively.

Of 296 publications, the 25 (8.4%) most cited ones, appearing in 17 different journals, received between 83 (83/9379, 0.9%) and 485 (485/9379, 5.2%) citations (Table S5 in Multimedia Appendix 1) [150,172-196]. Of these 25 publications, 10 (40%) were published before 2000, another 10 (40%) between 2000 and 2010, and 5 (20%) after 2010. Most of these studies focused on cancer risk (16/25, 64%), while others investigated neurodegenerative disorders (5/25, 20%); respiratory conditions (2/25, 8%); and multiple health outcomes, such as sleep disorders, mental health disorders, and musculoskeletal disorders (2/25, 8%).

Table S6 in Multimedia Appendix 1 provides details on the most productive countries, prolific authors, active journals, institutions, and funding bodies.

### Study Characteristics

Table 4 provides an overview of the included publications. Longitudinal study designs were the most common, including retrospective cohorts (129/296, 43.6%) and prospective cohorts (56/296, 18.9%). Case-control studies (62/296, 20.9%), cross-sectional studies (39/296, 13.2%), and ecological studies (17/296, 5.7%) were less common (Multimedia Appendix 2). A few studies (10/296, 3.4%) used multiple study designs [188,194,197-204].

**Figure 2:**
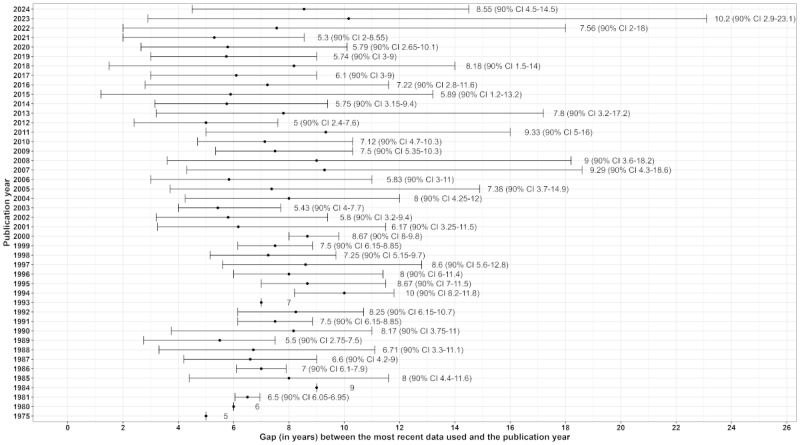
Number of years between the most recent data used and publication for all included articles (1975-2024). Points refer to the average number of years or gap between the most recent data used and publication (x-axis) for each publication year (y-axis). Error bars refer to the 90% CI of the number of years between the most recent data used and publication.

The median follow-up period was 9.5 (IQR 5-17) years. On average, there was a 7-year gap (90% CI 3-14) between the most recent data used and the year of publication, with considerable variation depending on the publication year (Figure 2). The oldest data were from 1801 [205], and the most recent data were from 2022 [206]. Notably, one-third of the data used (98/296, 33.1%) were from before 2000, while nearly three-quarters (214/296, 72.3%) were from before 2015 (Figure S2 in Multimedia Appendix 1). Of 296 studies, only 10 (3.4%) used data from the last 5 years (from 2020), while 80 (27%) used data from the last 10 years (from 2015).

**Figure 3:**
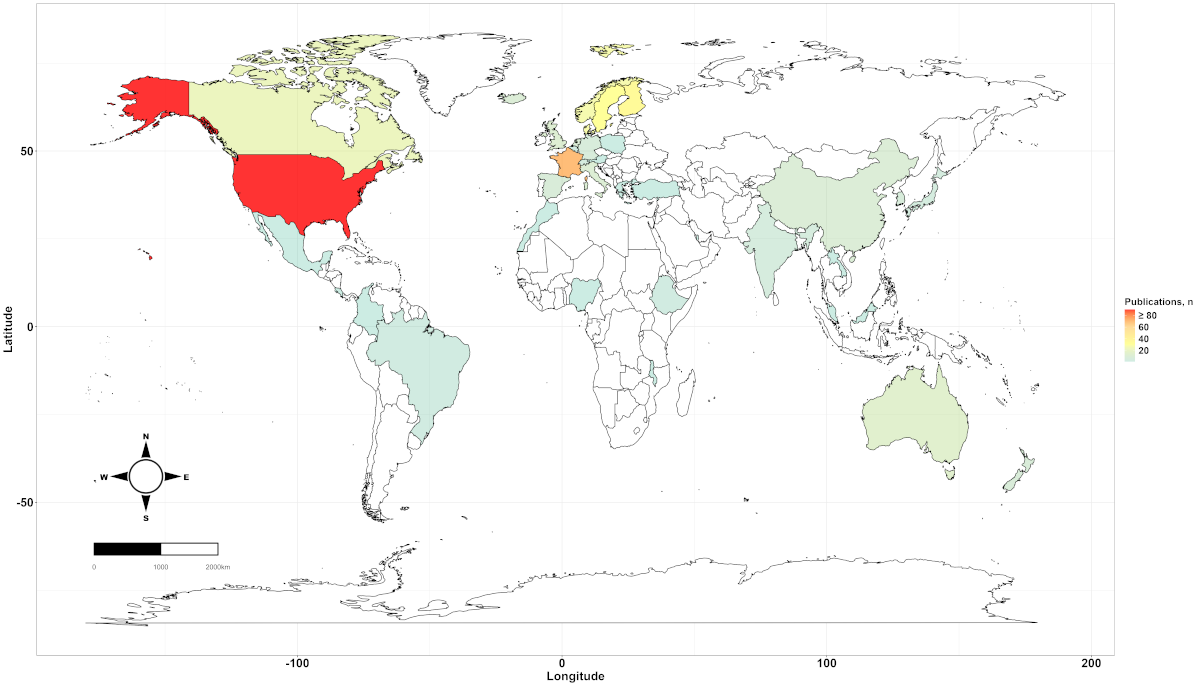
World map of the number of publications per country of the farming population studied between 1975 and 2024.

Studies were conducted in all continents, but most participants were from Europe (249/296, 84.1%), followed by North America (85/296, 28.7%), Asia (24/296, 8.1%), Oceania (17/296, 5.7%), Africa (4/296, 1.4%), and Central and South America (4/296, 1.4%). France (70/296, 23.6%) and the United States (67/296, 22.6%) were the most represented countries, followed by Finland (36/296, 12.2%), Sweden (32/296, 10.8%), Denmark (28/296, 9.5%), and Norway (26/296, 8.8%; Figure 3 and Figure S5 in Multimedia Appendix 1). Most studies had a regional or local scope (177/296, 59.8%), in particular, traditional epidemiological studies, such as Agriculture and Cancer (AGRICAN) [207] and Agricultural Health Study (AHS) [172], which used AHDs to either identify potential individuals for inclusion or enrich their cohorts.

**Table 4:** Characteristics of the included studies (1975 to 2024; N=296).

Characteristic			Values
**Research goal, n (%)**			
	Study the association between farming and a health event	156 (52.7)	
	Study the association between individual characteristics and a health event	131 (44.3)	
	Other research goals	9 (3)	
**Study design, n (%)**			
	Retrospective cohort	129 (43.6)	
	Case-control study	62 (20.9)	
	Prospective cohort	56 (18.9)	
	Cross-sectional study	39 (13.2)	
	Ecological study	17 (5.7)	
	Multiple designs	10 (3.4)	
	Review	2 (0.7)	
	Perspective	1 (0.3)	
**Geographic scope, n (%)**			
	Nationwide	117 (39.5)	
	Regional or local	176 (59.5)	
**Temporal scope (y)**			
	Follow-up period, median (IQR)	9.50 (5-17)	
	Follow-up period, mean (SD)	12.8 (14.0)	
	Gap between the latest data used and publication year, median (IQR)	7.21 (5-9)	
	Gap between the latest data used and publication year, mean (SD)	7.21 (4.67)	
**Population, n (%)**			
	Adult	265 (89.5)	
	Adult and child	19 (6.4)	
	Child	8 (2.7)	
	Not reported	1 (0.3)	
**Sex, n (%)**			
	Female	130 (43.9)	
	Male	169 (57.1)	
	Female and male	188 (63.5)	
	Not specified	108 (36.5)	
**Participants, n (%)**			
	>1,000,000	50 (16.9)	
	100,001 to 1,000,000	53 (17.9)	
	10,001 to 100,000	65 (22)	
	1001 to 10,000	67 (22.6)	
	101 to 1000	47 (15.9)	
	10 to 100	8 (2.7)	
	Not reported	3 (1)	
**AHD** ^a^ **type, n (%)**			
	Disease register	158 (53.4)	
	Electronic health or medical record	124 (41.9)	
	Insurance claim	106 (35.8)	
	Population register	95 (32.1)	
	Hospital discharge databases	41 (13.9)	
**AHD use, n (%)**			
	Obtain information on sociodemographics	272 (91.9)	
	Obtain information on a health event	269 (90.9)	
	Identify a farmer	147 (49.7)	
	Identify an individual	140 (47.3)	
	Obtain information on occupations	117 (39.5)	
	Exposure assessment	57 (19.3)	
	Obtain information on a farming activity	43 (14.5)	
	Other uses	14 (4.7)	

^a^AHD: administrative health database.

Most studies included 1001 to 10,000 participants (67/296, 22.6%), followed by studies with 10,001 to 100,000 participants (65/296, 22%) and 100,001 to 1 million participants (53/296, 17.9%; Table 4). Larger studies (>1 million participants) accounted for 16.9% (50/296) of the included publications. Smaller studies, with 100 to 1000 participants, were less common (47/296, 15.9%), and very few (8/296, 2.7%) had <100 participants. Most studies included adult participants (284/296, 95.9%). Of 296 studies, 169 (57.1%) examined male individuals, 130 (43.9%) examined female individuals, and 188 (63.5%) examined both sexes, but 108 (36.5%) did not specify the participants’ sex.

More than half of the studies (156/296, 52.7%) aimed to explore the relationship between farming activities (eg, dairy farming) and health events, while 131 studies (44.3%) focused on individual characteristics, such as occupation, age, sex, and socioeconomic status (Figure S6 in Multimedia Appendix 1). Among those studies examining individual characteristics, farming was often considered broadly and compared to other occupations (95/131, 72.5%). Conversely, in studies investigating health outcomes specifically related to farming activities, agriculture was treated as a broad category in only 27.6% (43/156) of the cases. Most studies (277/296, 93.6%) used the general population or other nonfarming groups as the reference category without differentiating farmers by job role (eg, farm managers vs farm workers). Descriptive statistics and multivariable regression were the most commonly used methods. Notably, only 2 studies (2/296, 0.7%) incorporated artificial intelligence (AI) in their analysis [208,209].

Few studies investigated health outcomes in farmers’ family members or nonfarmers exposed to farming. Of 296 studies, only 3 (1%) focused on health events in farmers’ partners [177,210,211], 5 (1.7%) on farmers’ children [179,212-215], and 6 (2%) on nonfarmers exposed to farming-related risks [209,216-221]. There were 11 (3.7%) studies that explored health risks in migrant workers.

Some publications reported findings from the same cohorts (Figure S7 in Multimedia Appendix 1). The 10 most prolific cohorts included France-based AGRICAN (18/296, 6.1%) [207], the US-based AHS (17/296, 5.7%) [172], Nordic Occupational Cancer Study (NOCCA; 12/296, 4.1%) from Nordic countries (Finland, Denmark, Norway, Sweden, and Iceland) [189], France-based Tracking and Monitoring Occupational Risks in Agriculture (TRACTOR; 7/296, 2.4%) [222], and Cancer in the Norwegian Agricultural Population (7/296, 2.4%) [182] cohorts. Other notable cohorts included the US-based United Farm Workers of America (6/296, 2.0%) [223], France-based BALISTIC (5/296, 1.7%) [224], the international (29 countries) consortium agricultural cohort (AGRICOH; 4/296, 1.4%) [150,225], AIRBAg (4/296, 1.4%) from France [226], and the US-based National Agricultural Workers Survey (3/296, 1%) [227]. Among these top 10 cohorts, only NOCCA, United Farm Workers of America, and TRACTOR exclusively used AHDs.

### AHD Use

There was high heterogeneity in the coding systems used and the granularity of the information available regarding health events (outcomes), population, and exposure determinants, depending on the AHD and study considered. Regardless of the publication reviewed, AHDs and other datasets were never reported as adhering to the findable, accessible, interoperable, and reusable (FAIR) data principles [228-230]. In addition, none of them could be considered as FAIR data because, with a few exceptions [222], most AHDs were not precisely described, and data availability statements were rare. Furthermore, mainly due to privacy concerns, AHDs were not available for open and free access.

The most commonly used AHDs were disease registers, used in more than half of the studies (158/296, 53.4%), followed by electronic health or medical records (124/296, 41.9%), insurance claims (106/296, 35.8%), population registers (95/296, 32.1%), and hospital discharge databases (41/296, 13.9%; Table 4). Among disease registers, cancer (120/158, 75.9) and mortality registers (75/158, 47.5%) were the most frequently used (Figure S8 in Multimedia Appendix 1 and Multimedia Appendix 2). Nearly one-third of the studies (91/296, 30.7%) relied on a single AHD, with disease registers being the most common (38/91, 42%), followed by insurance claims (29/91, 32%), electronic health or medical records (18/91, 20%), population registers (5/91, 5%), and hospital discharge databases (1/91, 1%). Other types of digital data were used less frequently, including pesticide registration records (13/296, 4.4%), job-exposure matrices (JEMs; 12/296, 4.1%), crop-exposure matrices (11/296, 3.7%), pesticide use records (8/296, 2.7%), climate data (7/296, 2.4%), and air quality data (2/296, 0.7%). While contextual data were sometimes used (9/296, 3.0%), person-generated data, smart agriculture data, and omics were never used.

The AHDs and other digital data were primarily used to obtain sociodemographic information (272/296, 91.9%) and health event data (269/296, 90.9%). They were also used to identify farmers (147/296, 49.7%) or individuals (140/296, 47.3%), gather occupational information (117/296, 39.5%), assess exposure (57/296, 19.3%), obtain data on farming activities (43/296, 14.5%), or track climate conditions (7/296, 2.4%).

Nearly two-thirds of the studies (181/296, 61.1%) relied exclusively on digital data (AHDs or other), while more than one-third (112/296, 37.8%) incorporated self-reported information/active data (requiring active participant involvement) as part of epidemiological cohorts. A total of 111 (37.5%) out of 296 studies used participant-completed questionnaires (paper or electronic) to gather sociodemographic data and confounding factors (98/296, 33.1%), assess exposure (96/296, 32.4%), or collect health information (83/296, 28%). Some information was obtained through interviews (44/296, 14.9%) or clinical examinations (32/296, 10.8%). Biological monitoring (24/296, 8.1%) and airborne monitoring (2/296, 0.7%) were sometimes used, whereas no study reported dermal monitoring (Figure S9 in Multimedia Appendix 1).

Among all the AHDs used, the Mutualité Sociale Agricole (MSA) is a singularity. To the best of our knowledge, it is the only AHD specifically dedicated to the entire farming population of a country. Indeed, MSA is the French national insurance scheme that covers the entire farming workforce (5% of the overall French population) [115,128]. MSA was used in 60 studies (60/296, 20.3%). These studies were often part of cohorts with multiple publications, such as AGRICAN (18/60, 30%), TRACTOR (7/60, 12%), BALISTIC (5/60, 8%), AIRBAg (4/60, 7%), Aging Multidisciplinary Investigation (2/60, 3%) [151], BM3R (2/60, 3%) [231], FERMA (risk factors of the rural environment and allergic and respiratory disease; 1/60, 2%) [232], and Phytoner (1/60, 2%) [233]. Of these, TRACTOR was the only cohort using exclusively MSA data [222].

### Farming Exposure

A variety of exposure proxies were used to assess farming-related exposure. The most common proxy was a job title, which generally referred to whether the individual was a farmer (184/296, 62.2%). Other proxies included specific farming activities, such as dairy or crop farming (111/296, 37.5%), general pesticide exposure (yes or no; 62/296, 20.9%), and exposure to specific pesticide compounds (eg, glyphosate or paraquat) or pesticide classes (eg, insecticides; 51/296, 17.2%; Figure S10 in Multimedia Appendix 1 and Multimedia Appendix 2). The number of farming activities studied ranged from just 1 [226] to 78 [222], with an average of 8 farming activities per study. Similarly, the number of pesticide compounds assessed ranged from 1 [234] to 943 [235], with an average of 42 pesticides per study. Only 1 study investigated the mixture effect of exposure to multiple pesticide combinations on human health [236]. Investigations into other chemical exposures were also limited, with only 2 papers each addressing silica exposure [237,238] and air pollution [194,217] (2/296, 0.7%). Notably, no studies examined exposure to per- and polyfluoroalkyl substances or micro- and nanoplastics. Research on the broader farming exposome was rare (5/296, 1.7%) and typically used farming activities as proxies [152].

Of 296 studies, few explored exposure to physical agents, with 5 studies (1.7%) focusing on radiation [187,218,239-241]. No studies investigated the effects of climate change on farmers’ health. Exposure to biological agents was rarely studied as well, with just 3 (1%) out of 296 papers addressing mycotoxins [241-243]. Finally, only 3 studies (1%) examined psychological factors related to farming exposure [244-246].

### Health Events

The most frequently studied health events were cancer (142/296, 48%), followed by mortality (44/296, 14.9%), injuries (38/296, 12.8%), workplace accidents (32/296, 10.8%), respiratory disorders (30/296, 10.1%), neurodegenerative diseases (28/296, 9.5%), and mental health issues (26/296, 8.8%; Figure S11 in Multimedia Appendix 1 and Multimedia Appendix 2). Less frequently studied conditions included cardiovascular diseases (16/296, 5.4%), autoimmune disorders (11/296, 3.7%), musculoskeletal disorders (11/296, 3.7%), reproductive disorders (3/296, 1.0%), sleep disorders (1/296, 0.3%), and frailty (1/296, 0.3%). Notably, no studies explored the farming microbiome.

Among cancers, lung cancer was the most commonly investigated cancer (43/142, 30.3%), followed by prostate cancer (38/142, 26.8%), leukemia (37/142, 26.1%), colorectal cancer (35/142, 34.6%), multiple myeloma (35/142, 34.6%), non-Hodgkin lymphoma (35/142, 34.6%), bladder cancer (31/142, 21.8%), and brain cancer (31/142, 21.8%; Figure S12 in Multimedia Appendix 1). Respiratory disorders were primarily focused on asthma (15/30, 50%) and COPD (chronic obstructive pulmonary disease; 14/30, 47%). Parkinson disease was the most studied neurodegenerative condition (16/28, 57%), followed by multiple sclerosis (6/28, 21%). Fewer publications examined Alzheimer disease (2/28, 7%) and amyotrophic lateral sclerosis (2/28, 7%; Figure S13 in Multimedia Appendix 1). In the mental health field, suicide (12/26, 46%) and depression (8/26, 31%) were the most investigated issues (Figure S14 in Multimedia Appendix 1).

### Keyword Analysis

#### Overview

Following an initial extraction of 1259 authors’ keywords, manual harmonization was performed. Duplicate keywords were removed through singular or plural standardization (130/1259, 10.3%) and synonym unification and grouping of cancer-related terms (553/1259, 43.9%), yielding a final set of 576 (45.8%) harmonized keywords, which were all used in subsequent analyses to prevent selection bias.

On average, each publication included 8.90 keywords (90% CI 0-17), although 35 (11.8%) out of 296 publications lacked any keywords, in line with the journal guidelines. Keyword analysis confirmed prior findings regarding farming exposure and health outcomes. It also provided deeper insights into emerging research hot spots, directions, and gaps.

Of the total 576 keywords, 301 (52.3%) appeared only once, while 68 (11.8%) were mentioned at least 10 times. More frequently used keywords included 39 that appeared at least 20 times (39/576, 6.8%) and 11 that featured in at least 50 publications (11/576, 1.9%). The 50 most frequently used keywords were mentioned in at least 17 (5.7%) out of 296 publications, while the top 10 appeared in at least 51 publications (17.2%; Table S7 in Multimedia Appendix 1). The most frequently cited keyword was “cancer” (150/296, 50.7%), followed by “mortality” (96/296, 32.4%), “pesticide” (88/296, 29.7%), “occupation” (82/296, 27.7%), “farmer” (77/296, 26.0%), “agriculture” (74/296, 25%), “exposure” (57/296, 19.3%), and “epidemiology” (57/296, 19.3%).

In terms of overall citations, “cancer” (5766/9379, 61.5%), “pesticide” (3569/9379, 38.1%), and “mortality” (3097/9379, 33%) were the most cited keywords. During the past decade, the frequency of the top 5 keywords has drastically increased (Figure S15 in Multimedia Appendix 1). Notably, keywords such as “cancer,” “mortality,” “occupation,” “pesticide,” “agriculture,” and “farmer” have been consistently present in publications spanning at least 30 years (not necessarily consecutively; Figure S16 in Multimedia Appendix 1). In the last decade, emerging keywords, such as “big data,” “administrative health database,” “dust,” and “BMI,” have gained prominence (Figure S17 in Multimedia Appendix 1).

#### Keyword Co-Occurrence

**Figure 4:**
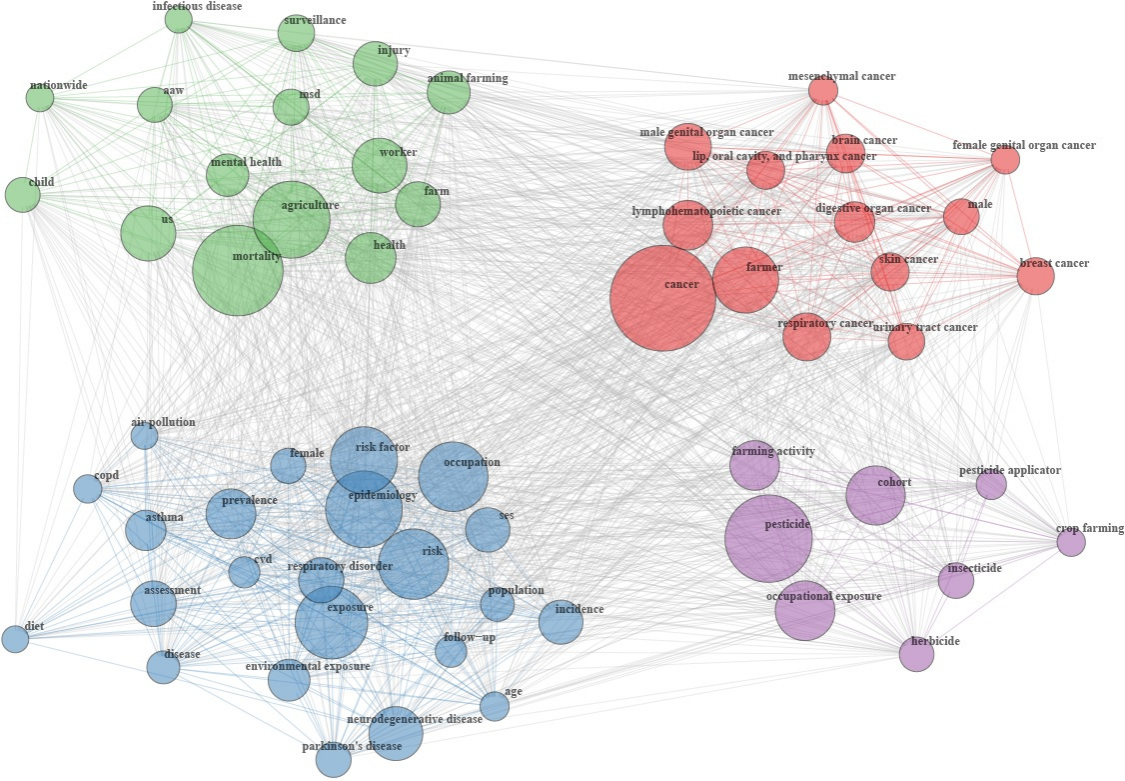
Keyword co-occurrence network of the 296 articles published between 1975 and 2024. Each vertex or node represents a keyword, while edges represent the co-occurrence between keywords. Two keywords are connected when they co-occur in the same publication, and the size of each vertex indicates the frequency of a keyword: larger vertices represent more frequently mentioned keywords. Keywords with the same color (cluster) represent a research area. AAW: workplace accident; COPD: chronic obstructive pulmonary disease; CVD: cardiovascular disorder; MSD: musculoskeletal disorder; SES: socioeconomic status.

A keyword co-occurrence network illustrating the frequency of keyword co-mentions in publications was constructed, thereby revealing relationships and conceptual connections (Figure 4). In this network, nodes or vertices represent keywords, with their sizes indicating frequency, while edges denote co-occurrences. The network’s density and arrangement reveal topic interconnectivity, with larger vertices representing more frequently mentioned keywords. The network visualization helps identify clusters of related topics and highlights core research areas.

Using a community detection algorithm (spin-glass model with simulated annealing), 4 distinct clusters or communities of keywords were identified. Each cluster groups keywords that are often mentioned together, with stronger internal associations and weaker connections to keywords in other clusters.

The most frequently used keywords for each cluster were “cancer” (red cluster), “pesticide” (purple cluster), “mortality” (green cluster), and “exposure” (blue cluster). The red cluster highlights associations between various types of cancer, reflecting the fact that studies investigating cancer risks often examine multiple types of cancer. The green cluster links “mortality” with terms such as “mental health,” “injury,” and “animal farming,” explained by the association between workplace accidents, mental health issues (eg, suicide), animal farming, and mortality. In the purple cluster, “pesticide” connects with “occupational exposure” and “farming activity,” emphasizing that pesticide exposure is primarily studied in occupational settings across different types of farming. The blue cluster connects “exposure” to terms such as “neurodegenerative disease,” “respiratory disorder,” “cardiovascular disorder,” “risk factor,” “air pollution,” “age,” and “diet,” indicating the study of various risk factors in relation to several health events. These clusters highlight current research hot spots that focus on 4 main interconnected themes: the associations between risk factors, pesticide exposure, farming activities, and a range of diseases.

#### Thematic Mapping: Research Hot Spots

**Figure 5:**
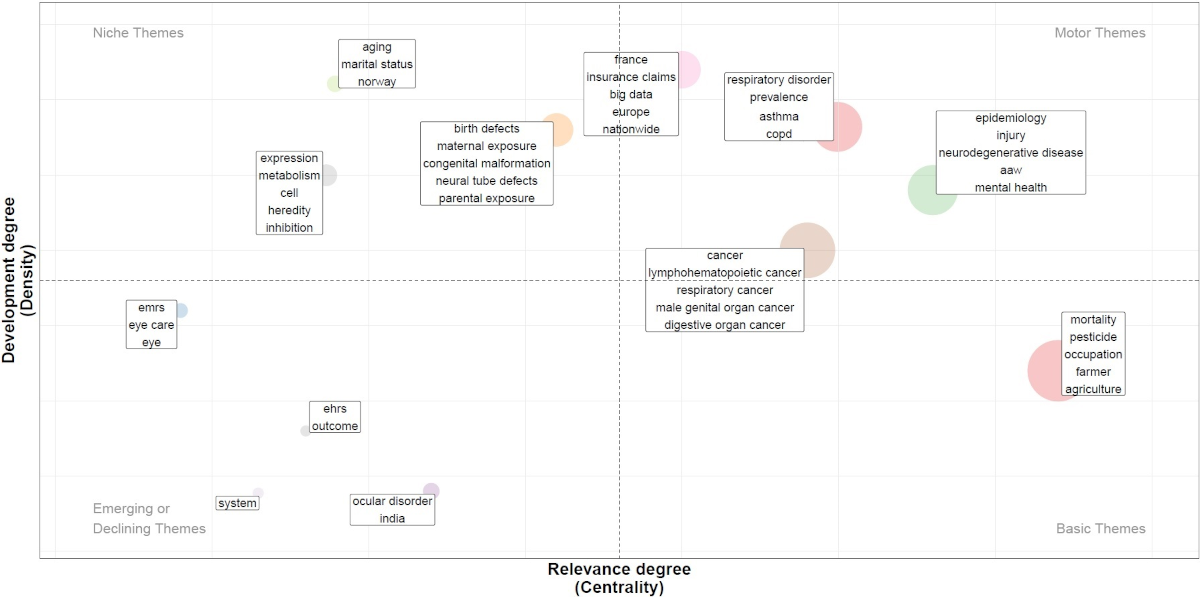
Thematic mapping: research hot spots based on keywords from the 296 articles published between 1975 and 2024. The graph shows how themes relate to each other and their relevance within a specific field. This graph is divided into 4 quadrants, categorizing topics based on their relevance (x-axis and Callon centrality) and maturity (y-axis and Callon density) within the broader research landscape. Each circle represents a theme or topic (ie, a cluster of equivalent keywords), with the circle size corresponding to the frequency of the keywords associated with that theme. AAW: accident at work; COPD: chronic obstructive pulmonary disease; EHR: electronic health record; EMR: electronic medical record.

Figure 5 presents a thematic map that illustrates current research directions. Thematic mapping visualizes the relationship between research themes or topics, enabling the identification of directions, emerging areas, and gaps in the literature. The result is a strategic diagram that shows how themes relate to each other and their relevance within a specific field. The graph is divided into 4 quadrants, categorizing topics based on their relevance (x-axis, Callon centrality) and maturity (y-axis, Callon density) within the broader research landscape. Each circle represents a theme or topic (ie, a cluster of equivalent keywords), with the circle size corresponding to the frequency of the keywords associated with that theme.

The upper-right quadrant represents “hot topics,” which are both highly relevant and mature in the research landscape. Four key hot topics drive AHD-based public health research in farming populations. These include 1 topic focused on cancer research; another on respiratory disorders; and a third encompassing neurodegenerative diseases, workplace accidents, injuries, and mental health issues. The final hot topic involves large-scale studies in France and Europe using big data and insurance claims.

The lower-right quadrant contains “basic topics,” which are relevant but not yet mature in the research landscape. Only 1 such theme emerged: research related to pesticide exposure, mortality, and farming.

In the upper-left quadrant, “niche themes” refer to mature research topics that have not yet achieved full relevance. Three niche themes were identified: the first involves studies examining aging and research conducted in Norway; the second focuses on reproductive disorders and parental exposure, a theme poised to potentially evolve into a hot topic; and the final niche theme covers genetics and metabolism.

Finally, the lower-left quadrant contains “peripheral topics,” which represent either emerging or declining themes with low relevance and maturity. Four peripheral topics were observed, of which 2 (50%) were primarily centered on research on ocular disorders, 1 (25%) on the use of electronic health or medical records, and 1 (25%) on studies conducted in India.

This thematic map helps highlight both well-established and emerging areas of research, as well as gaps that may be ripe for future investigation.

#### Bibliometric Farming Exposome

To identify research directions and gaps in the farming exposome literature, a bibliometric keyword co-occurrence analysis was conducted to explore the farming exposome by examining the co-occurrence between keywords associated with potential risk factors and specific health outcomes. This analysis was restricted to exposome-related and health event–related keywords. Of 576 keywords, 130 (22.6%) were related to the exposome, among which 93 (16.1%) were related to the specific external exposome (eg, pesticide), 19 (3.3%) to the general external exposome (eg, climate), and 18 (3.1%) to the internal exposome (eg, oxidative stress). Furthermore, there were 70 (12.2%) health event–related keywords (eg, brain cancer).

The results of this analysis are provided in Tables 5 and 6 and Multimedia Appendix 3, with each cell representing the percentage of occurrences of an exposome-related keyword (eg, air pollution) in all publications mentioning a specific health event–related keyword (eg, Alzheimer disease). For example, a value of 33.3 indicates that an exposome-related keyword appeared in 33.3% of all publications mentioning a specified health event–related keyword. To facilitate interpretation and ease the reading of Tables 5 and 6, exposome-related keywords were categorized into 19 groups (eg, chemical agent) and health event–related keywords into 20 groups.

**Table 5:** Co-occurrence between keywords related to internal exposomes and health event categories among the articles published between 1975 and 2024. Each cell refers to the number of times (%) a keyword related to an exposome category (eg, chemical agent) was mentioned among all publications in which a keyword related to a health event category (eg, cancer) appeared (N=296). Please note that the absolute value for each row is provided in parentheses with the row header and remains the same for all the parameters in that row.

Health event, n	Internal exposome (%)										
	Age	Sex	BMI	BP^a^	Heredity	Ethnicity	Hormone	Menopause	Metabolism	OS^b^	Inflammation

Cardiovascular disease (n=17)	5.88	11.8	5.88	5.88	0	5.88	0	0	0	0	0
Work-related disease (n=5)	0	20	0	0	0	0	0	0	0	0	0
Autoimmune disease (IBD^c^, RA^d^, vasculitis, and NR^e^; n=9)	0	0	11.1	0	0	0	0	0	0	0	0
Cancer (n=150)	3.62	27.5	2.17	0	0.73	1.45	3.62	0.73	2.9	0.73	0
Dental health (n=2)	0	0	0	0	0	0	0	0	0	0	0
Ocular disorder (n=6)	16.7	0	0	0	0	0	0	0	0	0	0
Frailty (n=2)	50	0	50	0	0	0	0	0	0	0	0
Anemia (n=1)	100	100	0	0	0	0	0	0	100	0	0
Infectious disease (malaria, Lyme disease, tuberculosis, toxoplasmosis, and NR; n=14)	0	7.14	0	0	0	7.14	0	0	0	0	0
Injury (including workplace accident and disability; n=40)	4.26	8.51	2.13	0	0	2.13	0	2.13	0	0	0
Chronic kidney disease (n=3)	0	0	0	0	0	0	0	0	0	0	0
Mental health disorder (depression, suicide, and NR; n=25)	9.52	4.76	4.76	0	0	0	0	0	0	0	0
Metabolic disorder (diabetes, dysthyroidism, and NR; n=9)	11.1	0	11.1	11.1	0	11.1	0	0	0	0	0
Mortality (n=75)	4.17	17.7	0	0	1.04	0	1.04	2.08	0	0	0
Musculoskeletal disorder (arthritis, low-back pain, and NR; n=14)	7.14	14.3	7.14	0	0	0	0	0	0	0	0
Neurodegenerative disease (AD^f^, ALS^g^, MND^h^, MS^i^, PD^j^, and NR; n=33)	6.06	0	0	0	3.03	6.06	0	0	12.1	6.06	0
Sensory impairment (n=1)	0	0	0	0	0	0	0	0	0	0	0
Reproductive disorder (birth defects, infertility, spontaneous abortion, and NR; n=24)	0	28.6	14.3	0	0	7.14	7.14	0	7.14	0	0
Respiratory disorder (allergy, asthma, COPD^k^, pneumonia, sarcoidosis, and NR; n=39)	5.88	11.8	8.82	2.94	0	5.88	0	0	5.88	0	2.94
Skin disorder (dermatitis and NR; n=2)	0	50	0	0	0	0	0	0	0	0	0

^a^BP: blood pressure.

^b^OS: oxidative stress.

^c^IBD: inflammatory bowel disease.

^d^RA: rheumatoid arthritis.

^e^NR: not reported.

^f^AD: Alzheimer disease.

^g^ALS: amyotrophic lateral sclerosis.

^h^MND: motor neuron disease

^i^MS: multiple sclerosis.

^j^PD: Parkinson disease.

^k^COPD: chronic obstructive pulmonary disease.

**Figure 6:**
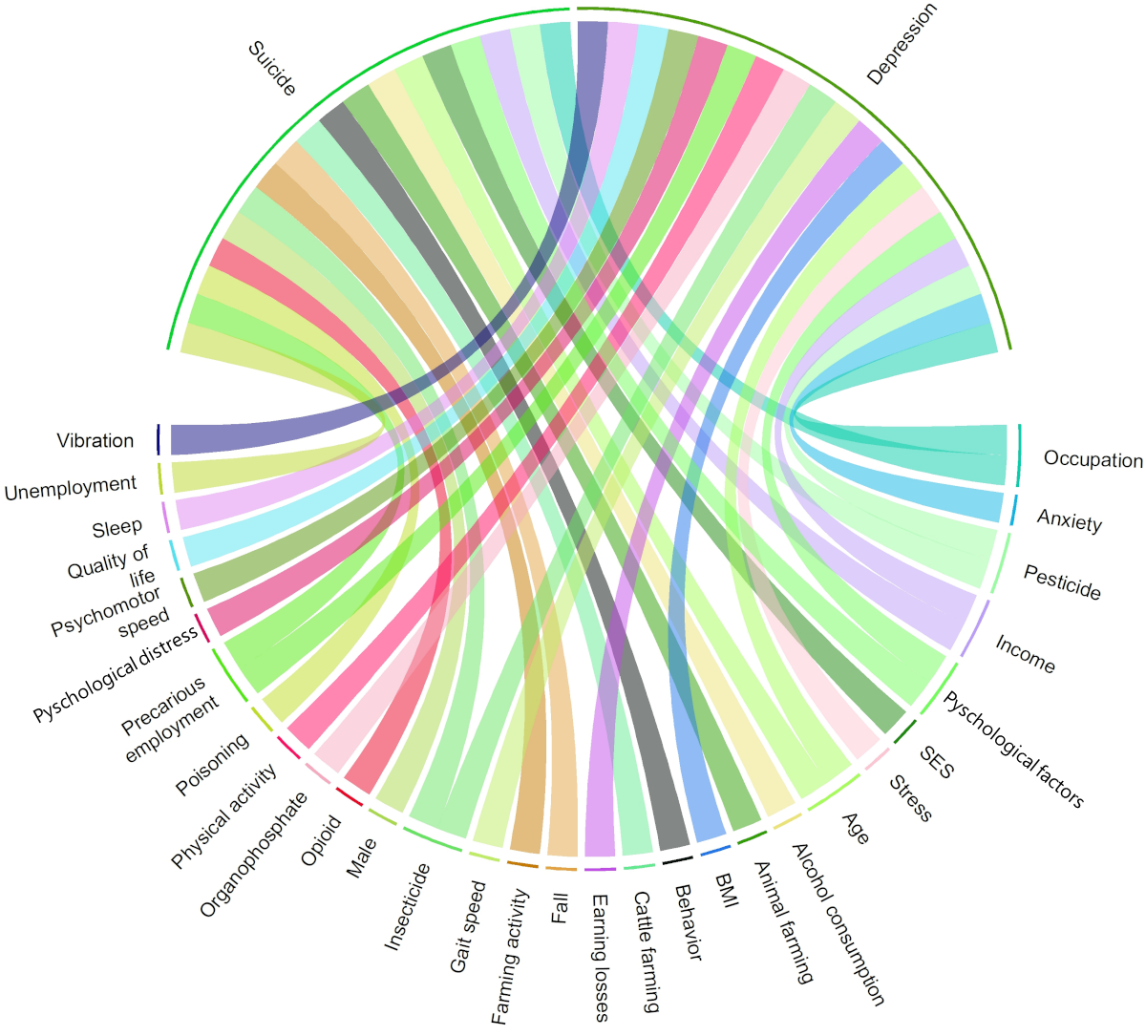
Chord diagram of keyword co-occurrence between potential risk factors and mental health disorder keywords from the 296 articles published between 1975 and 2024. Disease-related keywords are displayed on the top half of the chord diagram, while exposome-related keywords are displayed on the bottom half. Each chord or link indicates that an exposome-related keyword was mentioned with a disease-related keyword (co-occurrence) at least once in the same publication. The chord color differs from one exposome-related keyword to another. SES: socioeconomic status.

Distinct keyword exposome profiles were developed for each health event–related keyword (Figures S18-S43 in Multimedia Appendix 1), as illustrated in Figure 6 for mental health disorders. Most exposome-related keywords associated with keywords related to mental health disorders pertained to the type of occupations as well as chemical, lifestyle, socioeconomic, and psychological factors. Cancer-related keywords were associated mostly with keywords related to the internal (sex) and specific external exposome (chemical agents, lifestyle, and type of occupations). Autoimmune disease–related keywords co-occurred mostly with external exposome–related keywords (chemical agents, lifestyle, type of occupations, and socioeconomic factors). Neurodegenerative disease–related keywords were associated mostly with keywords related to the specific external exposome (lifestyle, chemical agents, and type of occupations). Reproductive disorders co-occurred mostly with internal (sex and BMI) and specific external exposome–related keywords (chemical agents and type of occupations). Keywords related to both musculoskeletal disorder and injury were associated with keywords from all 3 exposome components, in particular sex, type of occupations, lifestyle, biomechanical factors, chemical agents, and psychological factors. Infectious disease–related keywords co-occurred with specific external exposome–related keywords (biological agents and type of occupations), while respiratory disorder–related keywords were associated mostly with internal (sex) and specific external exposome–related keywords (lifestyle, chemical and biological agents, and type of occupations). Cardiovascular disorder–related keywords were associated with keywords from all 3 exposome components, in particular the sex, type of occupations, lifestyle, chemical agents, and socioeconomic and psychological factors.

**Table 6:** Co-occurrence between keywords related to specific external and general external exposomes and health event categories among the articles published between 1975 and 2024. Each cell refers to the number of times (%) a keyword related to an exposome category (eg, chemical agent) was mentioned among all publications in which a keyword related to a health event category (eg, cancer) appeared (N=296). Please note that the absolute value for each row is provided in parentheses with the row header and remains the same for all the parameters in that row.

Health event, n	Specific external exposome (%)						General external exposome (%)		
	Lifestyle	CA^a^	BA^b^	BF^c^	Occupation	PA^d^		SF^e^	PF^f^

Cardiovascular disease (n=17)	11.8	29.4	0	11.8	23.5	0		29.4	17.6
Work-related disease (n=5)	0	20	20	20	40	0		20	0
Autoimmune disease (IBD^g^, RA^h^, vasculitis, and NR^i^; n=9)	22.2	44.4	0	0	55.6	0		22.2	0
Cancer (n=150)	17.4	51.4	7.25	1.45	58.7	5.8		8.7	0
Dental health (n=2)	50	0	0	0	0	0		50	0
Ocular disorder (n=6)	16.7	16.7	0	0	0	0		0	16.7
Frailty (n=2)	100	50	0	0	0	0		0	50
Anemia (n=1)	0	0	0	0	0	0		0	0
Infectious disease (malaria, Lyme disease, tuberculosis, toxoplasmosis, and NR; n=14)	21.4	21.4	50	7.14	28.6	0		14.3	7.14
Injury (including workplace accident and disability; n=40)	12.8	14.9	2.13	14.9	23.4	2.13		4.26	6.38
Chronic kidney disease (n=3)	0	100	66.7	0	0	33.3		0	0
Mental health disorder (depression, suicide, and NR; n=25)	28.6	23.8	0	4.76	33.3	4.76		23.8	28.6
Metabolic disorder (diabetes, dysthyroidism, and NR; n=9)	33.3	44.4	0	11.1	44.4	11.1		0	22.2
Mortality (n=75)	17.7	55.2	8.33	4.17	54.2	5.21		17.7	2.08
Musculoskeletal disorder (arthritis, low-back pain, and NR; n=14)	14.3	21.4	7.14	14.3	42.9	7.14		0	21.4
Neurodegenerative disease (AD^j^, ALS^k^, MND^l^, MS^m^, PD^n^, and NR; n=33)	21.2	48.5	6.06	0	36.4	0		9.09	3.03
Sensory impairment (n=1)	0	0	0	0	100	0		0	0
Reproductive disorder (birth defects, infertility, spontaneous abortion, and NR; n=24)	7.14	35.7	7.14	0	64.3	0		7.14	0
Respiratory disorder (allergy, asthma, COPD^o^, pneumonia, sarcoidosis, and NR; n=39)	47.1	35.3	11.8	2.94	35.3	0		8.82	5.88
Skin disorder (dermatitis and NR; n=2)	50	50	0	0	50	0		0	0

^a^CA: chemical agent.

^b^BA: biological agent.

^c^BF: biomechanical factor.

^d^PA: physical agent.

^e^SF: socioeconomic factor.

^f^PF: psychological factor.

^g^IBD: inflammatory bowel disease.

^h^RA: rheumatoid arthritis.

^i^NR: not reported.

^j^AD: Alzheimer disease.

^k^ALS: amyotrophic lateral sclerosis.

^l^MND: motor neuron disease

^m^MS: multiple sclerosis.

^n^PD: Parkinson disease.

^o^COPD: chronic obstructive pulmonary disease.

## Discussion

### Principal Findings

This review provides the first comprehensive and objective synthesis of research on the use of AHDs to address health issues in farming populations. It identifies major contributors, key publications, and existing research gaps while also suggesting future directions for leveraging AHDs to study health issues in farming populations. Overall, findings indicate that only a small part of the exposome and a limited range of health events have been examined within farming populations through the reuse of AHDs.

### Current Directions in AHD Use for Public Health Research in Farming Populations

Research using AHDs in farming populations has been predominantly conducted in developed countries [150,225], with the United States [172,223,227]; France [207,222,224]; Canada [145,186,195]; and Scandinavian nations [182,189], including Denmark, Finland, Norway, and Sweden, leading the field. This dominance is linked to considerable funding from these regions and international collaborations. Scandinavian countries are particularly advanced in AHD use, offering databases that are highly complete, accessible, and well-integrated into public health research. AHDs from Denmark, Sweden, Canada, and France also provide comprehensive data on a patient’s digital trajectory within their respective health systems [93,98,104,108,113,115,128,247]. France stands out further, with an AHD dedicated specifically to the entire farming population (MSA). This may explain the frequency of large-scale and long-term studies from these countries, some of which included >100,000 participants. However, many studies still had a regional focus, partly due to the use of AHDs by traditional epidemiological studies such as AGRICAN [207] and AHS [172], which rely on limited resources [47-50,53,54,57]. These studies often used AHDs to identify farming populations for inclusion or to supplement cohort data. The international AGRICOH consortium, initiated by the US National Cancer Institute and the International Agency for Research on Cancer, includes 11 (38%) of the 29 cohorts identified in this review [150,225]. However, several cohorts in AGRICOH were not identified, potentially due to lack of publications, language barriers, or limited use of AHDs. There were many publications associated with these well-established cohorts, for which many of the most prolific authors were working [172,182,189,207,222-227].

The most frequently used AHDs in farming population health research were disease registers, followed by electronic health or medical records and insurance claims. More than two-thirds of the studies used disease registers, in particular, cancer and mortality registers. This is not surprising because disease registers are created for clinical and research purposes with a continuous, exhaustive, and optimal digital collection of individual data regarding ≥1 health event in a geographically defined population [53,105]. The coding systems and the granularity of information related to health outcomes, populations, and exposure determinants varied widely across studies. Most studies (291/296, 98.3%) used AHDs to collect sociodemographic and health event information [222].

There was no consensus on the best methods or proxies to assess farming exposure. A variety of exposure proxies and determinants were used across studies, with indirect methods being the most common. Many studies (237/296, 80.1%) dichotomized proxy, for example, classifying individuals as farmers or nonfarmers or as pesticide-exposed versus nonexposed. In nearly two-thirds of the included studies, job title (ie, “being a farmer”) served as the primary exposure proxy. About one-third of studies took into account specific farming activities (eg, dairy farming and crop farming) to reflect the diversity of farming practices. This approach is a valuable proxy for agricultural exposure, offering a broader representation of the farming exposome, which involves multiple stressors beyond just pesticides [147,152,188,201,248,249]. Farming activity information was often derived from digitalized data, such as agricultural censuses or self-reported data from mandatory insurance enrollments [152]. Many studies (111/296, 37.5%) combined AHDs with self-reported data (eg, questionnaires) [172,207], which allowed for more comprehensive data collection but tended to restrict the scope to regional studies due to resource constraints. These studies typically yielded high-quality data, with more potential confounders considered compared to studies relying solely on AHDs. Most studies (68/111, 61.3%) using self-reported data focused on single exposures, mainly pesticides, with only 1 study addressing multiexposure to pesticides [236]. Biological monitoring and airborne chemical sampling were rarely conducted, likely due to practical and financial constraints and the short half-lives of most pesticides [250]. Dermal chemical monitoring has not been reported, even though it is the main exposure route for pesticides [251]. The high number of studies investigating exposure to pesticides may be explained by the fact that AHS focuses on pesticide applicators and their spouses [172] and because many pesticides have adverse health effects on humans, such as neurotoxicity or endocrine disruption [252-256]. Beyond pesticides, farmers face exposure to other chemicals [257], such as air pollution; micro- and nanoplastics [258-263]; biological agents (eg, endotoxins and zoonoses) [264-266]; physical agents (eg, UV radiation, noise, and vibrations) [187,267]; biomechanical factors (eg, repetitive movements, heavy load, and working posture) [198,268,269]; and psychosocial stressors [270-274], which have been less studied than pesticides. Despite these multiple exposures, the broader farming exposome remains understudied.

In addition to AHDs, some studies integrated other secondary data, such as climate data [187,275], air quality data [194,217], JEMs [212,276,277], or crop-exposure matrices [278]. JEMs provide exposure level estimates for various chemicals and stressors based on job categories [250,279]. Although JEMs can provide valuable exposure data, they often lack the specificity of individual-level data, making it difficult to account for task-specific risks, temporal variations, and the inclusion of specific worker subgroups such as female individuals [250,279-282]. The lack of a universal standard for JEMs further complicates their application, which may explain why many studies still rely on self-reported data for more accurate exposure assessment despite the risk of recall bias [250,279-282].

The health outcomes studied were predominantly cancer [145,150,151,171-175,177-179,182,183,185,189,190,196,207,210-212,223], mortality [173,186,194,195,202,205], workplace injuries [198,208], respiratory disorders (eg, asthma and COPD) [151,180,181,213,224,226], neurodegenerative diseases (eg, Parkinson disease) [111,151,176,184,187,188,193,197,201, 248,249,283], and mental health issues [151,244,273], which represent focal points within the research field. This is not surprising because many well-established cohorts centered on cancer research, in particular AGRICAN [207], Cancer in the Norwegian Agricultural Population [182], and NOCCA [189]. In addition, arsenic and inorganic arsenic compounds are classified as carcinogenic to humans by the International Agency for Research on Cancer [252,253,284], while malathion, glyphosate, diazinon, dichlorodiphenyltrichloroethane, and occupational exposures in spraying and application of nonarsenical insecticides are classified as probably carcinogenic to humans (group 2A), and several other pesticides are ranked as possibly carcinogenic to humans (group 2B), such as tetrachlorvinphos and parathion. Regarding mortality, it is often cancer and suicide mortalities that are investigated [186,195,202,244]. Furthermore, several pesticides are neurotoxic [252,255], but existing studies focused mainly on Parkinson disease and multiple sclerosis, with a paucity of data on Alzheimer disease and other neurodegenerative diseases [147,283]. In contrast, certain areas, such as cardiovascular diseases [151,194,203,227,285,286], autoimmune disorders [168,219,237], musculoskeletal disorders [192,204,245,287,288], reproductive disorders [242,289,290], sleep disorders [191], aging-related conditions [151,291], hearing impairment [267,292], and the microbiome [8,293-298], remain underexplored, despite their potential relevance to farming populations.

### Challenges of Reusing AHD for Public Health Research in Farming Populations

Each AHD presents unique advantages and limitations. For example, large sample sizes and a large number of available health events are strengths, while generalizability and the absence of key confounders are challenges [64,93,95,105,115]. Access to AHDs is frequently restricted by a variety of factors, including governance and technical barriers, such as language, data structure, interoperability, and coding systems. Additional challenges stem from the type of AHD (eg, insurance claims or cancer registers), inadequate documentation (eg, absence of a data dictionary), limited accessibility due to costs or conditions, and jurisdictional and legal constraints [30,62,64,81,113,115]. Identifying the optimal AHD for a given research question is also complex, especially when considering the heterogeneity in coding systems and country-specific data structures. In countries such as Scandinavia, Canada, and France, individual identifiers facilitate data linkage across multiple AHDs, enhancing research opportunities [93,104,113,115,128,247]. However, many AHDs are not ready for research and require significant processing, cleaning, and understanding before they can be analyzed [93,105,113,222,299]. Another major challenge is the long lag between data access, analysis, and research publication. On average, studies used data that were 7 years old at the time of publication, largely due to delays in data access, administrative approvals, and the need to prepare complex datasets for analysis [223,300]. For instance, the TRACTOR project took 2 years to clean and prepare its dataset for research use [222]. These delays are compounded by the time required to conduct statistical analyses and prepare manuscripts for publication, as well as review and publication times (delay from submission to acceptance and from acceptance to publication). Another limitation of AHDs is the lack of detailed exposure data. AHDs rarely include exposure information due to their administrative focus, requiring researchers to supplement with additional data sources, such as JEMs or self-reported data. When exposure information is recorded in AHDs, it is often too generalized, typically only reflecting broad job classifications, such as farming, without specifying detailed activities or stressors. There are some exceptions, such as MSA data, which capture a wider range of specific farming activities (eg, dairy farming) [222]. However, exposure to specific stressors (eg, chemical compounds) is largely absent from AHDs.

The reference populations used in farming studies vary, which precludes direct comparisons and limits the generalizability of the findings. For example, AGRICAN used the general population as a reference [207], while TRACTOR used a farming population [152,168]. Furthermore, studies differ in their focus on specific farming populations, such as the entire agricultural workforce [207], farm managers [152], or pesticide applicators [172], which may lead to distinct exposure profiles that influence health outcomes because these farming populations have different socioeconomic status, experiences, and behaviors. Hence, to avoid or lessen bias, some studies focused on specific farming populations [152,172]. Moreover, the scope of farming populations included in studies is often limited, omitting subgroups, such as farm families, nearby residents, or consumers exposed to agricultural products, which limits the broader application of the findings. In addition, farming practices can vary significantly between countries and studies, and there is no international standardized classification for farming activities. In some cases, farming categories are derived from legal or administrative sources, as seen in the MSA data [152,168,283]. This lack of standardization limits the comparability and generalizability of findings across studies. In addition, the generalizability of the findings to other countries when using AHDs may be limited because of the differences in health care systems [93].

There are several well-known limitations of AHDs that complicate the investigation of health outcomes [301]. Health events captured in AHDs are typically limited to those requiring medical attention, which may not reflect the true incidence of diseases. In addition, the level of detail varies across diseases, even within the same AHD [92]. Although diagnostic accuracy is generally higher in disease registers, these are often geographically limited and cover only a subset of health conditions. For example, in France, cancer registers only cover 23 (24%) out of 96 administrative regions [155]. In addition, certain conditions, such as depression, are not covered by any registers. Identifying health outcomes in AHDs often requires complex algorithms that combine data from multiple sources, such as drug reimbursements, disease declarations, or medical procedures [104,105,128,152,302-304]. In addition, inconsistencies in case definitions and algorithms across studies and countries hinder the ability to compare and pool risk estimates [104,302,304]. Some AHDs also lack critical clinical information, such as laboratory results and genetic data [115,128,305], and the recorded diagnosis or treatment date may not correspond to the actual onset of the disease. Furthermore, diagnosis codes are not always indicative of a confirmed diagnosis.

### Emerging Opportunities and Research Needs

While AHDs are well-used in certain countries, there are underexplored opportunities in regions such as the United Kingdom, where AHDs exist but are underused for research [113]. For low- and middle-income countries, the development and access to AHDs remain limited, and international support is needed to expand this research infrastructure. As already reported by Habib et al [306], there is a notable lack of sex-specific data, even though occupational exposures and health outcomes can vary significantly between sexes due to genetic, physiological, psychological, and behavioral factors [307-311]. Future research should address these disparities to provide a more comprehensive understanding of health risks in farming communities. Although there are inherent delays in using AHDs due to the time required for data generation, consolidation, and access, we advocate for the continued publication of studies, even those using older data. Historical data remain vital for better understanding long-term health patterns, particularly for diseases such as cancer, where tumor initiation can span decades [312,313]. Editors should encourage the publication of studies using older datasets, especially when addressing long-term health outcomes (eg, cancer and neurodegenerative diseases) or when recent data are not available [312].

None of the analyzed AHDs fully adhere to the FAIR principles, possibly because most were developed before the establishment of these principles in 2016 [228-230]. Moreover, the assessment of FAIR compliance of AHDs relied solely on information presented in publications, which may not provide a comprehensive evaluation. Nevertheless, there is a critical need to advocate for the integration of FAIR principles within AHDs to enhance public health research [228-230]. Currently, the landscape is favorable for data reuse, particularly with initiatives such as the forthcoming European Health Data Space [314-316]. Data reuse extends beyond mere access; it encompasses data discovery, a fundamental aspect of FAIR principles that involves recognizing the existence of databases [228-230]. To facilitate this, the creation of data catalogs is essential [228]. Numerous data repositories, such as Re3Data [317], Zenodo [318], CANUE [319], Figshare [320], “Epidémiologie – France” [321], data.gouv [322], Dataverse [320], or Data Europa [320], already exist. In addition, specialized multidisciplinary open-access and peer-reviewed journals such as *Scientific Data* and *Data in Brief* publish datasets [318]. A dataset search can also be conducted using the Google (Google LLC) platform [323]. However, the documentation and access conditions for datasets can highly vary across inventories, complicating the selection process for researchers. The absence of indicators or scores for data reusability further hampers efforts to identify the most suitable datasets for specific research questions [45,69]. To our knowledge, no comprehensive catalog of AHDs currently exists to date. A web-based inventory of AHDs, modeled after existing resources, such as OccupationalCohorts.net [324], OccupationalExposureTools.net [325], and Toxicological and Exposure Database Inventory [10], could greatly enhance research endeavors. The motivation for analyzing AHDs often stems from the data they contain. Consequently, as data availability increases, researchers will be better positioned to formulate research questions and engage in a parallel process of “datagraphy” or “datagraphic search” akin to traditional bibliographic research. The objective of datagraphy would be to determine which datasets are best suited for addressing specific research questions, highlighting the need for accessible catalogs to support this goal.

There is also an opportunity to integrate other secondary data, such as person-generated data (eg, mobile health, social media, digital footprints, and wearable sensors); contextual data (eg, climate and air quality); and smart agriculture data [2,83,84,326-328]. These datasets, largely untapped in farming population research, could provide new insights into health outcomes and environmental exposures [101].

Nationwide studies using big data were a hot spot. AI, such as machine learning, is particularly useful for analyzing big data and holds substantial promise for future research [329-331], particularly for identifying predictors of health outcomes in farming populations [332]. To date, AI has been underused, with only 2 studies using it, 1 identifying occupational injuries in agriculture [208] and 1 reviewing the development of chronic kidney disease risk prediction models [209]. Incorporating AI, along with cohort enrichment and interdisciplinary expert interpretation, could open new avenues for research.

Many studies continue to examine agriculture as a broad category, highlighting the need for more detailed investigations into specific farming activities and tasks [147,152,188,201,248,249]. Our analysis reveals major research gaps in understanding environmental and occupational exposures among farming populations, particularly with regard to emerging concerns such as per- and polyfluoroalkyl substances, biological agents, micro- and nanoplastics, and the impact of climate change. Climate change is a critical issue for agriculture, as it may drive shifts in pests, diseases, and farming practices [274,333-339]. Parental exposure appears to be a theme that will soon become a hot topic. Furthermore, research is needed to explore the farming exposome, particularly focusing on the “mixture effects” of multiple simultaneous exposures [340,341]. Omics data, which have not been used in farming population studies to date, represent a promising avenue for future research because genetics and metabolism were found to be a niche theme. Omics data refer to the large-scale datasets generated from various omics technologies that analyze biological molecules (eg, genomics, transcriptomics, proteomics, and metabolomics), which provide comprehensive insights into different biological layers and processes [11,342,343].

To enhance the characterization of farming exposome research using keyword analysis, there is a pressing need for standardized keyword reporting. We advocate for the development of a standardized approach to reporting keywords in scientific journals, including defining a minimum set of information (eg, study type, health outcome, population studied, data sources, and positive, negative, or null associations) and adopting a list of standardized terms. Although challenging, this approach would improve literature searches, make data more comparable and FAIR [228-230], and lead to more efficient, frugal (less time and energy spent to identify relevant information), and accurate synthesis of the scientific literature, such as in reviews and bibliometric analyses.

The prominence of topics such as cancer, neurodegenerative diseases, mortality, injuries, and mental health issues underscores the need for targeted prevention strategies. The thematic map analysis indicates that reproductive disorders (eg, birth defects, endometriosis, and infertility) are on the verge of becoming a central research focus. Emerging and understudied health conditions, including ocular disorders, autoimmune diseases (eg, inflammatory bowel disease and rheumatoid arthritis), sleep disorders (eg, sleep apnea), cardiovascular diseases, and musculoskeletal conditions (eg, low-back pain), warrant increased attention and further research. Aging-related health issues, such as frailty, also represent promising avenues for future research, particularly given the growing aging population and associated health care challenges [24].

### Limitations

The findings of this review should be considered in view of their limitations. Because of time and resource constraints, a single screening approach was used, with only 1 author (PP) conducting the review and bibliometric analysis. While single screening is an efficient use of time and resources, there is a higher risk of missing relevant studies than when using dual screening [344,345]. However, when completed by an experienced reviewer familiar with the research topic, the proportion of missed studies is limited and estimated to be around 3% [344]. Therefore, we cannot exclude the possibility that some studies may have been missed. Nevertheless, we are confident that none of these methodological limitations would change the overall conclusions of this work. Our restriction on articles published in English and French may have inadvertently excluded potentially relevant publications. We cannot exclude the possibility that publications using AHDs for addressing health issues in farming populations may have been missed if there was no mention of AHD in the publications’ titles and abstracts. However, it is important to mention that our search strategy was similar to recent reviews that specifically examined the use of AHDs for population-based research [93,96,103,104]. We further broadened our search by including synonyms to improve the comprehensiveness of our literature search. Some details and specificities on the AHDs and other digital data used may be limited because only information reported in each study was used. Shortcomings inherent to bibliometric analysis cannot be excluded. Some authors may have duplicate names, and namesakes could exist. This limitation could not be prevented as a unique author identifier (eg, Open Researcher and Contributor ID number) was not available. Self-citation could not be identified.

While our keyword analysis helped map the farming exposome in AHD-based public health research, this profile is incomplete and potentially biased. Because our review focused on AHD-based studies, we likely missed relevant epidemiological studies not using AHDs, leaving gaps in our understanding of the complete farming exposome across public health. In addition, the variability in keyword reporting practices across journals introduced bias into our keyword analysis. Some journals limit the number of keywords, and the lack of standardized keyword ontologies adds further variability. To mitigate this bias, we manually harmonized the keywords (eg, the use of 1 unique term for a given entity). While this approach is time-consuming, it allows for a more accurate analysis. For instance, if this approach was not performed, the same entity could be designated by various terms that would have been considered separate entities or terms, potentially resulting in underestimates (eg, in the number of publications). Despite these challenges, the findings from our scoping review were consistent with the keyword analysis.

Notwithstanding the aforementioned limitations, most of which are inherent to all scoping reviews and bibliometric analyses [93,96,103,104,158-160], we are confident that our findings can provide a comprehensive picture of what has been published until now (the current state of research and general directions) regarding the use of AHDs for addressing health issues in farming populations. This study may lay the groundwork for researchers to quickly identify research priorities and emerging research directions investigating health issues in farming populations using AHDs.

### Conclusions

Technological advancements have greatly increased the volume of research data available, positioning AHDs as critical resources for population-based public health studies [41]. Our review underscores the broad public health implications of AHDs, providing actionable insights for researchers, physicians, and policy makers (Textbox 3). Addressing the identified research gaps is crucial to comprehensively understanding health risks in farming populations.

The insights derived from AHDs can inform meaningful recommendations for policy makers and guide future research directions, ultimately aiding health services and health policy development. Our findings underscore the necessity of comprehensive, interdisciplinary approaches to better understand and mitigate the health risks encountered by farming populations. Such efforts will improve data comparability and research quality while also supporting the formulation of targeted prevention strategies. This, in turn, will enhance health outcomes for farming populations and promote the sustainability of agriculture in an increasingly dynamic environment. The findings from this review offer insights that are not only relevant to farming populations but also potentially generalizable to other populations.

**Take-home messages.**

**Farming population**

Research focusing on low- and middle-income countries, as well as on underrepresented subgroups within farming communities (eg, women, children, and contingent workers), remains insufficiently developed. These areas warrant further investigation to ensure more comprehensive insights.

**Administrative health database (AHD) use**

The use of AHDs in public health research among farming populations is expanding, offering major potential to enhance epidemiological studies and inform public health decisions. Promoting AHD-based research alongside the integration of other secondary data and artificial intelligence approaches represents a promising direction for future exploration. There is also a need to promote findable, accessible, interoperable, and reusable principles. Creating an AHD catalog or inventory could be a solution that would allow researchers to conduct a “datagraphic search” akin to traditional bibliographic research.

**Farming exposure**

Published studies on farming-related exposures often rely on broad proxies, such as job titles, neglecting to capture the nuances of specific agricultural tasks. While pesticide exposure remains a predominant research focus, emerging concerns, such as per- and polyfluoroalkyl substances, biological agents, micro or nanoplastics, and the effects of climate change, require urgent attention. The farming exposome remains underexplored despite its potential to uncover important associations between risk factors and a diverse range of health outcomes.

**Health outcomes**

Cancer, respiratory diseases, neurodegenerative disorders, and mental health issues are among the most frequently studied health outcomes in farming populations. However, significant gaps exist in understanding other critical conditions, such as cardiovascular diseases, reproductive disorders, ocular conditions, autoimmune diseases, musculoskeletal disorders, age-related health issues, and microbiome impacts. Addressing these overlooked areas is essential for a more complete understanding of the health risks faced by farming communities.

## Appendix

Multimedia Appendix 1 Supplementary materials.

Multimedia Appendix 2 Publications included and analyzed.

Multimedia Appendix 3 Co-occurrence between exposome-related keywords and health event-related keywords.

Multimedia Appendix 4 PRISMA-ScR checklist.

Multimedia Appendix 5 BIBLIO checklist for reporting the bibliometric reviews of the biomedical literature.

## Abbreviations

AGR: annual growth rate
AGRICAN: Agriculture and Cancer
AGRICOH: agricultural cohort
AHD: administrative health database
AHS: Agricultural Health Study
AI: artificial intelligence
AMI: Aging Multidisciplinary Investigation
ANR: French National Research Agency (Agence Nationale de la Recherche in French)
AS: association strength
BIBLIO: preliminary guideline for reporting bibliometric reviews of the biomedical literature
COPD: chronic obstructive pulmonary disease
EHR: electronic health record
EMR: electronic medical record
FAIR: findable, accessible, interoperable, and reusable
FERMA: risk factors of the rural environment and the allergic and respiratory disease
GDP: gross domestic product
JEM: job-exposure matrix
MIAI: Multidisciplinary Institute in Artificial Intelligence
MSA: Mutualité Sociale Agricole
NOCCA: Nordic Occupational Cancer Study
PRISMA-ScR: Preferred Reporting Items for Systematic Reviews and Meta-Analyses extension for Scoping Reviews
TRACTOR: Tracking and Monitoring Occupational Risks in Agriculture

## Data Availability

The datasets generated and analyzed during this study are available from the corresponding author upon reasonable request and also in the multimedia appendices.
