# Supplementary material for: Leveraging Administrative Health Databases to Address Health Challenges in Farming Populations: Scoping Review and Bibliometric Analysis (1975-2024)
Source: JMIR Public Health Surveill. 2025 Jan 9;11:e62939. doi: 10.2196/62939 (PMC11757986; doi:10.2196/62939)
Supplement: Multimedia Appendix 1 [file publichealth_v11i1e62939_app1.docx]

**Multimedia Appendices**

**Title**: Leveraging administrative health databases to address health challenges in farming populations: a scoping review and bibliometric analysis (1975-2024)

**Authors**

Pascal Petit^1*^; Nicolas Vuillerme^1,2^

^1^ Univ. Grenoble Alpes, AGEIS, 38000 Grenoble, France

^2^ Institut Universitaire de France, Paris, France

**Correspondence (present address)**

⁎ Corresponding author at: Pascal Petit. Laboratoire AGEIS – Université Grenoble Alpes. Bureau 315. Bâtiment Jean Roget. UFR de Médecine. Domaine de La Merci. 38706 La Tronche Cedex, France.

E-mail address: [pascal.petit@univ-grenoble-alpes.fr](mailto:pascal.petit@univ-grenoble-alpes.fr) (P. Petit). Telephone: + 33 4 76 63 71 04.

**Table of content**

[**Table S1**. Preferred Reporting Items for Systematic reviews and Meta-Analyses extension for Scoping Reviews (PRISMA-ScR) Checklist. 3](#_Toc184973225)

[**Table S2**. Criteria used for the publication selection. 4](#_Toc184973226)

[**Table S3**. Bibliometric index metrics. 5](#_Toc184973227)

[**Table S4**. The BIBLIO checklist for reporting the bibliometric reviews of the biomedical literature. 6](#_Toc184973228)

[**Figure S1**. Publication type. 7](#_Toc184973229)

[**Figure S2**. Scientific productivity in terms of publications per period of time. 8](#_Toc184973230)

[**Figure S3**. Historical direct citation network. 9](#_Toc184973231)

[**Figure S4**. Most prolific authors’ country. 11](#_Toc184973232)

[**Table S5**. Top 25 of the most cited publications. 12](#_Toc184973233)

[**Table S6**. Other bibliometric analysis information. 13](#_Toc184973234)

[**Multimedia appendix 2**. Publications included and analyzed. 20](#_Toc184973235)

[**Figure S5**. Number of publications per country. 21](#_Toc184973236)

[**Figure S6**. Main goal addressed by study. 22](#_Toc184973237)

[**Figure S7**. Number of publications per cohort. 23](#_Toc184973238)

[**Figure S8**. Type of digital data used. 24](#_Toc184973239)

[**Figure S9**. Active data used. 25](#_Toc184973240)

[**Figure S10**. Most frequent farming exposure proxy. 26](#_Toc184973241)

[**Figure S11**. Most frequent health events studied. 27](#_Toc184973242)

[**Figure S12**. Most frequent cancer studied. 28](#_Toc184973243)

[**Figure S13**. Most frequent neurodegenerative disease studied. 29](#_Toc184973244)

[**Figure S14**. Most frequent mental health issue studied. 30](#_Toc184973245)

[**Table S7**. Top 50 of the most frequent keywords. 31](#_Toc184973246)

[**Figure S15**. Top 5 of the most frequent keywords – growth trends. 33](#_Toc184973247)

[**Figure S16**. Top ten of the most popular keywords by total number of years of appearance. 34](#_Toc184973248)

[**Figure S17**. Top 10 of the most frequent keywords by time period. 35](#_Toc184973249)

[**Multimedia appendix 3**. Co-occurrence between exposome-related keywords and health event-related keywords. 36](#_Toc184973250)

[**Figure S18**. Keyword co-occurrence network between potential risk factor and mental health disorder keywords. 37](#_Toc184973251)

[**Figure S19**. Chord diagram of keyword co-occurrence between potential risk factor and neurodegenerative disease keywords. 38](#_Toc184973252)

[**Figure S20**. Keyword co-occurrence network between potential risk factor and neurodegenerative disease keywords. 39](#_Toc184973253)

[**Figure S21**. Chord diagram of keyword co-occurrence between potential risk factor and autoimmune disease keywords. 40](#_Toc184973254)

[**Figure S22**. Keyword co-occurrence network between potential risk factor and autoimmune disease keywords. 41](#_Toc184973255)

[**Figure S23**. Chord diagram of keyword co-occurrence between potential risk factor and breast and genital organ cancer keywords. 42](#_Toc184973256)

[**Figure S24**. Keyword co-occurrence network between potential risk factor and breast and genital organ cancer keywords. 43](#_Toc184973257)

[**Figure S25**. Chord diagram of keyword co-occurrence between potential risk factor and digestive organ, lip, oral cavity and pharynx cancer keywords. 44](#_Toc184973258)

[**Figure S26**. Keyword co-occurrence network between potential risk factor and digestive organ, lip, oral cavity and pharynx cancer keywords. 45](#_Toc184973259)

[**Figure S27**. Chord diagram of keyword co-occurrence between potential risk factor and lymphohematopoietic and mesenchymal cancer keywords. 46](#_Toc184973260)

[**Figure S28**. Keyword co-occurrence network between potential risk factor and lymphohematopoietic and mesenchymal cancer keywords. 47](#_Toc184973261)

[**Figure S29**. Chord diagram of keyword co-occurrence between potential risk factor and brain, skin, ocular and endocrine gland cancer keywords. 48](#_Toc184973262)

[**Figure S30**. Keyword co-occurrence network between potential risk factor and brain, skin, ocular and endocrine gland cancer keywords. 49](#_Toc184973263)

[**Figure S31**. Chord diagram of keyword co-occurrence between potential risk factor and respiratory and urinary tract cancer keywords. 50](#_Toc184973264)

[**Figure S32**. Keyword co-occurrence network between potential risk factor and respiratory and urinary tract cancer keywords. 51](#_Toc184973265)

[**Figure S33**. Chord diagram of keyword co-occurrence between potential risk factor and infectious disease keywords. 52](#_Toc184973266)

[**Figure S34**. Keyword co-occurrence network between potential risk factor and infectious disease keywords. 53](#_Toc184973267)

[**Figure S35**. Chord diagram of keyword co-occurrence between potential risk factor and respiratory disorder keywords. 54](#_Toc184973268)

[**Figure S36**. Keyword co-occurrence network between potential risk factor and respiratory disorder keywords. 55](#_Toc184973269)

[**Figure S37**. Chord diagram of keyword co-occurrence between potential risk factor and reproductive disorder keywords. 56](#_Toc184973270)

[**Figure S38**. Keyword co-occurrence network between potential risk factor and reproductive disorder keywords. 57](#_Toc184973271)

[**Figure S39**. Chord diagram of keyword co-occurrence between potential risk factor and injury and work-related disease keywords. 58](#_Toc184973272)

[**Figure S40**. Keyword co-occurrence network between potential risk factor and injury and work-related disease keywords. 59](#_Toc184973273)

[**Figure S41**. Chord diagram of keyword co-occurrence between potential risk factor and other health event keywords. 60](#_Toc184973274)

[**Figure S42**. Keyword co-occurrence network between potential risk factor and other health event keywords. 61](#_Toc184973275)

[**Figure S43**. Keyword co-occurrence network between potential risk factor and mortality keywords. 62](#_Toc184973276)

[**Abbreviations** 63](#_Toc184973277)

[**References** 64](#_Toc184973278)

# **Table S1**. Preferred Reporting Items for Systematic reviews and Meta-Analyses extension for Scoping Reviews (PRISMA-ScR) Checklist.

| **SECTION** | **ITEM** | **PRISMA-ScR CHECKLIST ITEM** | **REPORTED ON PAGE #** |
| --- | --- | --- | --- |
| **TITLE** | | | |
| Title | 1 | Identify the report as a scoping review. | 1 |
| **ABSTRACT** | | | |
| Structured summary | 2 | Provide a structured summary that includes (as applicable): background, objectives, eligibility criteria, sources of evidence, charting methods, results, and conclusions that relate to the review questions and objectives. | 2, 3 |
| **INTRODUCTION** | | | |
| Rationale | 3 | Describe the rationale for the review in the context of what is already known. Explain why the review questions/objectives lend themselves to a scoping review approach. | 4-7 |
| Objectives | 4 | Provide an explicit statement of the questions and objectives being addressed with reference to their key elements (e.g., population or participants, concepts, and context) or other relevant key elements used to conceptualize the review questions and/or objectives. | 7 |
| **METHODS** | | | |
| Protocol and registration | 5 | Indicate whether a review protocol exists; state if and where it can be accessed (e.g., a Web address); and if available, provide registration information, including the registration number. | 8 |
| Eligibility criteria | 6 | Specify characteristics of the sources of evidence used as eligibility criteria (e.g., years considered, language, and publication status), and provide a rationale. | 8-10 |
| Information sources* | 7 | Describe all information sources in the search (e.g., databases with dates of coverage and contact with authors to identify additional sources), as well as the date the most recent search was executed. | 8-10 |
| Search | 8 | Present the full electronic search strategy for at least 1 database, including any limits used, such that it could be repeated. | 9 |
| Selection of sources of evidence† | 9 | State the process for selecting sources of evidence (i.e., screening and eligibility) included in the scoping review. | 8-10 |
| Data charting process‡ | 10 | Describe the methods of charting data from the included sources of evidence (e.g., calibrated forms or forms that have been tested by the team before their use, and whether data charting was done independently or in duplicate) and any processes for obtaining and confirming data from investigators. | 10-14 |
| Data items | 11 | List and define all variables for which data were sought and any assumptions and simplifications made. | 10-14 |
| Critical appraisal of individual sources of evidence§ | 12 | If done, provide a rationale for conducting a critical appraisal of included sources of evidence; describe the methods used and how this information was used in any data synthesis (if appropriate). | NA |
| Synthesis of results | 13 | Describe the methods of handling and summarizing the data that were charted. | 10-14 |
| **RESULTS** | | | |
| Selection of sources of evidence | 14 | Give numbers of sources of evidence screened, assessed for eligibility, and included in the review, with reasons for exclusions at each stage, ideally using a flow diagram. | 14-17, supplementary materials |
| Characteristics of sources of evidence | 15 | For each source of evidence, present characteristics for which data were charted and provide the citations. | 14-26, supplementary materials |
| Critical appraisal within sources of evidence | 16 | If done, present data on critical appraisal of included sources of evidence (see item 12). | NA |
| Results of individual sources of evidence | 17 | For each included source of evidence, present the relevant data that were charted that relate to the review questions and objectives. | 14-26, supplementary materials |
| Synthesis of results | 18 | Summarize and/or present the charting results as they relate to the review questions and objectives. | 14-39, supplementary materials |
| **DISCUSSION** | | | |
| Summary of evidence | 19 | Summarize the main results (including an overview of concepts, themes, and types of evidence available), link to the review questions and objectives, and consider the relevance to key groups. | 40-47 |
| Limitations | 20 | Discuss the limitations of the scoping review process. | 48, 49 |
| Conclusions | 21 | Provide a general interpretation of the results with respect to the review questions and objectives, as well as potential implications and/or next steps. | 49-51 |
| **FUNDING** | | | |
| Funding | 22 | Describe sources of funding for the included sources of evidence, as well as sources of funding for the scoping review. Describe the role of the funders of the scoping review. | 52 |

JBI = Joanna Briggs Institute; PRISMA-ScR = Preferred Reporting Items for Systematic reviews and Meta-Analyses extension for Scoping Reviews.

* Where *sources of evidence* (see second footnote) are compiled from, such as bibliographic databases, social media platforms, and Web sites.

† A more inclusive/heterogeneous term used to account for the different types of evidence or data sources (e.g., quantitative and/or qualitative research, expert opinion, and policy documents) that may be eligible in a scoping review as opposed to only studies. This is not to be confused with *information sources* (see first footnote).

‡ The frameworks by Arksey and O’Malley (6) and Levac and colleagues (7) and the JBI guidance (4, 5) refer to the process of data extraction in a scoping review as data charting*.*

§ The process of systematically examining research evidence to assess its validity, results, and relevance before using it to inform a decision. This term is used for items 12 and 19 instead of "risk of bias" (which is more applicable to systematic reviews of interventions) to include and acknowledge the various sources of evidence that may be used in a scoping review (e.g., quantitative and/or qualitative research, expert opinion, and policy document).

Peters MDJ, Godfrey C, McInerney P, BaldiniSoares C, Khalil H, Parker D. Scoping reviews. In: Aromataris E, Munn Z, eds. Joanna Briggs Institute Reviewer's Manual. Adelaide, Australia: Joanna Briggs Inst; 2017.

Arksey H, O'Malley L. Scoping studies: towards a methodological framework. Int J Soc Res Methodol. 2005;8:19-32. doi: 10.1080/1364557032000119616

Peters MD, Godfrey CM, Khalil H, McInerney P, Parker D, Soares CB. Guidance for conducting systematic scoping reviews. Int J Evid Based Healthc. 2015;13(3):141-146. doi:10.1097/XEB.0000000000000050

Levac D, Colquhoun H, O'Brien KK. Scoping studies: advancing the methodology. Implement Sci. 2010;5:69. Published 2010 Sep 20. doi:10.1186/1748-5908-5-69.

*From:* Tricco AC, Lillie E, Zarin W, O'Brien KK, Colquhoun H, Levac D, et al. PRISMA Extension for Scoping Reviews (PRISMAScR): Checklist and Explanation. Ann Intern Med. 2018;169:467–473. [doi: 10.7326/M18-0850](http://annals.org/aim/fullarticle/2700389/prisma-extension-scoping-reviews-prisma-scr-checklist-explanation).

# **Table S2**. Criteria used for the publication selection.

| **Question** | **Description** | **Answer** | |
| --- | --- | --- | --- |
|  |  | no | yes/can’t tell |
| **Stage 1: Record language** | | | |
| *Q_11_* | Is the record written in English or French? | 0 | 1 |
| *S_1_ = Q_11_; Record eligible for stage 2 if S_1_ > 0* | | | |
| **Stage 2: Screening record title** | | | |
| *Q_21_* | Does the title mention terms related to administrative health data? | 0 | 1 |
| *Q_22_* | Does the title mention terms related to humans? | 0 | 1 |
| *Q_23_* | Does the title mention terms related to farming? | 0 | 1 |
| *Q_24_* | Does the title mention terms related to public health or epidemiological research? | 0 | 1 |
| *S_2_ = Q_21_ × Q_22_× Q_23_× Q_24_; Record eligible for stage 3 if S_2_ = 1* | | | |
| **Stage 3: Screening record abstract** | | | |
| *Q_31_* | Does the abstract describe an analysis using administrative health database? | 0 | 1 |
| *Q_32_* | Does the abstract describe an analysis conducted/related to humans? | 0 | 1 |
| *Q_33_* | Does the abstract describe an analysis related to farming? | 0 | 1 |
| *Q_34_* | Does the abstract describe an analysis related public health or epidemiological research? | 0 | 1 |
| *S_3_ = Q_31_ × Q_32_× Q_33_× Q_34_; Record eligible for stage 4 if S_3_ = 1* | | | |
| **Stage 4: Screening record content** | | no | yes |
| *Q_41_* | Does the record describe an analysis using administrative health database? | 0 | 1 |
| *Q_42_* | Does the record describe an analysis conducted/related to humans? | 0 | 1 |
| *Q_43_* | Does the record describe an analysis conducted/related to farming population? | 0 | 1 |
| *Q_44_* | Does the record describe an analysis related public health or epidemiological research? | 0 | 1 |
| *S_4_ = Q_41_ × Q_42_× Q_43_× Q_44_; Record eligible for stage 4 if S_4_ = 1* | | | |
| ***Score = S_1_ × S_2_ × S_3_× S_4_; Record included and analyzed if score ≥ 1*** | | | |

*Note*: Q: question, S: score.

# **Table S3**. Bibliometric index metrics.

- **h index**

The h index attempts to measure both the productivity and citation impact of the published body of work of an entity (e.g., author, institution, journal) [1,2]. It refers to the total number of publications by a particular entity with at least the same number of citations.

- **m index**

The m index is calculated by dividing the h index by the number of years of an entity’s productive life (e.g., researcher) [1].

- **g index**

The g index of an entity corresponds to the largest number g such that the top g publications have at least g^2^ or more citations together [2].

- **Y index**

The Y index refers to the sum of both the total number of first-authored publications and the total number of corresponding-author publications [3].

- **Dominance factor**

The dominance factor (DF) refers, for a particular researcher, to the proportion of multi-authored publications as specific author’s rank to the total number of multi-authored publications [4].

The dominance factor for being a first/primary author (DF_first_) refers to the proportion of multi-authored publications as a first author to the total number of multi-authored publications. It is calculated as follows:

$${DF}_{first}=\frac{number of multi-authored papers as first author}{total number of multi-authored papers}\times100$$

The dominance factor for being a last/senior author (DF_last_) refers to the proportion of multi-authored publications as a last author to the total number of multi-authored publications. It is calculated as follows:

$${DF}_{last}=\frac{number of multi-authored papers as last author}{total number of multi-authored papers}\times100$$

- **Fractionalized frequency**

The fractional frequency (FF) intends to reflect/measure an author’s contribution. For one publication, it is calculated as follows:

${FF}_{i}=\frac{1}{total number of authors in the {paper}_{i}}$

For a given researcher, the FF corresponds to:

${FF}_{author}=\sum_{i=1}^{n} {FF}_{i}$, where n refers to the total number of publications for which a researcher was an author.

- **Annual growth rate**

The annual growth rate refers to the variable’s change in percentage as a year-over-year statistic [5], and is calculated as follows:

$$Annual Growth Rate=\frac{\left( end value-first value \right)}{first value}\times100$$

# **Table S4**. The BIBLIO checklist for reporting the bibliometric reviews of the biomedical literature.

| **Section/Topic** | **Item No.** | **Checklist item** | **Reported on**  **page No.** |  |
| --- | --- | --- | --- | --- |
| **Title** |  |  |  |  |
| Identification | 1 | Identify the report as a bibliometric review in the title. | 1 |  |
| Issues/topics | 2 | Indicate the key issues/topics under investigation and coverage of time period. | 1 |  |
| **Abstract** |  |  |  |  |
| Structured summary | 3 | Structured summary including (as applicable): background, methods, results (key findings) and conclusions. | 2, 3 |  |
| **Introduction/ Background** |  |  |  |  |
| Justification/ Rationale/ Explanation | 4 | Present review of existing knowledge and epidemiological information. | 4-7 |  |
| Objectives | 5 | Statement of the objective (s) or question (s). | 7 |  |
| **Methods** |  |  |  |  |
| Search engines (data sources) | 6 | Describe all information sources (such as electronic databases, contact with study authors, trial registers or other grey literature sources). | 8-10 |  |
| Search strategy | 7 | Keywords and systematization criteria (date of search, language, type of document) for the search. | 8-11 |  |
| Time period | 8 | The period that the review covers and the justification. | 9 |  |
| Eligibility criteria | 9 | Describe all inclusion and exclusion criteria; languages; study design, type of publication and time period. | 9, 10 |  |
| Data refinement (data selection procedure) | 10 | Remove the irrelevant articles; inspection to eliminate duplicate and unrelated articles (after evaluation of the title, abstract and content). | 9, 10 |  |
| Quality assessment (optional) | 11 | Assessment of papers by three authors and the use of assessing checklists. | NA |  |
| Data synthesis | 12 | Describe the methods used for summarizing, handling, synthesis, tabulations or schematic displays. Describe how the data were analyzed. | 10-14 |  |
| **Results** |  |  |  |  |
| Descriptive findings (statistics) | 13 | - Provide details of the search and selection process in a flow diagram.  - Number of citations retrieved (number of publication, year of publication, type of documents, country of publication, articles with the highest impact, most impactful authors, most impactful articles, authors with the highest production, top journals, top institutions, …) | 14-17, supplementary materials |  |
| Schematic map and trend | 14 | Summarize and/or present the schematic maps and trends using an appropriate software to present citations, journals, authors, top journals, time trends, emerging literature, and any relevant indicators (as applicable) [1-5]. | 27-39, supplementary materials |  |
| Tabulation and summarizing the findings | 15 | General recommendation: Studies under consideration could be summarized and organized by different subtitles and different scenarios. Regardless, results need to be presented in separate tables covering each subtitle. The followings are some options that could help to summarize the findings.  *Option 1:*  - Start the presentation with a historical view [when and who first published on the topic].  - Report on review papers. The result should be listed in a separate table. Also, specify the review type (scoping review, narrative review, systematic review, and meta-analysis).  - Summarize the findings according to the study designs and main study types.  *Option 2:*  - Start the presentation with a historical view [when and who first published on the topic].  - Report on review papers. The result should be listed in a separate table. Also, indicate the review type (scoping review, narrative review, systematic review, and meta-analysis) should be specified.  - Summarize the findings according to outcome measures or populations. For example, see [6].  *Option 3:*  - Start the presentation with a historical view [when and who first published on the topic].  - Report on review papers. The result should be listed in a separate table. Also, specify the review type (scoping review, narrative review, systematic review, and meta-analysis).  - Summarize the findings according to concept [7].  *Option 4.*  - Start the presentation with a historical view [when and who first published on the topic].  - Report on review papers. The result should be listed in a separate table, and also specify the review type (scoping review, narrative review, systematic review, and meta-analysis).  - Summarize the findings according to different subtitles relevant to the main topic [8]. | 14-39, supplementary materials |  |
| Synthesis of findings | 16 | Synthesize the findings as much as possible, find the gap, and propose a model, hypothesis, etc. (if applicable). | 27-39, supplementary materials |  |
| **Discussion** |  |  |  |  |
| Summary of evidence | 17 | Summarize the main findings. The findings should be presented in more "general" or "accessible" terms. | 40-48 |  |
| Interpretation | 18 | Include interpretation consistent with results. Explanations for observed outcomes, similarities, and differences reported would be essential. | 40-48 |  |
| Strengths and limitations | 19 | Discuss the strengths and limitations. | 48-49 |  |
| Conclusion(s) | 20 | Provide a general interpretation of the results with respect to the review questions and objectives, as well as potential implications. | 49-51 |  |
| 1. McDougal L, Dehingia N, Cheung WW, Dixit A, Raj A. COVID-19 burden, author affiliation and women's well-being: A bibliometric analysis of COVID-19 related publications including focus on low-and middle-income countries. eClinicalMedicine 2022; 52: 101606.  2. Henstock L, Wong R, Tsuchiya A, Spencer A. Behavioral theories that have influenced the way health state preferences are elicited and interpreted: A bibliometric mapping analysis of the ttime trade-off method with VOSviewer visualization. Front Health Serv 2022; 2: 848087.  3. Bodea F, Bungau SG, Negru AP, Radu A, Tarce AG, Tit DM, et al. Exploring new therapeutic avenues for ophthalmic disorders: Glaucoma-related molecular docking evaluation and bibliometric analysis for improved management of ocular diseases. Bioengineering 2023; 10(8): 983.  4. Sang XZ, Wang CQ, Chen W, Rong H, Hou LJ. An exhaustive analysis of post-traumatic brain injury dementia using bibliometric methodologies. Front Neurol 2023; 14: 1165059.  5. Ramli MI, Hamzaid NA, Engkasan JP, Usman J. Respiratory muscle training: a bibliometric analysis of 60 years’ multidisciplinary journey. Biomed Eng Online 2023; 22(1): 50.  6. Akosman I, Kumar N, Mortenson R, Lans A, De La Garza Ramos R, Eleswarapu A,et al. Racial differences in perioperative complications, readmissions, and mortalities after elective spine surgery in the United States: A systematic review using AI-assisted bibliometric analysis. Glob Spine J 2023: 21925682231186759.  7. Tavousi M, Mohammadi S, Sadighi J, Zarei F, Kermani RM, Rostami R, Montazeri A. Measuring health literacy: A systematic review and bibliometric analysis of instruments from 1993 to 2021. Plos One 2022; 17(7): e0271524.  8. Montazeri A. Health-related quality of life in breast cancer patients: A bibliographic review of the literature from 1974 to 2007. J Exp Clin Cancer Res 2008; 27: 32. | | | | |

**Rights and permissions:** The original source of the checklist is: Montazeri A, Mohammadi S, M.Hesari P, Ghaemi M, Riazi H, Sheikhi‑Mobarakeh Z. Preliminary guideline for reporting bibliometric reviews of the biomedical literature (BIBLIO): a minimum requirements. *Systematic Reviews* 2023; 12: 239. doi: 10.1186/s13643-023-02410-2. The article is licensed under a Creative Commons Attribution 4.0 International License (<http://creativecommons.org/licenses/by/4.0/>). A changes was made to the original checklist to add in full references to the cited sources.

# **Figure S1**. Publication type.


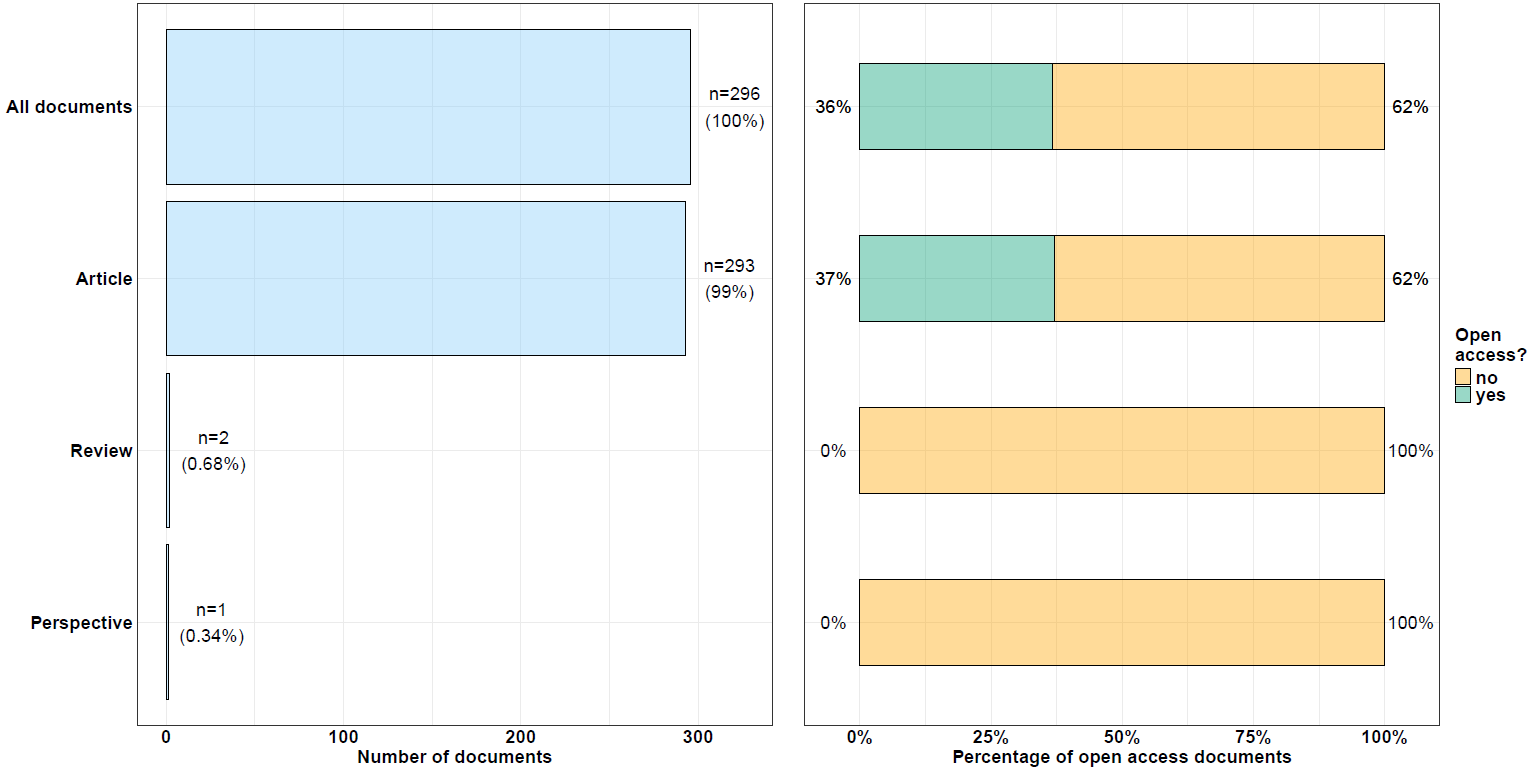


# **Figure S2**. Scientific productivity in terms of publications per period of time.


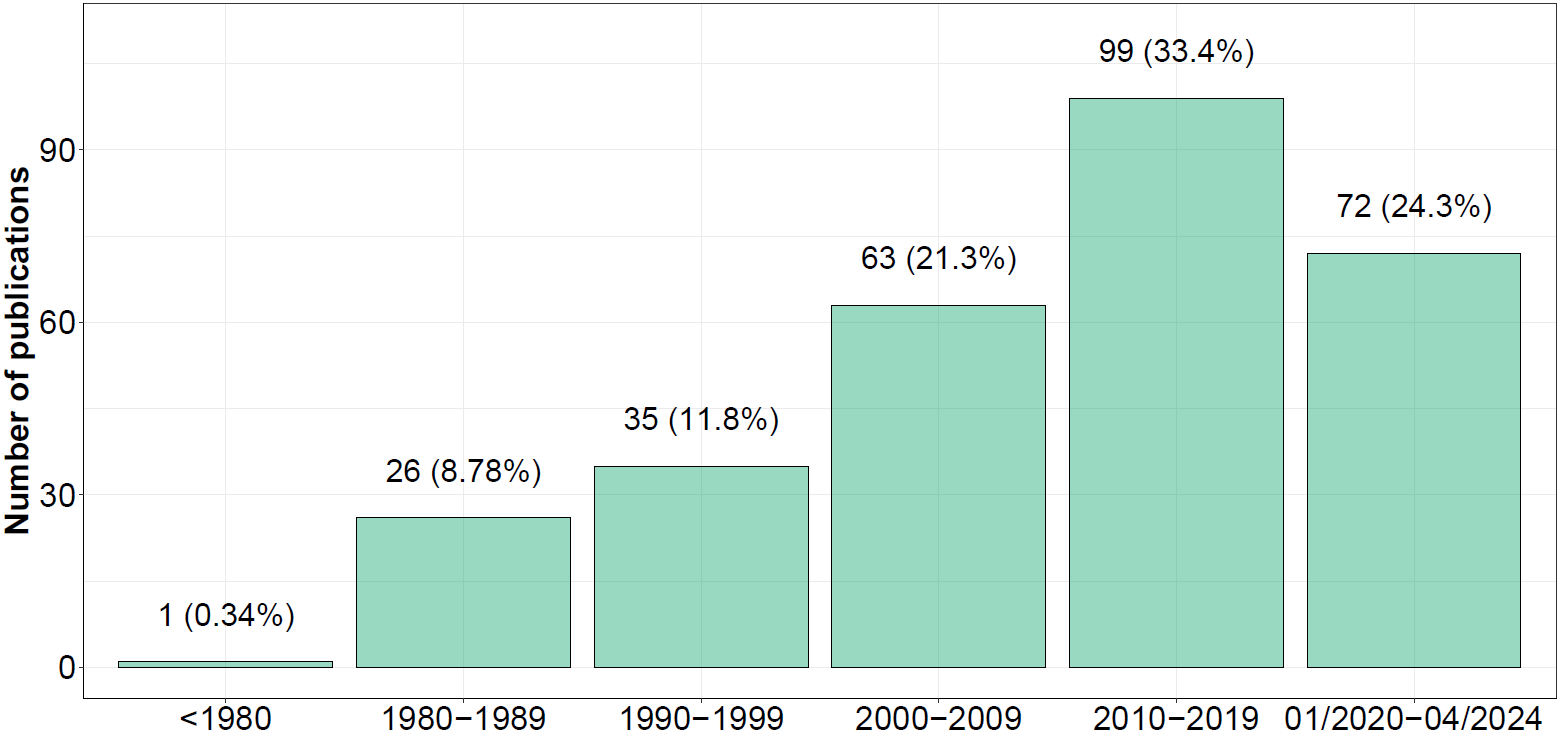


# **Figure S3**. Historical direct citation network.


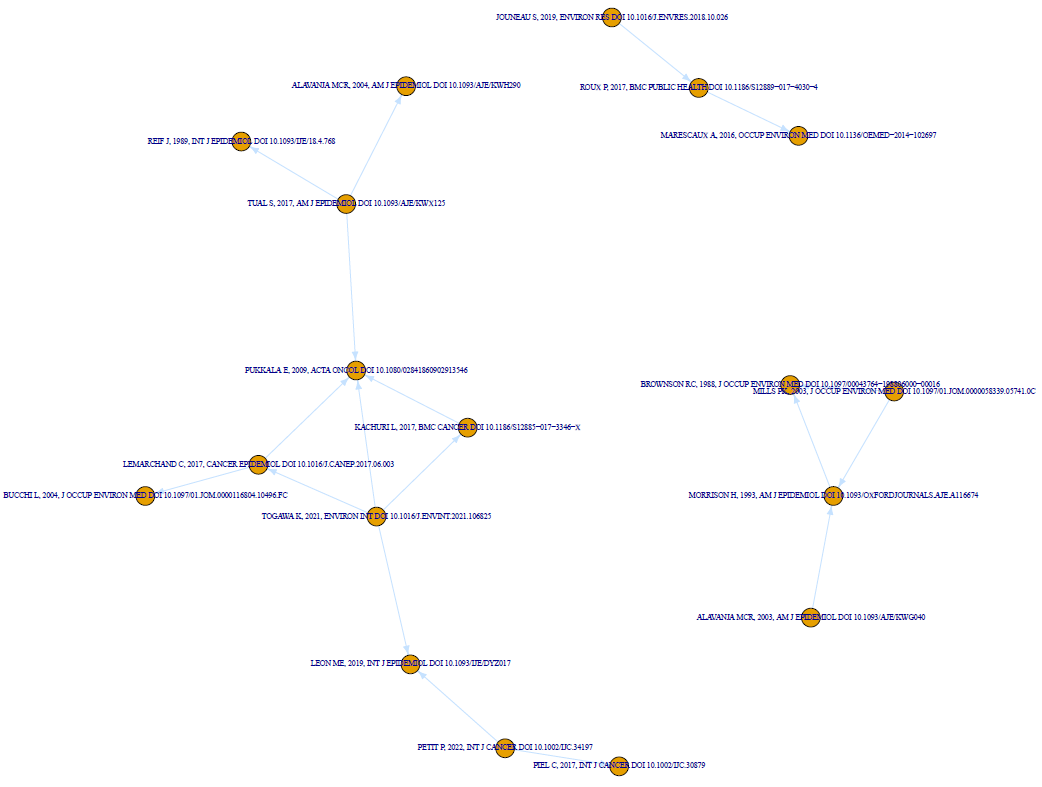


Jouneau S, Marette S, Robert AM, et al. Prevalence and risk factors of chronic obstructive pulmonary disease in dairy farmers: AIRBAg study. Environ Res. 2019;169:1-6. doi:10.1016/j.envres.2018.10.026

Roux P, Guillien A, Soumagne T, et al. Smoking habits in French farmers: a cross-sectional study. BMC Public Health. 2017;17(1):166. doi:10.1186/s12889-017-4030-4

Alavanja MC, Dosemeci M, Samanic C, et al. Pesticides and lung cancer risk in the agricultural health study cohort. Am J Epidemiol. 2004;160(9):876-885. doi:10.1093/aje/kwh290

Reif J, Pearce N, Fraser J. Cancer risks in New Zealand farmers. Int J Epidemiol. 1989;18(4):768-774. doi:10.1093/ije/18.4.768

Marescaux A, Degano B, Soumagne T, Thaon I, Laplante JJ, Dalphin JC. Impact of farm modernity on the prevalence of chronic obstructive pulmonary disease in dairy farmers. Occup Environ Med. 2016;73(2):127-133. doi:10.1136/oemed-2014-102697

Tual S, Lemarchand C, Boulanger M, et al. Exposure to Farm Animals and Risk of Lung Cancer in the AGRICAN Cohort. Am J Epidemiol. 2017;186(4):463-472. doi:10.1093/aje/kwx125.

Petit P, Gandon G, Chabardès S, Bonneterre V. Agricultural activities and risk of central nervous system tumors among French farm managers: Results from the TRACTOR project. Int J Cancer. 2022;151(10):1737-1749. doi:10.1002/ijc.34197.

Piel C, Pouchieu C, Tual S, et al. Central nervous system tumors and agricultural exposures in the prospective cohort AGRICAN. Int J Cancer. 2017;141(9):1771-1782. doi:10.1002/ijc.30879

Lemarchand C, Tual S, Levêque-Morlais N, et al. Cancer incidence in the AGRICAN cohort study (2005-2011). Cancer Epidemiol. 2017;49:175-185. doi:10.1016/j.canep.2017.06.003.

Morrison H, Savitz D, Semenciw R, et al. Farming and prostate cancer mortality. Am J Epidemiol. 1993;137(3):270-280. doi:10.1093/oxfordjournals.aje.a116674.

Alavanja MC, Samanic C, Dosemeci M, et al. Use of agricultural pesticides and prostate cancer risk in the Agricultural Health Study cohort. Am J Epidemiol. 2003;157(9):800-814. doi:10.1093/aje/kwg040

Mills PK, Yang R. Prostate cancer risk in California farm workers. J Occup Environ Med. 2003;45(3):249-258. doi:10.1097/01.jom.0000058339.05741.0c.

Kachuri L, Harris MA, MacLeod JS, Tjepkema M, Peters PA, Demers PA. Cancer risks in a population-based study of 70,570 agricultural workers: results from the Canadian census health and Environment cohort (CanCHEC). BMC Cancer. 2017;17(1):343. doi:10.1186/s12885-017-3346-x

Togawa K, Leon ME, Lebailly P, et al. Cancer incidence in agricultural workers: Findings from an international consortium of agricultural cohort studies (AGRICOH). Environ Int. 2021;157:106825. doi:10.1016/j.envint.2021.106825.

Pukkala E, Martinsen JI, Lynge E, et al. Occupation and cancer - follow-up of 15 million people in five Nordic countries. Acta Oncol. 2009;48(5):646-790. doi:10.1080/02841860902913546

Leon ME, Schinasi LH, Lebailly P, et al. Pesticide use and risk of non-Hodgkin lymphoid malignancies in agricultural cohorts from France, Norway and the USA: a pooled analysis from the AGRICOH consortium. Int J Epidemiol. 2019;48(5):1519-1535. doi:10.1093/ije/dyz017

Bucchi L, Nanni O, Ravaioli A, et al. Cancer mortality in a cohort of male agricultural workers from northern Italy. J Occup Environ Med. 2004;46(3):249-256. doi:10.1097/01.jom.0000116804.10496.fc

Brownson RC, Chang JC, Davis JR, Bagby JR Jr. Occupational risk of prostate cancer: a cancer registry-based study. J Occup Med. 1988;30(6):523-526.

# **Figure S4**. Most prolific authors’ country.

MPC: multi-country publication, SCP: single-country publication.


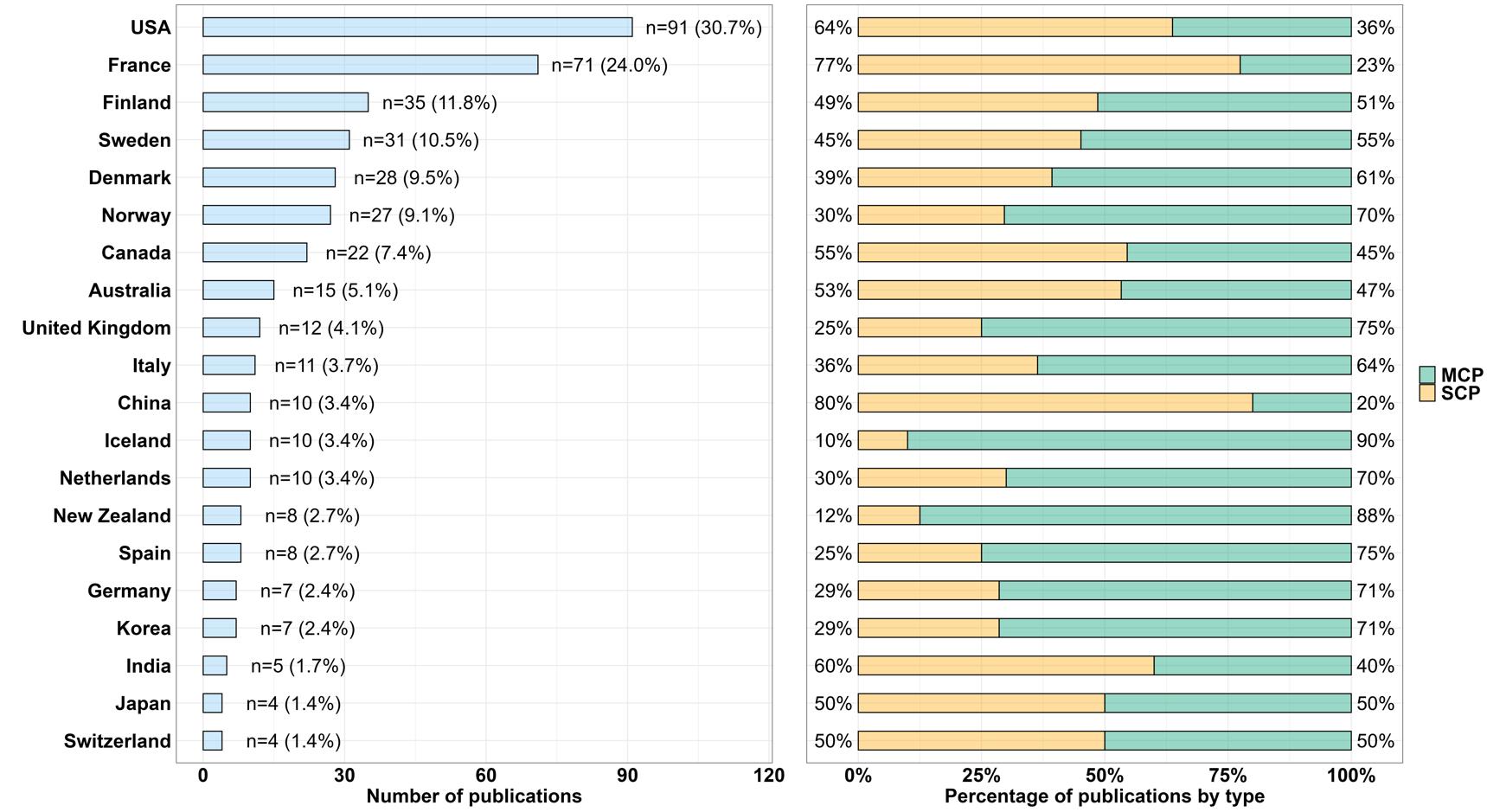


# **Table S5**. Top 25 of the most cited publications.

| **Publication** | **Publication**  **year** | **Journal** | **DOI** | **Total**  **citation** | **Local**  **citation**  **(%)** | **Average**  **citation**  **per year** |
| --- | --- | --- | --- | --- | --- | --- |
| Pukkala et al. 2009 [6] | 2009 | Acta Oncol | 10.1080/02841860902913546 | 485 | 21 (4.33%) | 30.3 |
| Alavanja et al. 2003 [7] | 2003 | Am J Epidemiol | 10.1093/aje/kwg040 | 283 | 6 (2.12%) | 12.9 |
| Elbaz et al. 2009 [8] | 2009 | Ann Neurol | 10.1002/ana.21717 | 206 | 7 (3.40%) | 12.9 |
| Andersen et al. 1999 [9] | 1999 | Scand J Work Envion Health | none | 206 | 0 (0.00%) | 7.92 |
| Karjalainen et al. 2001 [10] | 2001 | Am J Respir Crit Care Med | 10.1164/ajrccm.164.4.2012146 | 162 | 0 (0.00%) | 6.75 |
| Vingård et al. 1991 [11] | 1991 | Int J Epidemiol | 10.1093/ije/20.4.1025 | 157 | 1 (0.64%) | 4.62 |
| Alavanja et al. 2004 [12] | 2004 | Am J Epidemiol | 10.1093/aje/kwh290 | 155 | 7 (4.52%) | 7.38 |
| Andreotti et al. 2018 [13] | 2018 | J Natl Cancer Inst | 10.1093/jnci/djx233 | 143 | 0 (0.00%) | 20.4 |
| Wigle et al. 1990 [14] | 1990 | J Natl Cancer Inst | 10.1093/jnci/82.7.575 | 136 | 0 (0.00%) | 3.89 |
| Engel et al. 2005 [15] | 2005 | Am J Epidemiol | 10.1093/aje/kwi022 | 116 | 2 (1.72%) | 5.8 |
| Hemminki et al. 1981 [16] | 1981 | J Epidemiol Community Health | 10.1136/jech.35.1.11 | 114 | 0 (0.00%) | 2.59 |
| Weichenthal et al. 2014 [17] | 2014 | Environ Health Perspect | 10.1289/ehp.1307277 | 113 | 0 (0.00%) | 10.3 |
| Levecque et al. 2003 [18] | 2003 | Hum Mol Genet | 10.1093/hmg/ddg009 | 111 | 0 (0.00%) | 5.05 |
| Morrison et al. 1993 [19] | 1993 | Am J Epidemiol | 10.1093/oxfordjournals.aje.a116674 | 106 | 5 (4.72%) | 3.31 |
| Kristensen et al. 1996 [20] | 1996 | Scand J Work Envion Health | 10.5271/sjweh.104 | 98 | 0 (0.00%) | 3.38 |
| Orton et al. 2011 [21] | 2011 | Neurology | 10.1212/WNL.0b013e31820a0a9f | 94 | 1 (1.06%) | 6.71 |
| Stokes et al. 1995 [22] | 1995 | Occup Environ Med | 10.1136/oem.52.10.648 | 92 | 0 (0.00%) | 3.07 |
| Vukusic et al. 2007 [23] | 2007 | J Neurol Neurosurg Psychiatry | 10.1136/jnnp.2006.101196 | 90 | 0 (0.00%) | 5.00 |
| Mills and Yang, 2003 [24] | 2003 | J Occup Environ Med | 10.1097/01.jom.0000058339.05741.0c | 88 | 5 (5.68%) | 4.00 |
| Eriksson and Karlsson, 1992 [25] | 1992 | Br J Ind Med | none | 87 | 0 (0.00%) | 2.64 |
| Wiklund et al. 1989 [26] | 1989 | Br J Ind Med | none | 87 | 0 (0.00%) | 2.42 |
| Pouchieu et al. 2018 [27] | 2018 | Int J Epidemiol | 10.1093/ije/dyx225 | 86 | 1 (1.16%) | 12.3 |
| Karjalainen et al. 2003 [28] | 2003 | Chest | 10.1378/chest.123.1.283 | 85 | 0 (0.00%) | 3.86 |
| Leon et al. 2019 [29] | 2019 | Int J Epidemiol | 10.1093/ije/dyz017 | 85 | 5 (5.88%) | 14.2 |
| Lee et al. 2007 [30] | 2007 | Int J Cancer | 10.1002/ijc.22635 | 83 | 2 (2.41%) | 4.61 |
| Reif et al. 1989 [31] | 1989 | Int J Epidemiol | 10.1093/ije/18.4.768 | 83 | 4 (4.82%) | 2.31 |

*Note*: DOI: digital object identifier. Please refer to the beginning of the supplemental materials for the definition of the bibliometric indices.

# **Table S6**. Other bibliometric analysis information.

- *The most prolific countries*

Studies were led by authors from 34 countries, predominantly high-income nations, with 24.3% of studies (72/296) involving multi-country collaborations. US-based authors contributed the most publications (91/296, 30.7%), followed by France (71/296, 24.0%) and Finland (35/296, 11.8%). US authors also had the most citations (3495/9379, 37.2%), with France and Finland ranking second and third. The highest average AGRs were seen in authors from India (25%), France (15%), and the US (10.1%) (Table MA1).

Positive associations were found between the number of publications and GDP (ρ=0.46, p=5.9e-3), R&D researchers (ρ=0.62, p=1.1e-4), and cereal yield (ρ=0.41, p=0.02). In contrast, negative associations were observed with agricultural employment (ρ=-0.60, p=1.6e-4), sex-specific employment in agriculture, agricultural value added to GDP (ρ=-0.51, p=2.1e-3), as well as fertilizer consumption (ρ=-0.37, p=0.04) (Table MA2).

Countries with the highest scientific production per GDP were Iceland, Finland, and Malawi, while Iceland, Finland, and Norway led per capita and cereal production (Table MA3). The US, Norway, and Canada exhibited the highest scientific production per fertilizer consumption, and Norway, Finland, and Denmark excelled in scientific output per hectare of agricultural land. The US, France, and Finland were leaders in scientific output related to crop, food, livestock production indices, and cereal yield. Notably, the US, Sweden, and France led in output per agricultural employment, while the US, Canada, and the UK had the highest scientific production relative to agriculture, forestry, and fishing value added to GDP.

**Table MA1**. The most productive author’s countries.

| **Country** | **Continent** | **Publication period** | **Number of publications (%)** | **Total citation** | **Local citation (%)** | **Average citation per year** | **Mean AGR** | **Fractionalized frequency (%)** | **h-index** | **g-index** | **m-index** |
| --- | --- | --- | --- | --- | --- | --- | --- | --- | --- | --- | --- |
| USA | North America | 1981-2024 | 91 (30.7%) | 3495 | 81 (2.32%) | 81.3 | 10.1 | 72.6 | 37 | 57 | 0.841 |
| France | Europe | 1998-2024 | 71 (24.0%) | 1762 | 69 (3.92%) | 65.3 | 15.0 | 60.3 | 22 | 40 | 0.815 |
| Finland | Europe | 1980-2024 | 35 (11.8%) | 1429 | 30 (2.10%) | 37.2 | -16.7 | 21.6 | 17 | 35 | 0.378 |
| Sweden | Europe | 1986-2024 | 31 (10.5%) | 1290 | 33 (2.56%) | 43.9 | -17.5 | 18.2 | 19 | 31 | 0.487 |
| Denmark | Europe | 1988-2024 | 28 (9.46%) | 828 | 31 (3.74%) | 31.9 | -9.46 | 15.1 | 15 | 28 | 0.405 |
| Norway | Europe | 1996-2024 | 27 (9.12%) | 965 | 37 (3.83%) | 41.8 | -9.20 | 12.0 | 13 | 27 | 0.448 |
| Canada | North America | 1975-2022 | 22 (7.43%) | 799 | 13 (1.63%) | 18.0 | -13.9 | 16.3 | 13 | 22 | 0.260 |
| Australia | Oceania | 1986-2024 | 15 (5.07%) | 177 | 9 (5.08%) | 4.85 | -6.41 | 10.6 | 7 | 13 | 0.179 |
| UK | Europe | 1984-2021 | 12 (4.05%) | 294 | 9 (3.06%) | 8.50 | -15.8 | 6.53 | 9 | 12 | 0.220 |
| Italy | Europe | 1988-2024 | 11 (3.72%) | 170 | 7 (4.12%) | 4.59 | -9.01 | 6.58 | 7 | 11 | 0.189 |
| China | Asia | 1995-2024 | 10 (3.38%) | 85 | 0 (0.00%) | 3.73 | -16.4 | 9.00 | 4 | 10 | 0.114 |
| Iceland | Europe | 2006-2024 | 10 (3.38%) | 570 | 24 (4.21%) | 30.0 | -21.1 | 2.74 | 7 | 10 | 0.368 |
| Netherlands | Europe | 2004-2023 | 10 (3.38%) | 217 | 14 (6.45%) | 10.9 | -18.3 | 4.64 | 6 | 10 | 0.286 |
| New Zealand | Oceania | 1986-2021 | 8 (2.70%) | 364 | 11 (3.02%) | 10.1 | -5.56 | 3.86 | 7 | 8 | 0.179 |
| Spain | Europe | 1998-2023 | 8 (2.70%) | 186 | 4 (2.15%) | 7.31 | -23.1 | 4.32 | 6 | 8 | 0.222 |
| Germany | Europe | 2001-2024 | 7 (2.36%) | 166 | 2 (1.20%) | 6.92 | -16.7 | 3.79 | 4 | 7 | 0.167 |
| South Korea | Asia | 2002-2021 | 7 (2.36%) | 136 | 7 (5.15%) | 6.80 | -20.0 | 4.11 | 5 | 7 | 0.217 |
| India | Asia | 2020-2023 | 5 (1.69%) | 22 | 1 (4.55%) | 5.50 | 25.0 | 4.00 | 2 | 4 | 0.400 |
| Japan | Asia | 1999-2023 | 4 (1.35%) | 59 | 0 (0.00%) | 2.36 | -8.00 | 3.00 | 3 | 4 | 0.115 |
| Switzerland | Europe | 1988-2014 | 4 (1.35%) | 98 | 5 (5.10%) | 7.26 | -11.1 | 2.62 | 4 | 4 | 0.108 |
| Belgium | Europe | 2016-2017 | 2 (0.68%) | 15 | 0 (0.00%) | 7.50 | 0.00 | 1.33 | 2 | 2 | 0.222 |
| Brazil | South America | 2017-2024 | 2 (0.68%) | 5 | 0 (0.00%) | 0.63 | -12.5 | 1.50 | 1 | 2 | 0.125 |
| Ethiopia | Africa | 2022-2024 | 2 (0.68%) | 1 | 0 (0.00%) | 0.33 | -33.3 | 1.50 | 1 | 1 | 0.333 |
| Austria | Europe | 2019 | 1 (0.34%) | 1 | 0 (0.00%) | 1.00 | 0.00 | 0.33 | 1 | 1 | 0.167 |
| Colombia | South America | 2020 | 1 (0.34%) | 15 | 0 (0.00%) | 15.0 | 0.00 | 0.25 | 1 | 1 | 0.200 |
| Costa Rica | Central America | 2006 | 1 (0.34%) | 12 | 0 (0.00%) | 12.0 | 0.00 | 0.50 | 1 | 1 | 0.053 |
| Greece | Europe | 2016 | 1 (0.34%) | 7 | 0 (0.00%) | 7.00 | 0.00 | 0.33 | 1 | 1 | 0.111 |
| Laos | Asia | 2019 | 1 (0.34%) | 6 | 0 (0.00%) | 6.00 | 0.00 | 0.50 | 1 | 1 | 0.167 |
| Malawi | Africa | 2015 | 1 (0.34%) | 15 | 0 (0.00%) | 15.0 | 0.00 | 0.50 | 1 | 1 | 0.100 |
| Malaysia | Asia | 2001 | 1 (0.34%) | 24 | 0 (0.00%) | 24.0 | 0.00 | 0.50 | 1 | 1 | 0.042 |
| Mexico | Central America | 2020 | 1 (0.34%) | 15 | 0 (0.00%) | 15.0 | 0.00 | 0.25 | 1 | 1 | 0.200 |
| Morocco | Africa | 2018 | 1 (0.34%) | 7 | 0 (0.00%) | 7.00 | 0.00 | 0.50 | 1 | 1 | 0.143 |
| Nigeria | Africa | 2018 | 1 (0.34%) | 1 | 0 (0.00%) | 1.00 | 0.00 | 1.00 | 1 | 1 | 0.143 |
| Turkey | Europe | 2021 | 1 (0.34%) | 0 | 0 (0.00%) | 0.00 | 0.00 | 0.50 | 1 | 1 | 0.250 |

*Note*: AGR: annual growth rate. Please refer to the beginning of the supplemental materials for the definition of the bibliometric indices.

**Table MA2**. Spearman correlation between scientific production and country characteristics

| **Country characteristics** | **Spearman 's ρ** | **p** |
| --- | --- | --- |
| Scientific production per researchers in R&D (per million people) | **0.62** | **1.1e-04** |
| Scientific production per GDP | **0.46** | **5.9e-03** |
| Scientific production per cereal yield (kg per hectare) | **0.41** | **0.016** |
| Scientific production per female employment in agriculture (% of female employment) | **-0.69** | **6.6e-06** |
| Scientific production per employment in agriculture (% of total employment) | **-0.66** | **2.1e-05** |
| Scientific production per male employment in agriculture (% of male employment) | **-0.60** | **1.6e-04** |
| Scientific production per permanent cropland (% of land area) | **-0.53** | **1.5e-03** |
| Scientific production per agriculture, forestry, and fishing, value added (% of GDP) | **-0.51** | **2.1e-03** |
| Scientific production per fertilizer consumption (% of fertilizer production) | **-0.37** | **0.036** |
| Scientific production per fertilizer consumption (kilograms per hectare of arable land) | **-0.37** | **0.036** |
| Scientific production per livestock production index | -0.26 | 0.14 |
| Scientific production per food production index | -0.24 | 0.17 |
| Scientific production per cereal production (metric tons) | 0.20 | 0.25 |
| Scientific production per crop production index | -0.14 | 0.44 |
| Scientific production per land under cereal production (hectares) | 0.12 | 0.48 |
| Scientific production per population | -0.07 | 0.69 |
| Scientific production per agricultural land (% of land area) | -0.05 | 0.77 |
| Scientific production per total labor force | -0.03 | 0.85 |
| Scientific production per agricultural land (km^2^) | 0.01 | 0.96 |

*Note*: GDP: gross domestic product, R&D: research and development.

**Table MA3**. Spearman correlation between scientific production and country characteristics

| **Country** | **SciProd**  **per**  **GDP** | **SciProd**  **per**  **pop.** | **SciProd**  **per res.**  **in R&D (per**  **million**  **people)** | **SciProd**  **per**  **fertilizer**  **cons.**  **(% of**  **fertilizer**  **prod.)** | **SciProd**  **per**  **fertilizer**  **cons.**  **(kg per**  **hectare**  **of**  **arable**  **land)** | **SciProd**  **per**  **agricult.**  **land**  **(km^2^)** | **SciProd**  **per**  **agricult.**  **land**  **(% of**  **Land**  **area)** | **SciProd**  **per land**  **under**  **cereal**  **prod.**  **(hectares)** | **SciProd**  **per**  **permanent**  **cropland**  **(% of land**  **area)** | **SciProd**  **per**  **cereal**  **prod.**  **(metric**  **tons)** | **SciProd**  **per crop**  **prod.**  **index** | **SciProd**  **per food**  **prod.**  **index** | **SciProd**  **per**  **livestock**  **prod.**  **index** | **SciProd**  **per**  **cereal**  **yield**  **(kg per**  **hectare)** | **SciProd**  **per**  **female**  **employ.**  **in**  **agri.**  **(% of**  **female**  **employ.)** | **SciProd**  **per male**  **employ.**  **in**  **agri.**  **(% of**  **male**  **employ.)** | **SciProd**  **per**  **employ.**  **in**  **agri.**  **(% of**  **total**  **employ.)** | **SciProd**  **per**  **agri.,**  **forestry,**  **and**  **fishing,**  **value**  **added**  **(% of**  **GDP)** | **SciProd**  **per**  **total**  **labor**  **force** |
| --- | --- | --- | --- | --- | --- | --- | --- | --- | --- | --- | --- | --- | --- | --- | --- | --- | --- | --- | --- |
| USA | 3.6e-12 | 2.7e-07 | 0.02 | 0.67 | 0.67 | 2.0e-05 | 1.86 | 1.2e-06 | 3.1e+02 | 1.8e-07 | 0.85 | 0.87 | 0.82 | 0.01 | 59.3 | 22.8 | 32.8 | 68.1 | 5.3e-07 |
| France | 2.6e-11 | 1.1e-06 | 0.01 | 0.05 | 0.21 | 2.1e-04 | 1.13 | 7.2e-06 | 21.6 | 9.7e-07 | 0.67 | 0.66 | 0.63 | 9.4e-03 | 15.1 | 10.9 | 12.4 | 6.74 | 2.2e-06 |
| Finland | 1.3e-10 | 6.3e-06 | 4.5e-03 | 0.17 | 0.15 | 1.2e-03 | 3.69 | 2.6e-05 | 2.1e+03 | 8.2e-06 | 0.30 | 0.31 | 0.31 | 8.2e-03 | 5.85 | 3.32 | 4.19 | 3.57 | 1.2e-05 |
| Sweden | 5.3e-11 | 3.0e-06 | 3.9e-03 | 0.10 | 0.17 | 7.3e-04 | 2.98 | 1.9e-05 | 2.8e+03 | 4.5e-06 | 0.26 | 0.27 | 0.27 | 4.9e-03 | 16.6 | 5.99 | 8.83 | 6.17 | 5.3e-06 |
| Denmark | 7.1e-11 | 4.7e-06 | 3.6e-03 | 0.06 | 0.10 | 8.9e-04 | 0.35 | 1.5e-05 | 36.1 | 2.8e-06 | 0.26 | 0.27 | 0.26 | 3.9e-03 | 9.98 | 3.64 | 5.15 | 4.19 | 8.8e-06 |
| Norway | 4.7e-11 | 5.0e-06 | 4.0e-03 | 0.52 | 0.08 | 2.4e-03 | 8.71 | 7.3e-05 | 2.0e+03 | 1.7e-05 | 0.21 | 0.26 | 0.26 | 5.6e-03 | 7.54 | 3.54 | 4.67 | 5.29 | 9.0e-06 |
| Canada | 1.0e-11 | 5.7e-07 | 4.6e-03 | 0.30 | 0.17 | 3.5e-05 | 3.10 | 9.9e-07 | 1.1e+03 | 3.3e-07 | 0.20 | 0.20 | 0.19 | 5.2e-03 | 9.21 | 4.99 | 6.30 | 10.0 | 1.0e-06 |
| Australia | 9.0e-12 | 5.8e-07 | 3.3e-03 | 0.04 | 0.14 | 2.9e-06 | 0.22 | 7.3e-07 | 2.9e+02 | 2.7e-07 | 0.10 | 0.13 | 0.15 | 5.2e-03 | 3.91 | 2.33 | 2.80 | 3.58 | 1.1e-06 |
| UK | 3.9e-12 | 1.8e-07 | 2.6e-03 | 0.03 | 0.03 | 6.1e-05 | 0.15 | 3.0e-06 | 23.4 | 4.5e-07 | 0.11 | 0.12 | 0.11 | 1.5e-03 | 9.75 | 4.07 | 5.54 | 9.41 | 3.5e-07 |
| Italy | 5.5e-12 | 1.9e-07 | 4.1e-03 | 0.01 | 0.04 | 5.3e-05 | 0.16 | 1.7e-06 | 1.03 | 4.7e-07 | 0.08 | 0.09 | 0.10 | 1.9e-03 | 1.25 | 1.33 | 1.30 | 3.40 | 4.2e-07 |
| China | 5.6e-13 | 7.1e-09 | 6.3e-03 | 0.05 | 0.02 | 1.9e-06 | 0.18 | 9.7e-08 | 4.94 | 1.6e-08 | 0.09 | 0.09 | 0.09 | 1.6e-03 | 0.19 | 0.16 | 0.17 | 0.24 | 1.3e-08 |
| Iceland | 3.6e-10 | 2.6e-05 | 1.3e-03 | 4.8e-04 | 0.04 | 4.7e-04 | 0.47 | 3.3e-03 | no data | 1.1e-03 | 0.08 | 0.09 | 0.09 | 2.1e-03 | 2.02 | 0.68 | 0.98 | 1.14 | 4.3e-05 |
| Netherlands | 1.0e-11 | 5.7e-07 | 1.7e-03 | 0.12 | 0.01 | 4.4e-04 | 0.15 | 1.9e-05 | 6.89 | 4.6e-06 | 0.09 | 0.10 | 0.10 | 1.1e-03 | 3.54 | 1.92 | 2.33 | 1.78 | 1.0e-06 |
| New Zealand | 3.2e-11 | 1.6e-06 | 1.4e-03 | 0.02 | 3.6e-03 | 5.3e-05 | 0.14 | 3.0e-05 | 28.5 | 6.7e-06 | 0.07 | 0.08 | 0.08 | 8.9e-04 | 1.04 | 0.60 | 0.74 | 0.61 | 2.6e-06 |
| Spain | 5.7e-12 | 1.7e-07 | 2.6e-03 | 0.06 | 0.05 | 2.4e-05 | 0.12 | 1.0e-06 | 0.79 | 2.9e-07 | 0.07 | 0.07 | 0.07 | 1.8e-03 | 0.90 | 0.70 | 0.75 | 1.82 | 3.4e-07 |
| Germany | 1.7e-12 | 8.3e-08 | 1.3e-03 | 0.10 | 0.02 | 3.6e-05 | 0.13 | 8.9e-07 | 4.83 | 1.4e-07 | 0.07 | 0.07 | 0.06 | 8.7e-04 | 1.93 | 1.87 | 1.89 | 6.35 | 1.6e-07 |
| South Korea | 3.9e-12 | 1.4e-07 | 8.0e-04 | 7.7e-03 | 0.01 | 3.0e-04 | 0.29 | 3.0e-06 | 3.05 | 6.6e-07 | 0.06 | 0.07 | 0.06 | 1.0e-03 | 0.38 | 0.47 | 0.43 | 0.15 | 2.4e-07 |
| India | 1.5e-12 | 3.5e-09 | 0.02 | 0.02 | 0.02 | 2.8e-06 | 0.08 | 4.7e-08 | 1.09 | 1.4e-08 | 0.04 | 0.04 | 0.04 | 1.4e-03 | 0.06 | 0.09 | 0.08 | 0.12 | 8.4e-09 |
| Japan | 9.5e-13 | 3.2e-08 | 7.3e-04 | 0.03 | 8.3e-03 | 5.6e-05 | 0.21 | 8.2e-07 | 2.30 | 1.9e-07 | 0.03 | 0.04 | 0.04 | 5.9e-04 | 0.51 | 0.67 | 0.59 | 2.11 | 5.8e-08 |
| Switzerland | 5.0e-12 | 4.6e-07 | 7.2e-04 | 3.9e-03 | 8.2e-03 | 2.3e-04 | 0.09 | 1.9e-05 | 6.19 | 2.8e-06 | 0.03 | 0.04 | 0.04 | 5.7e-04 | 0.98 | 0.66 | 0.79 | 1.90 | 8.0e-07 |
| Belgium | 3.5e-12 | 1.7e-07 | 3.5e-04 | 0.03 | 3.4e-03 | 1.1e-04 | 0.04 | 5.7e-06 | 2.52 | 6.1e-07 | 0.02 | 0.02 | 0.02 | 2.0e-04 | 0.92 | 0.59 | 0.69 | 1.49 | 3.7e-07 |
| Brazil | 1.0e-12 | 9.3e-09 | 2.3e-03 | 2.5e-03 | 5.4e-03 | 8.3e-07 | 0.07 | 7.2e-08 | 1.43 | 1.5e-08 | 0.02 | 0.02 | 0.02 | 3.8e-04 | 0.18 | 0.08 | 0.11 | 0.12 | 1.8e-08 |
| Ethiopia | 1.6e-11 | 1.6e-08 | 0.02 | no data | 0.05 | 3.4e-06 | 0.04 | 1.8e-07 | 0.99 | 6.3e-08 | 0.02 | 0.02 | 0.02 | 6.9e-04 | 0.03 | 0.02 | 0.03 | 0.03 | 3.2e-08 |
| Austria | 2.1e-12 | 1.1e-07 | 1.7e-04 | 7.0e-03 | 3.6e-03 | 2.8e-05 | 0.02 | 9.4e-07 | 0.83 | 1.7e-07 | 8.3e-03 | 9.7e-03 | 9.9e-03 | 1.4e-04 | 0.12 | 0.15 | 0.14 | 0.20 | 2.1e-07 |
| Colombia | 2.9e-12 | 1.9e-08 | 0.01 | 7.0e-04 | 1.4e-03 | 2.2e-06 | 0.02 | 5.7e-07 | 0.44 | 2.2e-07 | 9.0e-03 | 9.0e-03 | 9.1e-03 | 2.3e-04 | 0.13 | 0.03 | 0.05 | 0.04 | 3.9e-08 |
| Costa Rica | 1.5e-11 | 1.9e-07 | 1.8e-03 | 4.3e-05 | 1.1e-03 | 3.6e-05 | 0.02 | 6.1e-06 | 0.14 | 2.6e-06 | 9.5e-03 | 9.5e-03 | 8.8e-03 | 2.1e-04 | 0.10 | 0.04 | 0.05 | 0.04 | 3.9e-07 |
| Greece | 4.6e-12 | 9.5e-08 | 2.5e-04 | 1.6e-03 | 4.1e-03 | 1.1e-05 | 0.01 | 5.6e-07 | 0.11 | 1.6e-07 | 8.7e-03 | 9.2e-03 | 8.6e-03 | 2.2e-04 | 0.04 | 0.06 | 0.05 | 0.14 | 2.0e-07 |
| Laos | 6.5e-11 | 1.3e-07 | 0.06 | no data | 0.02 | 4.3e-05 | 0.10 | 8.1e-07 | 1.37 | 1.8e-07 | 8.8e-03 | 8.7e-03 | 5.5e-03 | 2.2e-04 | 0.01 | 0.01 | 0.01 | 0.02 | 3.2e-07 |
| Malawi | 7.6e-11 | 4.9e-08 | 0.02 | 1.0e-05 | 0.01 | 1.7e-05 | 0.02 | 4.7e-07 | 0.47 | 2.0e-07 | 7.4e-03 | 6.8e-03 | 5.3e-03 | 4.1e-04 | 0.01 | 0.01 | 0.01 | 0.03 | 1.2e-07 |
| Malaysia | 2.5e-12 | 3.0e-08 | 4.2e-04 | 5.0e-04 | 4.4e-04 | 1.2e-05 | 0.04 | 1.3e-06 | 0.04 | 3.4e-07 | 9.4e-03 | 9.7e-03 | 9.5e-03 | 2.2e-04 | 0.07 | 0.05 | 0.05 | 0.02 | 5.8e-08 |
| Mexico | 7.1e-13 | 7.8e-09 | 2.4e-03 | 1.9e-03 | 9.2e-03 | 9.4e-07 | 0.02 | 8.7e-08 | 0.69 | 2.6e-08 | 8.7e-03 | 8.5e-03 | 8.3e-03 | 2.5e-04 | 0.09 | 0.03 | 0.04 | 0.08 | 1.7e-08 |
| Morocco | 7.5e-12 | 2.7e-08 | 9.3e-04 | 3.5e-03 | 0.01 | 3.2e-06 | 0.01 | 1.6e-07 | 0.25 | 8.6e-08 | 8.4e-03 | 8.6e-03 | 8.6e-03 | 4.3e-04 | 0.02 | 0.02 | 0.02 | 0.04 | 8.1e-08 |
| Nigeria | 2.1e-12 | 4.6e-09 | 0.03 | 1.6e-04 | 0.05 | 1.5e-06 | 0.01 | 5.2e-08 | 0.14 | 3.3e-08 | 8.3e-03 | 8.3e-03 | 8.9e-03 | 5.8e-04 | 0.02 | 0.02 | 0.02 | 0.03 | 1.3e-08 |
| Turkey | 1.1e-12 | 1.2e-08 | 5.6e-04 | 2.1e-03 | 6.7e-03 | 2.4e-06 | 0.02 | 7.1e-08 | 0.21 | 2.6e-08 | 8.0e-03 | 7.8e-03 | 7.3e-03 | 2.9e-04 | 0.01 | 0.03 | 0.02 | 0.02 | 2.9e-08 |

*Note*: agri.: agriculture, agricult.: agricultural, cons.: consumption, employ.: employment, GDP: gross domestic product, pop.: population, prod.: production, R&D: research and development, res.: researcher, SciProd: scientific production.

- *The most active journals*

Articles were published in 118 journals (Table 4). Most journals published less than five studies and received on average 3.86 citations. The most active journals were the *American Journal Of Industrial Medicine* (18/296, 6.1%), the *Journal Of Occupational And Environmental Medicine* (14/296, 4.7%), as well as *the International Journal Of Epidemiology*, the *Occupational And Environmental Medicine*, and the *Scandinavian Journal Of Work Environment & Health*, which all had 13 publications (Table MA4). Notably, 11 of the top 20 journals are ranked in the first quartile (Q1) of their respective category, primarily in public health and occupational health, with 2022 impact factors ranging from 2.3 to 12.7.

**Table MA4**. Top 20 of the most active journals.

| **Journal** | **Publication**  **start** | **Number of**  **publications (%)** | **Total**  **citation** | **h-index** | **Average citation**  **per year** | **Mean AGR** | **g-index** | **m-index** | **Impact factor**  **(2022)** | **Rank** |
| --- | --- | --- | --- | --- | --- | --- | --- | --- | --- | --- |
| American Journal Of Industrial Medicine | 1989 | 18 (6.08%) | 515 | 15 | 14.3 | -27.8 | 18 | 0.42 | 3.5 | Q2 |
| Journal Of Occupational And Environmental Medicine | 1985 | 14 (4.73%) | 423 | 12 | 10.6 | -20.8 | 14 | 0.30 | 3.2 | Q3 |
| International Journal Of Epidemiology | 1980 | 13 (4.39%) | 813 | 13 | 18.1 | -14.2 | 13 | 0.29 | 7.7 | Q1 |
| Occupational And Environmental Medicine | 1995 | 13 (4.39%) | 351 | 9 | 11.7 | -20.0 | 13 | 0.30 | 4.9 | Q1 |
| Scandinavian Journal Of Work Environment & Health | 1990 | 13 (4.39%) | 649 | 12 | 18.5 | -20.6 | 13 | 0.34 | 6.3 | Q1 |
| Cancer Causes & Control | 1997 | 9 (3.04%) | 223 | 8 | 7.96 | -14.8 | 9 | 0.29 | 2.3 | Q4 |
| Environmental Research | 1993 | 9 (3.04%) | 76 | 4 | 2.38 | -6.25 | 8 | 0.13 | 8.3 | Q1 |
| Environmental Health Perspectives | 2006 | 7 (2.36%) | 357 | 7 | 18.8 | -26.7 | 7 | 0.37 | 10.5 | Q1 |
| International Journal Of Environmental Research And Public Health | 2011 | 7 (2.36%) | 56 | 3 | 4.00 | -11.5 | 7 | 0.21 | 4.6 | Q2 |
| International Archives Of Occupational And Environmental Health | 2001 | 6 (2.03%) | 118 | 6 | 4.92 | -23.5 | 6 | 0.25 | 3.0 | Q3 |
| International Journal Of Cancer | 1989 | 6 (2.03%) | 153 | 4 | 4.25 | -11.8 | 6 | 0.11 | 6.4 | Q1 |
| Journal Of Agromedicine | 2009 | 6 (2.03%) | 98 | 5 | 6.13 | 12.5 | 6 | 0.31 | 2.4 | Q3 |
| American Journal Of Epidemiology | 1993 | 5 (1.69%) | 669 | 5 | 20.9 | -8.00 | 5 | 0.16 | 5.0 | Q1 |
| Journal Of Epidemiology And Community Health | 1981 | 5 (1.69%) | 264 | 5 | 6.00 | -31.2 | 5 | 0.11 | 6.3 | Q1 |
| British Journal Of Industrial Medicine | 1988 | 5 (1.69%) | 256 | 5 | 6.92 | -30 | 5 | 0.14 | none | none |
| BMJ Open | 2013 | 4 (1.35%) | 35 | 3 | 2.92 | -28.6 | 4 | 0.25 | 2.9 | Q2 |
| Frontiers In Public Health | 2022 | 4 (1.35%) | 8 | 2 | 2.67 | 0.00 | 2 | 0.67 | 5.2 | Q1 |
| American Journal Of Public Health | 1990 | 3 (1.01%) | 144 | 3 | 4.11 | -5.00 | 3 | 0.09 | 12.7 | Q1 |
| Annals Of Epidemiology | 2010 | 3 (1.01%) | 133 | 3 | 8.87 | -22.2 | 3 | 0.20 | 5.6 | Q1 |
| Cancer Epidemiology | 2017 | 3 (1.01%) | 40 | 2 | 5.00 | -14.3 | 3 | 0.25 | 2.6 | Q2 |

*Note*: AGR: annual growth rate. Please refer to the beginning of the supplemental materials for the definition of the bibliometric indices.

- *The most prolific authors*

The top 25 of the most prolific authors included 28 individuals who contributed to at least 7 (7/296, 2.4%) publications (Table MA5). Among these authors, only nine were females (9/28, 32%), and twelve (12/28, 43%) published their last paper more than three years ago. I. Baldi led in total publications (18/296, 6.1%), followed closely by P. Lebailly and E.I. Pukkala, each with 16 publications (16/296, 5.4%). In terms of first authorship, P.K. Mills had a dominant presence, achieving a first author contribution of 100%, followed by P. Petit (85.7%), S. Jouneau (42.9%), and C. Piel (42.9%). D.P. Sandler emerged as the most cited author in this group, with 943 citations, followed by E.I. Pukkala (932) and A. Blair (905). The majority of the articles published by these authors focused on cancer risk (23/28, 82%), with additional research on neurodegenerative disorders (8/28, 29%), particularly Parkinson's disease (7/28, 25%), depression (6/28, 21%), and respiratory conditions (5/28, 18%).

**Table MA5**. Top 25 of the most prolific authors.

| **Author** | **Sex** | **Publication**  **period** | **Number of**  **publications** | **FF**  (%) | **DF1**  (%) | **DF**  **last**  (%) | **Total**  **citation** | **Local**  **citation** | **Average**  **citation**  **per year** | **h-**  **index** | **Mean**  **AGR**  (%) | **g-**  **index** | **m-**  **index** | **Y-**  **index** | **Thematic** |
| --- | --- | --- | --- | --- | --- | --- | --- | --- | --- | --- | --- | --- | --- | --- | --- |
| BALDI I | F | 2011-2024 | 18 (6.1%) | 1.61 | 5.56 | 27.8 | 494 | 33 (6.7%) | 35.3 | 10 | 3.57 | 18 | 0.71 | 5.10 | Cancer, neurobehavior effect, aging, mortality, PD |
| LEBAILLY P | M | 2011-2024 | 16 (5.4%) | 1.45 | 0.00 | 37.5 | 361 | 32 (8.9%) | 25.8 | 10 | 12.5 | 16 | 0.71 | 6.00 | Cancer, neurobehavior effect, mortality, PD |
| PUKKALA EI | M | 1997-2023 | 16 (5.4%) | 2.66 | 12.5 | 62.5 | 932 | 25 (2.7%) | 34.5 | 10 | -20.4 | 16 | 0.36 | 10.2 | Cancer |
| LYNGE E | F | 1988-2024 | 15 (5.1%) | 3.27 | 20.0 | 13.3 | 418 | 5 (1.2%) | 11.3 | 9 | -17.6 | 15 | 0.24 | 3.61 | Cancer |
| TUAL S | F | 2017-2022 | 13 (4.4%) | 1.10 | 15.4 | 0.00 | 358 | 27 (7.5%) | 59.7 | 9 | 0.00 | 13 | 1.12 | 2.00 | Cancer, PD |
| BLAIR A | M | 1981-2017 | 11 (3.7%) | 2.02 | 18.2 | 27.3 | 905 | 22 (2.4%) | 24.5 | 10 | -16.2 | 11 | 0.23 | 3.61 | Cancer |
| SANDLER DP | M | 2003-2020 | 11 (3.7%) | 1.27 | 0.00 | 0.00 | 943 | 17 (1.8%) | 52.4 | 11 | -22.2 | 11 | 0.50 | 0.00 | Cancer, CVD, injury |
| BOULANGER M | F | 2017-2022 | 10 (3.4%) | 0.93 | 20.0 | 0.00 | 151 | 11 (7.3%) | 25.2 | 7 | 5.56 | 10 | 0.88 | 2.00 | Cancer, mortality |
| KJAERHEIM K | F | 1999-2023 | 10 (3.4%) | 1.21 | 20.0 | 10.0 | 839 | 29 (3.5%) | 33.6 | 8 | -12.0 | 10 | 0.31 | 2.24 | Cancer |
| DEGANO B | M | 2016-2020 | 9 (3.0%) | 1.00 | 0.00 | 44.4 | 123 | 12 (9.8%) | 24.6 | 5 | 16.7 | 9 | 0.56 | 4.00 | COPD, depression |
| ELBAZ A | M | 2003-2021 | 9 (3.0%) | 1.45 | 22.2 | 55.6 | 549 | 8 (1.5%) | 28.9 | 8 | -26.3 | 9 | 0.36 | 5.39 | PD, motor neuron disease |
| LAPLANTE JJ | M | 1998-2019 | 9 (3.0%) | 0.85 | 0.00 | 0.00 | 210 | 16 (7.6%) | 9.55 | 7 | 11.4 | 9 | 0.26 | 0.00 | Asthma, cancer, COPD, depression |
| MARCOTULLIO E | F | 2017-2022 | 9 (3.0%) | 0.72 | 0.00 | 0.00 | 181 | 20 (11.0%) | 30.2 | 8 | 4.17 | 9 | 1.00 | 0.00 | Cancer, PD |
| MARTINSEN JI | M | 2009-2023 | 9 (3.0%) | 1.37 | 0.00 | 0.00 | 573 | 24 (4.2%) | 38.2 | 8 | -20.0 | 9 | 0.50 | 0.00 | Cancer |
| BONNETERRE V | M | 2020-2024 | 8 (2.7%) | 1.79 | 12.5 | 87.5 | 28 | 16 (57.1%) | 5.60 | 3 | 30.0 | 5 | 1.00 | 7.07 | AD, cancer, depression, IBD, thyroid disorders |
| DALPHIN JC | M | 1998-2020 | 8 (2.7%) | 0.86 | 12.5 | 50.0 | 195 | 11 (5.6%) | 8.48 | 6 | -4.35 | 8 | 0.22 | 4.12 | Asthma, cancer, COPD, depression |
| FREEMAN LEB | F | 2013-2023 | 8 (2.7%) | 0.75 | 0.00 | 37.5 | 380 | 10 (2.6%) | 34.6 | 7 | -22.7 | 8 | 0.58 | 3.00 | Cancer, injury |
| HOPPIN JA | F | 2003-2017 | 8 (2.7%) | 0.88 | 0.00 | 25.0 | 803 | 15 (1.9%) | 53.5 | 8 | -20.0 | 8 | 0.36 | 2.00 | Cancer, injury |
| SOUMAGNE TE | M | 2016-2020 | 8 (2.7%) | 0.88 | 12.5 | 0.00 | 120 | 12 (10.0%) | 24.0 | 5 | 0.00 | 8 | 0.56 | 1.00 | COPD, depression |
| PETIT P | M | 2022-2024 | 7 (2.4%) | 1.63 | 85.7 | 14.3 | 21 | 14 (66.7%) | 7.00 | 3 | 83.3 | 4 | 1.00 | 6.08 | AD, cancer, depression, IBD, thyroid disorders |
| ALAVANJA MCR | M | 2003-2017 | 7 (2.4%) | 0.77 | 28.6 | 57.1 | 752 | 17 (2.3%) | 50.1 | 7 | -13.3 | 7 | 0.32 | 4.47 | Cancer, injury |
| JOUNEAU S | M | 2015-2022 | 7 (2.4%) | 0.55 | 42.9 | 0.00 | 69 | 6 (8.7%) | 8.63 | 4 | -25.0 | 7 | 0.40 | 3.00 | Asthma, COPD |
| KOUTROS S | F | 2010-2020 | 7 (2.4%) | 0.67 | 0.00 | 0.00 | 369 | 6 (1.6%) | 33.6 | 6 | -9.09 | 7 | 0.40 | 0.00 | Cancer |
| LYNCH CF | M | 2003-2023 | 7 (2.4%) | 0.75 | 0.00 | 0.00 | 744 | 15 (2.0%) | 35.4 | 6 | -19.0 | 7 | 0.27 | 0.00 | Cancer |
| MILLS PK | M | 2001-2009 | 7 (2.4%) | 3.17 | 100 | 0.00 | 327 | 6 (1.8%) | 36.3 | 7 | -44.4 | 7 | 0.29 | 7.00 | Cancer |
| NORDBY KC | M | 2004-2023 | 7 (2.4%) | 0.90 | 28.6 | 0.00 | 183 | 11 (6.0%) | 9.15 | 5 | -20.0 | 7 | 0.24 | 2.00 | Cancer |
| PIEL C | M | 2017-2022 | 7 (2.4%) | 0.51 | 42.9 | 0.00 | 200 | 9 (4.5%) | 33.3 | 6 | 8.33 | 7 | 0.75 | 3.00 | Cancer, PD |
| TZOURIO C | M | 2003-2015 | 7 (2.4%) | 0.95 | 0.00 | 42.9 | 538 | 7 (1.3%) | 41.4 | 7 | -23.1 | 7 | 0.32 | 3.00 | PD |

*Note*: AD: Alzheimer’s disease, AGR: annual growth rate, COPD: chronic obstructive pulmonary disease, CVD: cardiovascular disorders, DF: dominance factor, DF1: dominance factor for being first author, DF last: dominance factor for being last author, F: female, FF: fractionalized frequency, IBD: inflammatory bowel disease, M: male, PD: Parkinson’s disease.

- *The most active institutions*

There were 338 institutions reported. The top 20 most active institutions comprised 24 organizations that contributed eleven or more publications (11/296, 3.7%) (Table MA6). These institutions were spread across six countries, with 13 from France (13/24, 54%), four from the US (4/24, 17%), three from Finland (3/24, 13%), two from Denmark (2/24, 8%), and one each from Sweden and Norway (1/24, 4%). INSERM (France) led with the highest number of publications (47/296, 15.9%), followed by Karolinska Institutet (Sweden) (24/296, 8.1%), and the University of Oslo (Norway) (23/296, 7.8%). INSERM was the most cited (1390/9379, 14.8%), followed closely by NIH (US) and NCI (US), each with 1336 citations (1336/9379, 14.2%). Among the top 20, CHU Bordeaux (35.3%), Université Grenoble Alpes (25.0%), and CHU Grenoble Alpes (25.0%) recorded the highest mean AGR.

**Table MA6**. Top 20 of the most active institutions.

| **Institution** | **Country** | **Publication period** | **Number of publications (%)** | **Total citation** | **Local citation (%)** | **Average citation**  **per year** | **Mean AGR** | **Fractionalized frequency (%)** | **h-index** | **g-index** | **m-index** |
| --- | --- | --- | --- | --- | --- | --- | --- | --- | --- | --- | --- |
| INSERM | France | 2003-2024 | 47 (15.9%) | 1390 | 47 (3.38%) | 63.2 | 6.19 | 7.73 | 20 | 37 | 0.91 |
| Karolinska Institutet | Sweden | 1987-2024 | 24 (8.11%) | 1214 | 29 (2.67%) | 31.9 | -23.2 | 7.53 | 15 | 24 | 0.40 |
| University of Oslo | Norway | 1996-2024 | 23 (7.77%) | 1137 | 35 (3.93%) | 39.2 | -17.2 | 5.71 | 12 | 23 | 0.41 |
| Université de Bordeaux | France | 2011-2024 | 22 (7.43%) | 566 | 34 (6.01%) | 40.4 | 6.43 | 2.96 | 13 | 22 | 0.93 |
| National Institutes of Health (NIH) | US | 1998-2023 | 21 (7.09%) | 1336 | 29 (2.17%) | 51.4 | -19.2 | 4.24 | 17 | 21 | 0.63 |
| National Cancer Institute (NCI) | US | 1998-2023 | 21 (7.09%) | 1336 | 29 (2.17%) | 51.4 | -19.2 | 4.24 | 17 | 21 | 0.63 |
| CHU Bordeaux | France | 2012-2023 | 19 (6.42%) | 500 | 33 (6.60%) | 41.7 | 35.3 | 2.38 | 11 | 19 | 0.85 |
| Unicancer | France | 2011-2023 | 19 (6.42%) | 479 | 32 (6.68%) | 36.8 | -12.8 | 2.28 | 11 | 19 | 0.79 |
| Université de Caen-Normandie | France | 2011-2024 | 18 (6.08%) | 450 | 33 (7.33%) | 32.1 | 2.38 | 2.35 | 10 | 18 | 0.71 |
| Centre François Baclesse | France | 2011-2023 | 16 (5.41%) | 429 | 28 (6.53%) | 33.0 | -12.8 | 1.95 | 10 | 16 | 0.71 |
| Finnish Cancer Registry | Finland | 2005-2024 | 16 (5.41%) | 864 | 25 (3.80%) | 43.2 | -17.3 | 2.95 | 10 | 16 | 0.39 |
| Assistance Publique – Hôpitaux de Paris (AP-HP) | France | 2003-2023 | 15 (5.07%) | 571 | 7 (1.23%) | 27.2 | -14.3 | 2.21 | 9 | 15 | 0.41 |
| University of Copenhagen | Denmark | 1997-2024 | 15 (5.07%) | 621 | 24 (3.86%) | 22.2 | -14.3 | 2.57 | 8 | 15 | 0.29 |
| CNRS | France | 2011-2024 | 14 (4.73%) | 152 | 5 (3.29%) | 10.9 | -7.14 | 2.32 | 4 | 12 | 0.29 |
| Tampere university | Finland | 1980-2024 | 14 (4.73%) | 886 | 24 (2.71%) | 19.7 | -11.1 | 2.92 | 9 | 14 | 0.20 |
| Université de Franche-Comté | France | 1998-2020 | 13 (4.39%) | 257 | 15 (5.84%) | 11.2 | -7.97 | 2.74 | 9 | 13 | 0.33 |
| Finnish Institute of Occupational Health | Finland | 1997-2020 | 12 (4.05%) | 518 | 3 (0.58%) | 21.6 | -16.7 | 6.83 | 10 | 12 | 0.36 |
| NIEHS | US | 2003-2020 | 12 (4.05%) | 1086 | 17 (1.57%) | 60.3 | -25.0 | 2.34 | 12 | 12 | 0.56 |
| Sorbonne Université | France | 2003-2019 | 12 (4.05%) | 595 | 9 (1.51%) | 35.0 | -20.6 | 1.68 | 10 | 12 | 0.46 |
| Université Grenoble Alpes (UGA) | France | 2019-2024 | 11 (3.72%) | 35 | 14 (40.0%) | 5.83 | 25.0 | 1.56 | 4 | 5 | 1.33 |
| CHU Grenoble Alpes | France | 2019-2024 | 11 (3.72%) | 35 | 14 (40.0%) | 5.83 | 25.0 | 1.56 | 4 | 5 | 1.33 |
| CHU Besançon | France | 1998-2019 | 11 (3.72%) | 247 | 15 (6.07%) | 11.2 | -9.85 | 2.58 | 9 | 11 | 0.33 |
| Danish Cancer Society | Denmark | 1988-2024 | 11 (3.72%) | 323 | 0 (0.00%) | 8.73 | -13.5 | 4.83 | 7 | 11 | 0.19 |
| University of Iowa | US | 2002-2023 | 11 (3.72%) | 829 | 19 (2.29%) | 37.7 | -18.2 | 3.78 | 8 | 11 | 0.35 |

*Note*: AGR: annual growth rate, CNRS: Centre national de la recherche scientifique (French National Centre for Scientific Research), INSERM: Institut national de la santé et de la recherche médicale (French National Institute of Health and Medical Research), NIEHS: National Institute of Environmental Health Sciences.

Please refer to the beginning of the supplemental materials for the definition of the bibliometric indices.

- *The most active funding bodies*

Funding sources were not reported for 156 (156/296, 52.7%) publications. Among the top 20 of the most active funding bodies, which contributed to 18 or more publications (18/296, 6.1%), the NIH (US) led with 22 publications (22/296, 7.4%), followed by Mutualité Sociale Agricole (MSA) (France) (19/296, 6.4%), and Medline (US) (18/296, 6.1%). MIAI@Grenoble Alpes (France) (83.3%), the French national cancer institute (INCa) (42.5%), and Ligue Contre Le Cancer (France) (37.5%) demonstrated the highest mean AGR among this top 20 (Table MA7).

**Table MA7**. Top 20 of the most active funding bodies.

| **Funding bodies** | **Country** | **Publication**  **period** | **Number of**  **publications**  **(%)** | **Total citation** | **Local**  **citation**  **(%)** | **Average**  **citation**  **per year** | **Mean**  **AGR** | **Fractionalized**  **frequency (%)** | **h-index** | **g-index** | **m-index** |
| --- | --- | --- | --- | --- | --- | --- | --- | --- | --- | --- | --- |
| National Institutes Of Health (NIH) | US | 1985-2022 | 22 (7.43%) | 840 | 16 (2.02%) | 22.1 | -11.0 | 10.0 | 18 | 22 | 0.45 |
| Mutualité Sociale Agricole (MSA) | France | 2009-2023 | 19 (6.42%) | 632 | 27 (4.27%) | 42.1 | 8.33 | 4.51 | 11 | 19 | 0.69 |
| Medline | US | 1985-2020 | 18 (6.08%) | 664 | 14 (2.27%) | 18.4 | -19.9 | 9.17 | 15 | 18 | 0.38 |
| French National Agency for Research (ANR) | France | 2009-2024 | 15 (5.07%) | 423 | 15 (3.55%) | 26.4 | -1.56 | 3.87 | 6 | 15 | 0.38 |
| French national cancer institute (INCa) | France | 2015-2022 | 15 (5.07%) | 333 | 21 (6.31%) | 41.6 | 42.5 | 3.71 | 10 | 15 | 1.00 |
| National Institute for Occupational Safety & Health (NIOSH) | US | 2005-2022 | 15 (5.07%) | 295 | 13 (4.41%) | 16.4 | -13.9 | 8.21 | 8 | 15 | 0.40 |
| ANSES | France | 2010-2022 | 14 (4.73%) | 278 | 25 (8.99%) | 21.4 | 0.00 | 3.26 | 9 | 14 | 0.60 |
| Centre François Baclesse | France | 2017-2022 | 12 (4.05%) | 291 | 26 (8.93%) | 48.5 | 0.00 | 1.31 | 9 | 12 | 1.12 |
| Ligue Contre Le Cancer | France | 2017-2022 | 12 (4.05%) | 272 | 25 (9.19%) | 45.3 | 37.5 | 1.61 | 9 | 12 | 1.12 |
| Association Pour La Recherche Sur Le Cancer (ARC) | France | 2017-2022 | 11 (3.72%) | 268 | 20 (7.46%) | 44.7 | 3.33 | 1.17 | 9 | 11 | 1.12 |
| Fondation De France | France | 2017-2022 | 11 (3.72%) | 268 | 20 (7.46%) | 44.7 | 3.33 | 1.17 | 9 | 11 | 1.12 |
| French National Institute of Agricultural Medicine (INMA) | France | 2017-2022 | 11 (3.72%) | 270 | 26 (9.63%) | 45.0 | 2.78 | 1.18 | 9 | 11 | 1.12 |
| French Ministry of Higher education and scientific research | France | 2009-2024 | 10 (3.38%) | 433 | 16 (3.70%) | 27.1 | -25.0 | 1.47 | 8 | 10 | 0.50 |
| French National Office for Water and Aquatic Environments (Onema) | France | 2017-2022 | 9 (3.04%) | 189 | 25 (13.2%) | 31.5 | 5.56 | 1.69 | 6 | 9 | 0.75 |
| [Miai@Grenoble Alpes](mailto:Miai@Grenoble%20Alpes) | France | 2022-2024 | 7 (2.36%) | 21 | 14 (66.7%) | 7.00 | 83.3 | 2.83 | 3 | 4 | 1.00 |
| National Institute Of Environmental Health Sciences (NIEHS) | US | 2010-2020 | 7 (2.36%) | 397 | 0 (0.00%) | 36.1 | -22.7 | 2.62 | 6 | 7 | 0.40 |
| Novartis Pharma | Switzerland | 2018-2022 | 7 (2.36%) | 33 | 4 (12.1%) | 6.60 | 13.3 | 2.62 | 4 | 5 | 0.57 |
| National Cancer Institute (NCI) | US | 2014-2023 | 6 (2.03%) | 316 | 1 (0.32%) | 31.6 | -20.0 | 1.70 | 4 | 6 | 0.36 |
| Nordic Cancer Union | Nordic  countries | 2009-2023 | 6 (2.03%) | 514 | 21 (4.09%) | 34.3 | -20.0 | 3.27 | 4 | 6 | 0.25 |
| Centers for Disease Control and Prevention (CDC) | US | 2009-2022 | 5 (1.69%) | 32 | 0 (0.00%) | 2.29 | -21.4 | 1.54 | 3 | 5 | 0.19 |

*Note*: AGR: annual growth rate, ANSES: French Agency for Food, Environmental and Occupational Health & Safety.

Please refer to the beginning of the supplemental materials for the definition of the bibliometric indices.

# **Multimedia appendix 2**. Publications included and analyzed.

Please refer to the MS Excel file entitled “Multimedia appendix 2”.

# **Figure S5**. Number of publications per country.


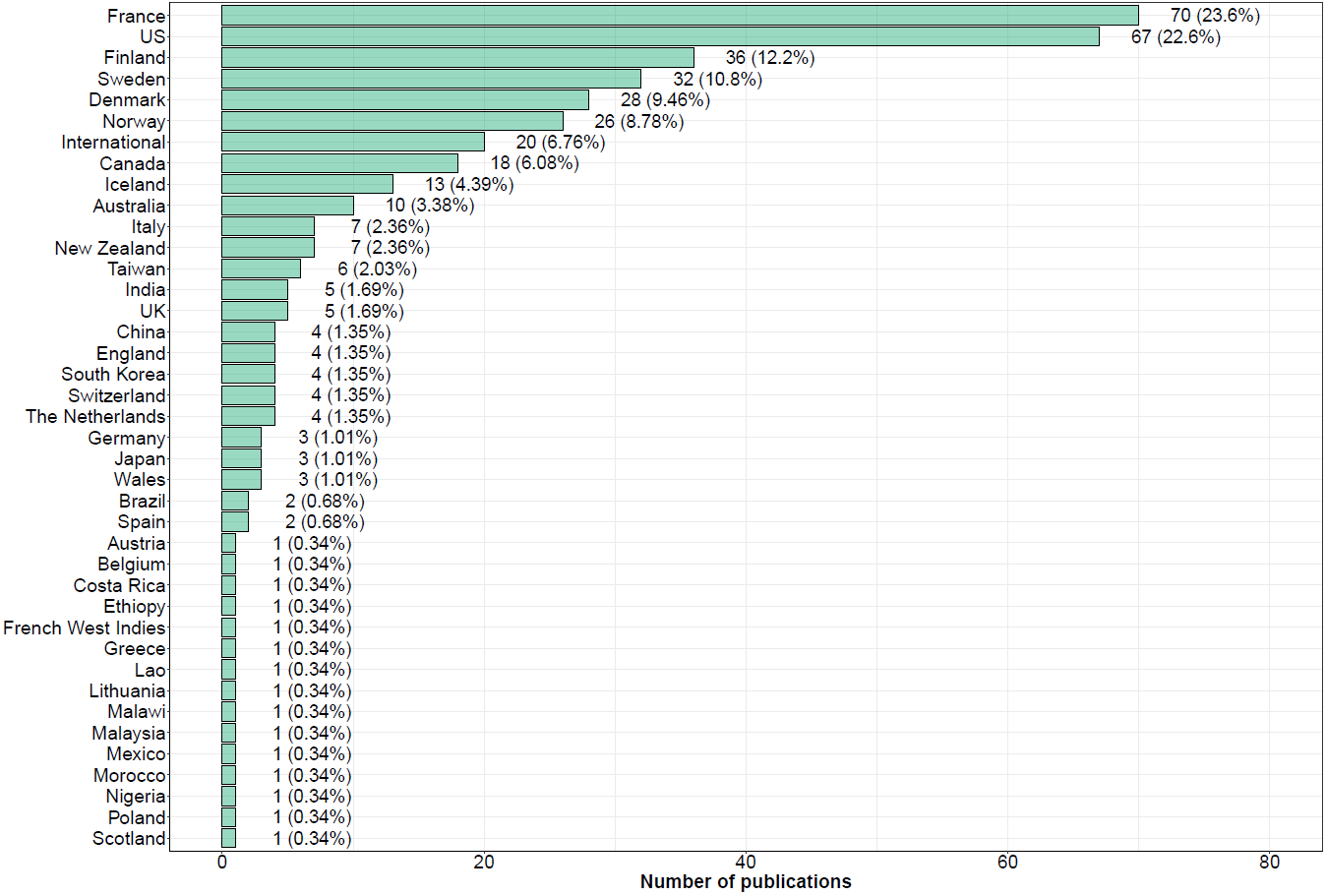


# **Figure S6**. Main goal addressed by study.


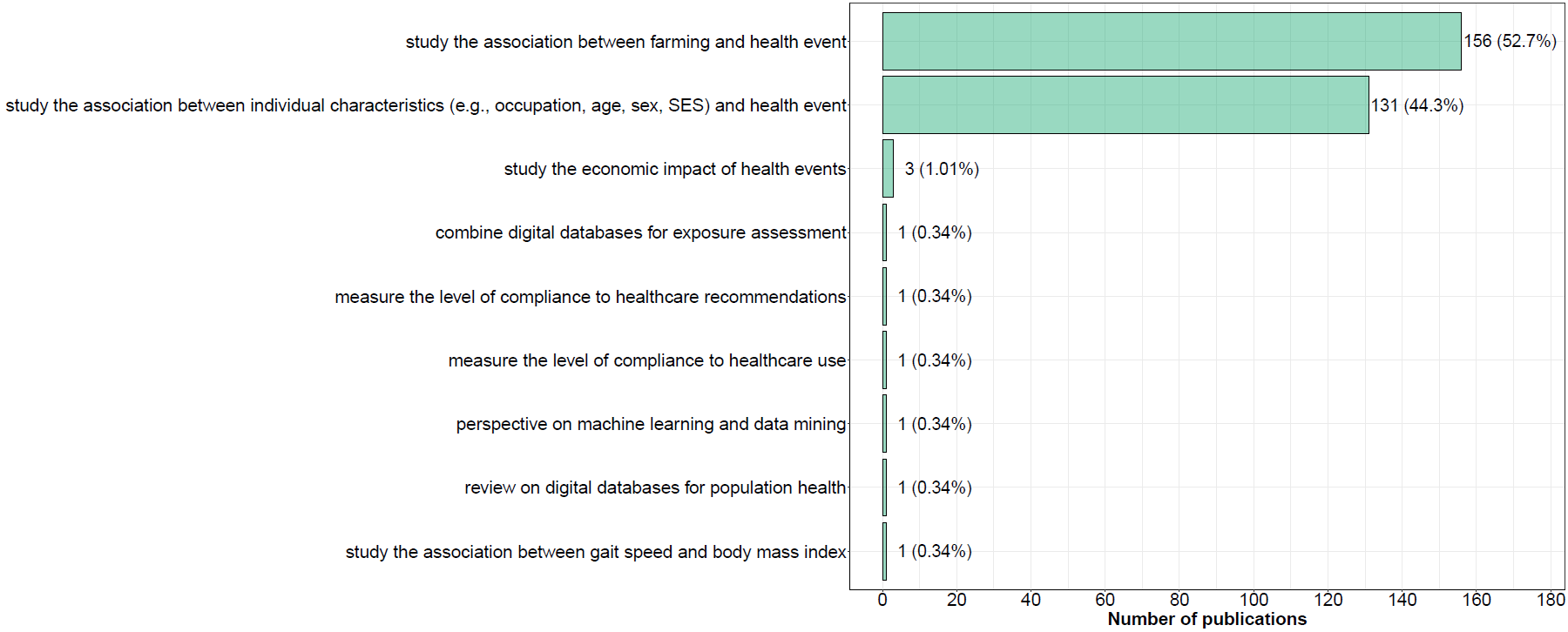


# **Figure S7**. Number of publications per cohort.


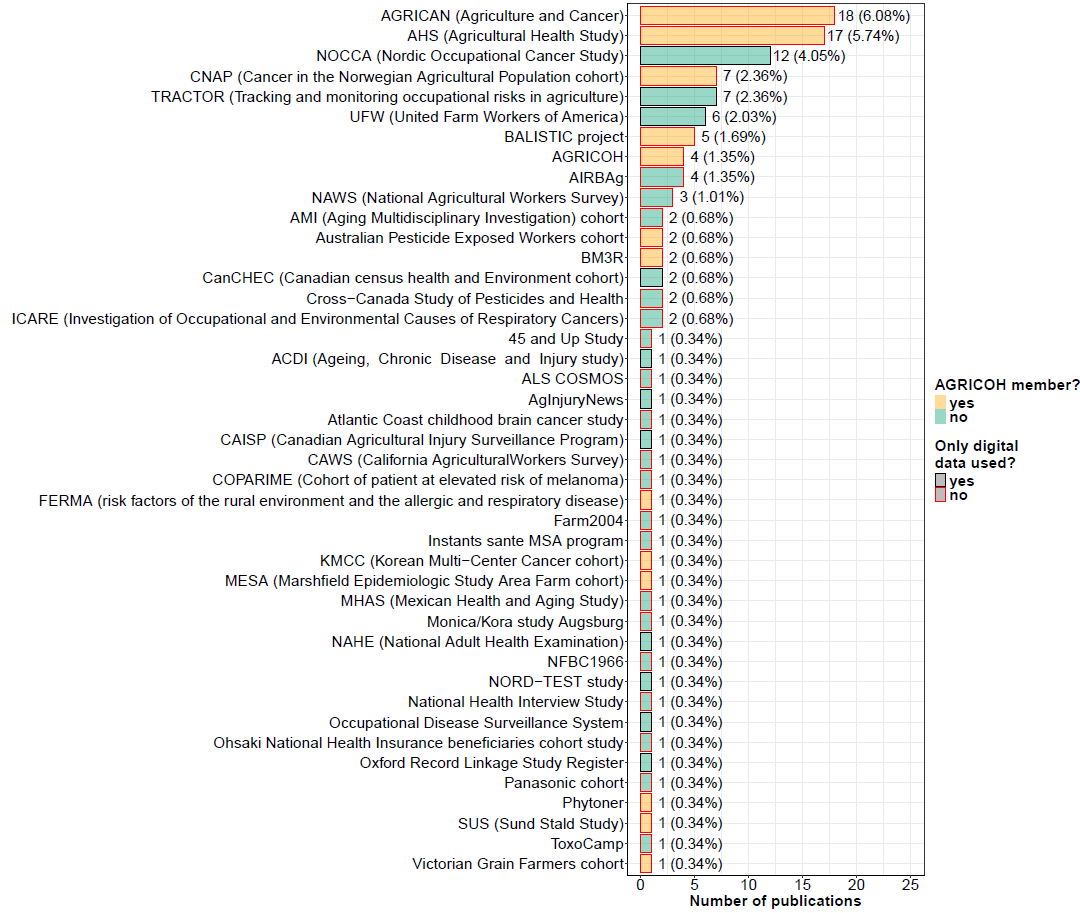


# **Figure S8**. Type of digital data used.


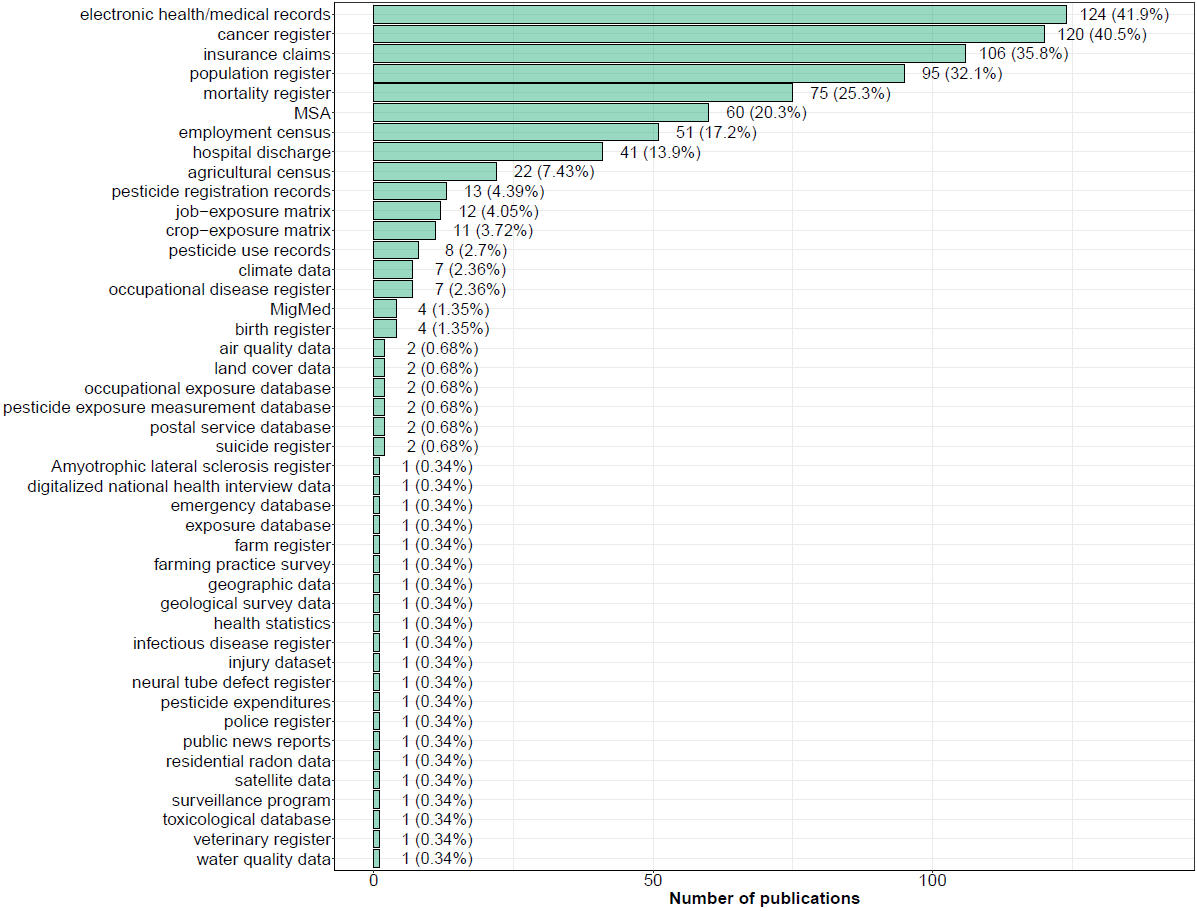


# **Figure S9**. Active data used.


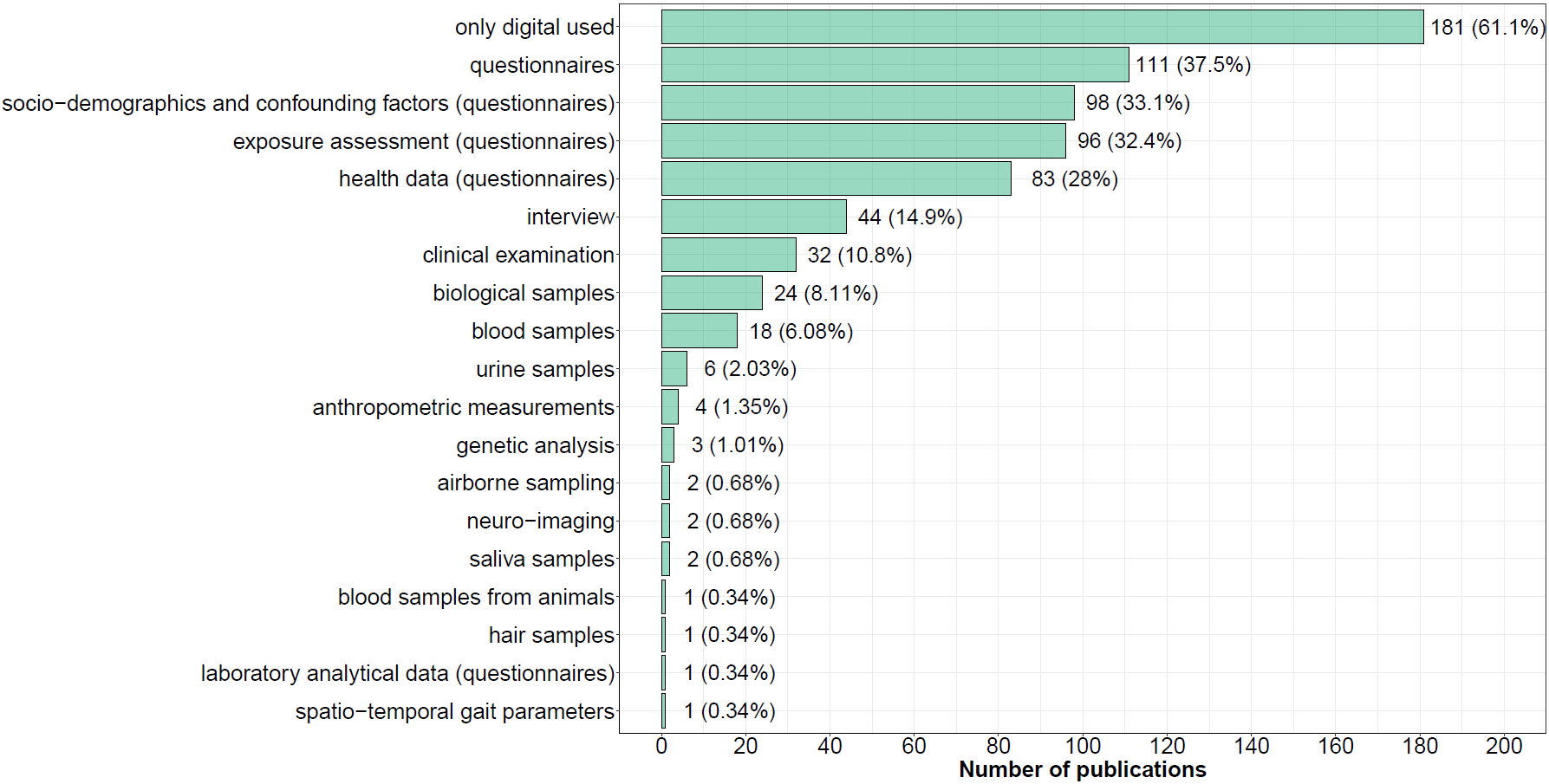


# **Figure S10**. Most frequent farming exposure proxy.


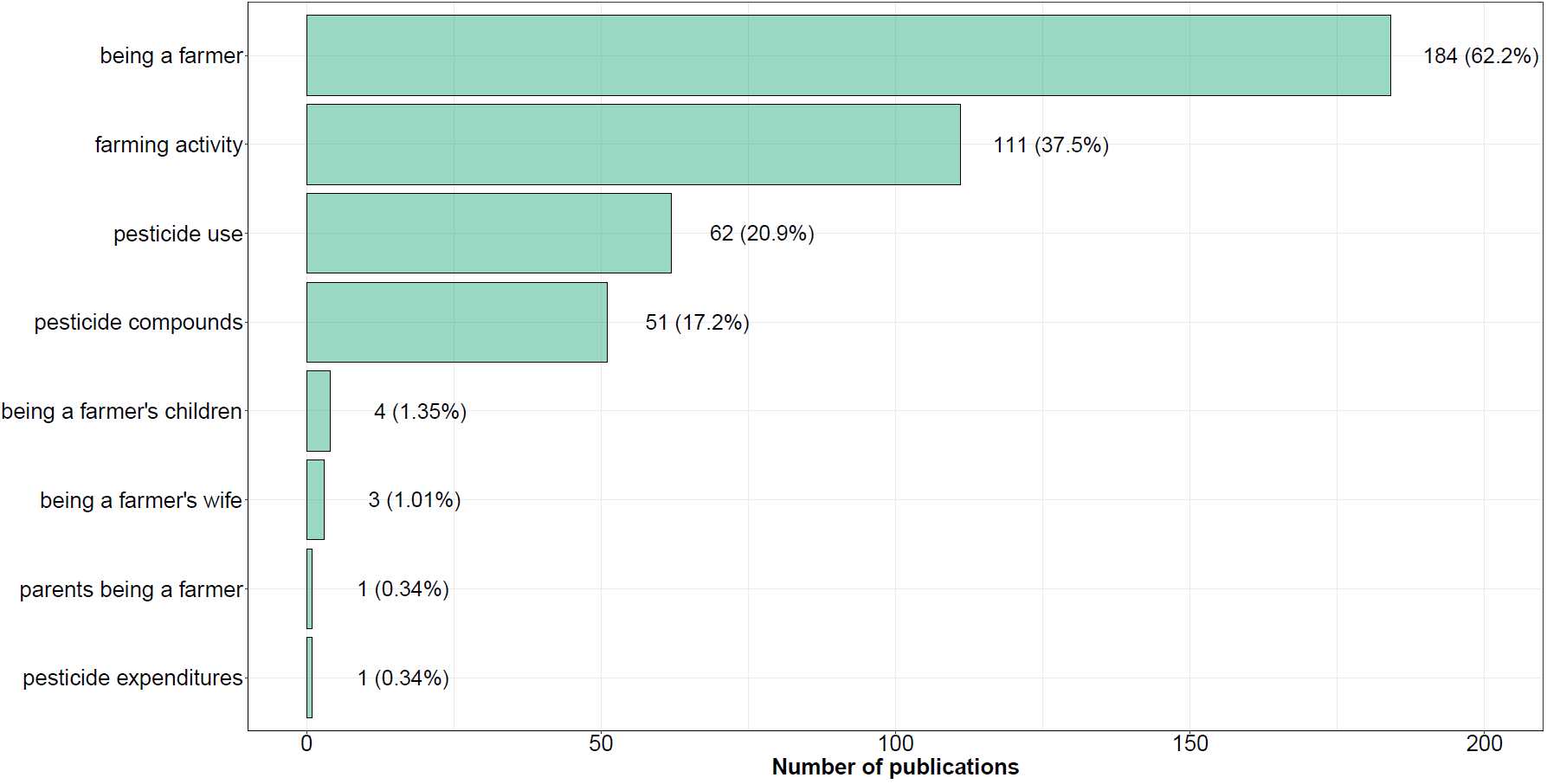


# **Figure S11**. Most frequent health events studied.


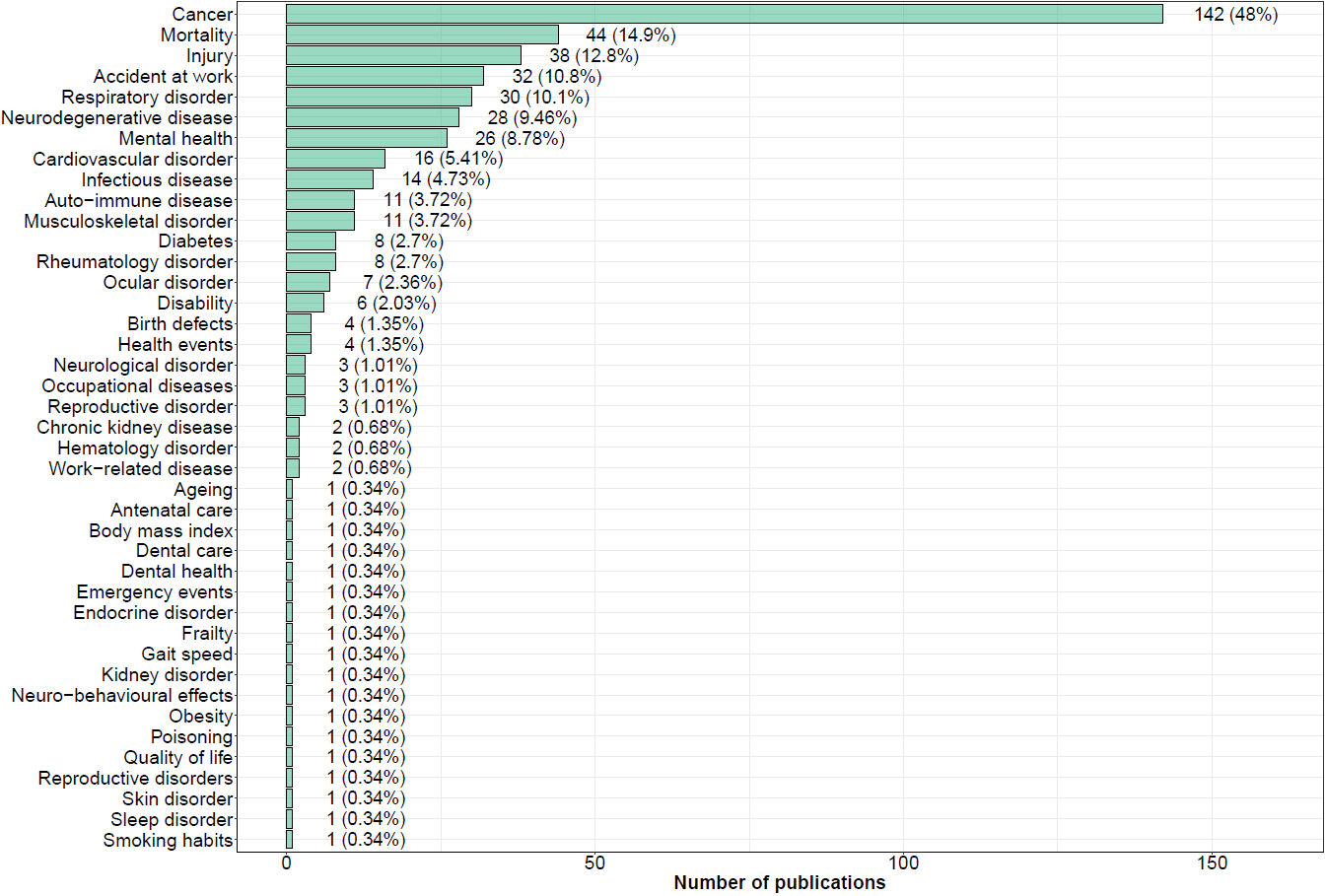


# **Figure S12**. Most frequent cancer studied.


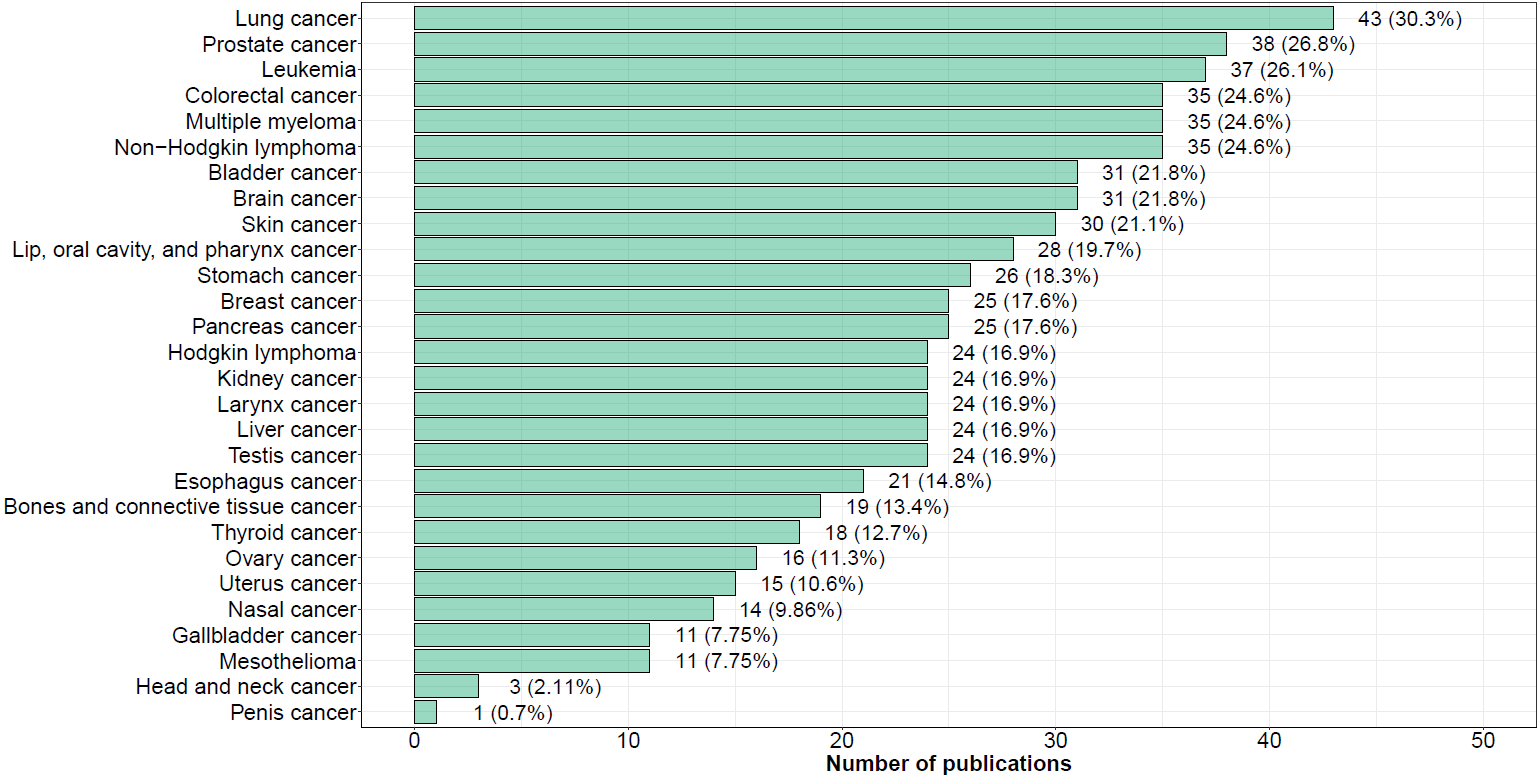


# **Figure S13**. Most frequent neurodegenerative disease studied.


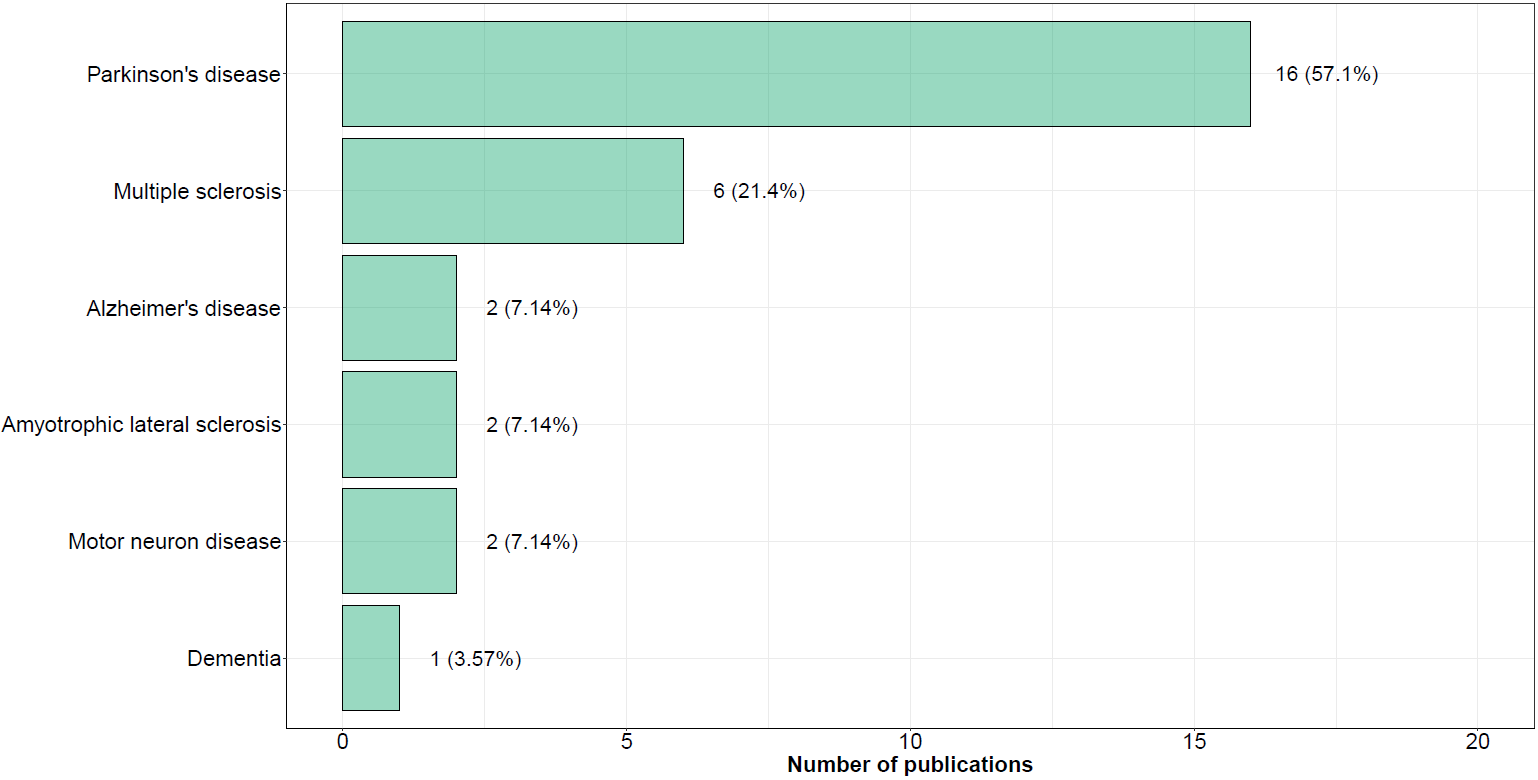


# **Figure S14**. Most frequent mental health issue studied.


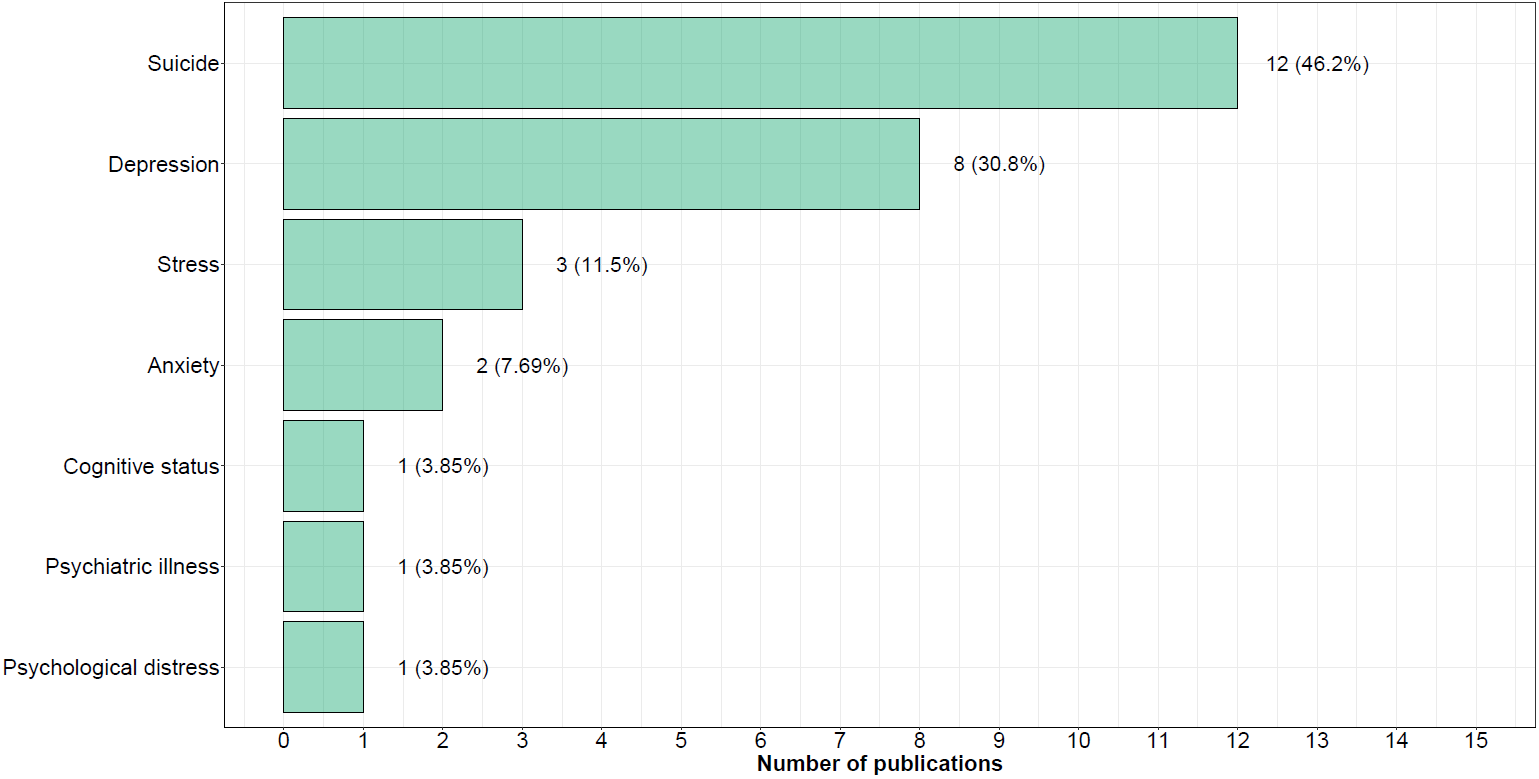


# **Table S7**. Top 50 of the most frequent keywords.

| **Keyword** | **First appearance** | **Last appearance** | **Number of publications** | **Total citation** | **Local citation** | **Average citation per year** | **Mean AGR** | **h-index** | **g-index** | **m-index** |
| --- | --- | --- | --- | --- | --- | --- | --- | --- | --- | --- |
| Cancer | 1975 | 2024 | 150 (50.7%) | 5766 | 137 (2.74%) | 115 | 13.5 | 43 | 70 | 0.860 |
| Mortality | 1981 | 2024 | 96 (32.4%) | 3097 | 75 (2.81%) | 70.4 | 8.15 | 34 | 53 | 0.773 |
| Pesticide | 1981 | 2024 | 88 (29.7%) | 3569 | 81 (2.44%) | 81.1 | -7.46 | 36 | 58 | 0.818 |
| Occupation | 1980 | 2024 | 82 (27.7%) | 2959 | 55 (2.33%) | 65.8 | 14.2 | 31 | 53 | 0.689 |
| Farmer | 1981 | 2024 | 77 (26.0%) | 2856 | 91 (3.25%) | 64.9 | 6.36 | 34 | 52 | 0.773 |
| Agriculture | 1980 | 2024 | 74 (25.0%) | 1701 | 55 (3.85%) | 37.8 | -9.96 | 26 | 39 | 0.578 |
| Epidemiology | 1991 | 2024 | 57 (19.3%) | 1396 | 17 (1.47%) | 41.1 | 23.1 | 21 | 37 | 0.618 |
| Exposure | 1992 | 2024 | 57 (19.3%) | 1713 | 27 (1.76%) | 61.2 | -2.73 | 21 | 41 | 0.636 |
| Lymphohematopoietic cancer | 1981 | 2023 | 54 (18.2%) | 2906 | 66 (2.69%) | 67.6 | -13.8 | 31 | 53 | 0.705 |
| Respiratory cancer | 1987 | 2024 | 51 (17.2%) | 2566 | 67 (3.29%) | 67.5 | -16.1 | 28 | 50 | 0.737 |
| Risk | 1992 | 2024 | 51 (17.2%) | 1909 | 27 (1.53%) | 57.8 | 6.01 | 25 | 43 | 0.758 |
| Male genital organ cancer | 1987 | 2024 | 47 (15.9%) | 2865 | 75 (3.12%) | 75.4 | -12.5 | 29 | 47 | 0.763 |
| Risk factor | 1997 | 2024 | 44 (14.9%) | 818 | 17 (2.08%) | 29.2 | -19.0 | 17 | 27 | 0.607 |
| Digestive organ cancer | 1987 | 2023 | 41 (13.9%) | 2331 | 58 (3.09%) | 63.0 | -11.3 | 26 | 41 | 0.684 |
| Occupational exposure | 1992 | 2024 | 41 (13.9%) | 789 | 39 (4.94%) | 23.9 | 2.01 | 17 | 27 | 0.515 |
| Worker | 1992 | 2024 | 41 (13.9%) | 1243 | 32 (2.81%) | 37.7 | -4.85 | 19 | 35 | 0.576 |
| Injury | 1991 | 2024 | 40 (13.5%) | 734 | 18 (2.69%) | 21.6 | -5.44 | 15 | 26 | 0.441 |
| Cohort | 1992 | 2024 | 39 (13.2%) | 879 | 40 (4.55%) | 26.6 | 1.41 | 16 | 29 | 0.485 |
| Incidence | 1985 | 2023 | 34 (11.5%) | 1187 | 29 (3.21%) | 30.4 | -13.7 | 20 | 34 | 0.500 |
| Neurodegenerative disease | 2000 | 2024 | 33 (11.1%) | 1216 | 13 (1.12%) | 55.3 | -10.0 | 17 | 33 | 0.680 |
| Urinary tract cancer | 1987 | 2023 | 33 (11.1%) | 1975 | 47 (3.03%) | 53.4 | -9.59 | 21 | 33 | 0.553 |
| Workplace accident | 1987 | 2024 | 32 (10.8%) | 727 | 17 (2.76%) | 20.8 | -6.58 | 15 | 26 | 0.395 |
| Respiratory disorder | 1986 | 2024 | 32 (10.8%) | 901 | 21 (2.46%) | 23.1 | -15.2 | 15 | 30 | 0.385 |
| US | 1994 | 2024 | 32 (10.8%) | 1167 | 14 (1.26%) | 40.2 | -25.0 | 18 | 32 | 0.581 |
| Brain cancer | 1981 | 2024 | 31 (10.5%) | 1858 | 61 (3.58%) | 42.2 | -10.6 | 23 | 31 | 0.523 |
| Health | 1997 | 2023 | 31 (10.5%) | 463 | 25 (5.45%) | 17.1 | -12.0 | 12 | 21 | 0.429 |
| Lip, oral cavity, and pharynx cancer | 1975 | 2024 | 30 (10.1%) | 1827 | 55 (3.93%) | 36.5 | -14.8 | 21 | 30 | 0.420 |
| Male | 1985 | 2023 | 30 (10.1%) | 1150 | 29 (2.86%) | 29.5 | -15.4 | 19 | 30 | 0.475 |
| Skin cancer | 1975 | 2023 | 30 (10.1%) | 1999 | 44 (2.80%) | 40.8 | -22.8 | 22 | 30 | 0.440 |
| Prevalence | 1998 | 2024 | 28 (9.46%) | 438 | 17 (3.88%) | 16.2 | -14.8 | 12 | 20 | 0.444 |
| Socio-economic status | 1980 | 2024 | 27 (9.12%) | 685 | 7 (1.33%) | 15.2 | -24.4 | 16 | 26 | 0.356 |
| Animal farming | 1975 | 2023 | 26 (8.78%) | 883 | 29 (3.28%) | 18.0 | -20.1 | 15 | 26 | 0.300 |
| Breast cancer | 1987 | 2024 | 26 (8.78%) | 1628 | 48 (3.63%) | 42.8 | -18.7 | 20 | 26 | 0.526 |
| Farming activity | 1985 | 2023 | 25 (8.45%) | 925 | 35 (3.82%) | 23.7 | -10.7 | 14 | 25 | 0.350 |
| Mental health | 1991 | 2024 | 25 (8.45%) | 512 | 6 (1.22%) | 15.1 | -28.4 | 12 | 22 | 0.353 |
| Asthma | 1986 | 2024 | 24 (8.11%) | 743 | 5 (0.72%) | 19.1 | -12.4 | 13 | 24 | 0.333 |
| Farm | 1992 | 2024 | 24 (8.11%) | 516 | 8 (1.75%) | 15.6 | -6.57 | 10 | 22 | 0.303 |
| Herbicide | 1984 | 2024 | 22 (7.43%) | 1349 | 26 (1.93%) | 32.9 | -12.6 | 15 | 22 | 0.366 |
| Mesenchymal cancer | 1984 | 2023 | 20 (6.76%) | 1466 | 35 (3.07%) | 36.6 | -18.1 | 14 | 20 | 0.341 |
| COPD | 1987 | 2024 | 19 (6.42%) | 298 | 14 (5.60%) | 11.0 | -1.32 | 8 | 17 | 0.211 |
| Environmental exposure | 1999 | 2024 | 19 (6.42%) | 688 | 12 (3.20%) | 31.3 | -17.9 | 11 | 19 | 0.423 |
| Female | 1980 | 2024 | 19 (6.42%) | 454 | 7 (1.54%) | 10.1 | -25.6 | 11 | 19 | 0.244 |
| Female genital organ cancer | 1987 | 2024 | 19 (6.42%) | 1083 | 47 (4.34%) | 28.5 | -21.1 | 14 | 19 | 0.368 |
| Insecticide | 1992 | 2023 | 19 (6.42%) | 1218 | 33 (2.71%) | 38.1 | -21.9 | 14 | 19 | 0.424 |
| Parkinson's disease | 2000 | 2021 | 19 (6.42%) | 972 | 12 (1.32%) | 51.2 | -19.7 | 16 | 19 | 0.640 |
| Pesticide applicator | 1987 | 2024 | 19 (6.42%) | 984 | 32 (3.57%) | 25.9 | -30.7 | 14 | 19 | 0.368 |
| Smoking | 1989 | 2022 | 19 (6.42%) | 586 | 14 (2.66%) | 17.2 | -18.6 | 12 | 19 | 0.333 |
| Assessment | 2003 | 2024 | 18 (6.08%) | 293 | 0 (0.00%) | 13.3 | -18.9 | 10 | 17 | 0.455 |
| CVD | 1987 | 2023 | 17 (5.74%) | 623 | 3 (0.52%) | 18.3 | -18.9 | 11 | 17 | 0.289 |
| Disease | 1997 | 2023 | 17 (5.74%) | 385 | 2 (0.58%) | 14.3 | -11.1 | 8 | 17 | 0.286 |
| Follow-up | 1997 | 2024 | 17 (5.74%) | 562 | 5 (0.96%) | 20.1 | -23.8 | 10 | 17 | 0.357 |
| Surveillance | 1988 | 2024 | 17 (5.74%) | 291 | 13 (5.04%) | 7.86 | -17.6 | 8 | 17 | 0.216 |

*Note*: AGR: annual growth rate, COPD: chronic obstructive pulmonary disease, CVD: cardiovascular disorder. Please refer to the beginning of the supplemental materials for the definition of the bibliometric indices.

# **Figure S15**. Top 5 of the most frequent keywords – growth trends.


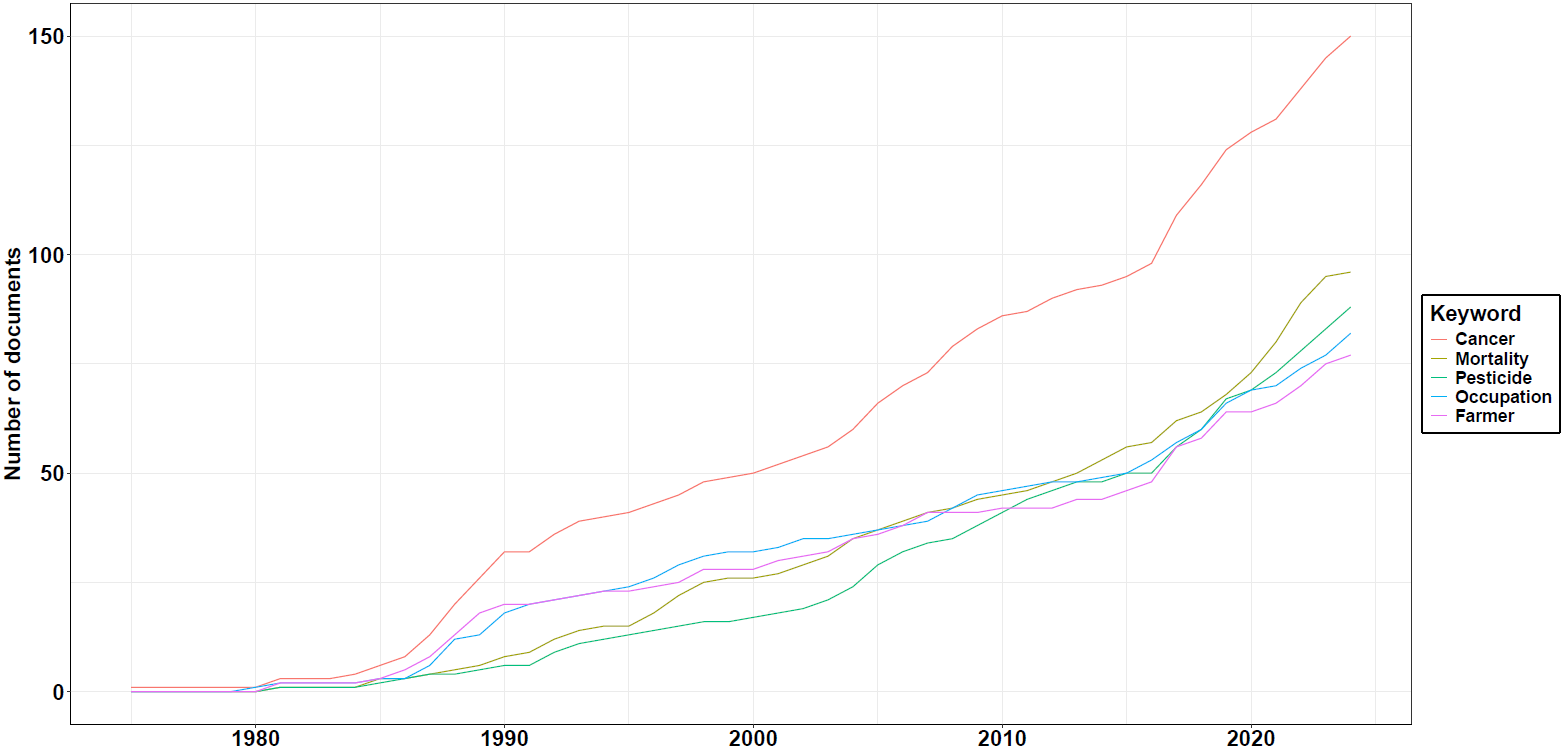


# **Figure S16**. Top ten of the most popular keywords by total number of years of appearance.

AAW: workplace accident; ALS: amyotrophic lateral sclerosis, BMI: body mass index, BP: blood pressure, COPD: chronic obstructive pulmonary disease, CVD: cardiovascular disorder, EMR: electronic medical record, IgE: immunoglobulin E, MSD: musculoskeletal disorder, SES: socio-economic status, US: United States of America.


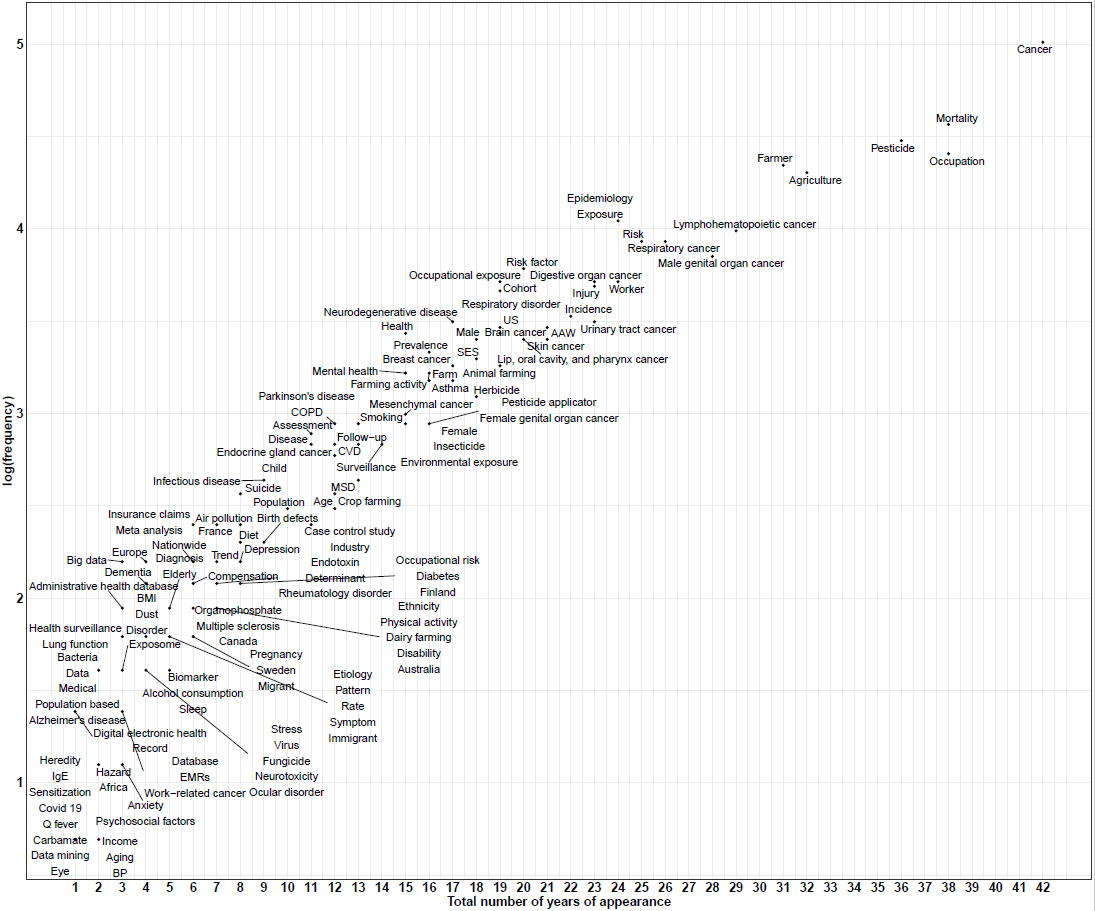


# **Figure S17**. Top 10 of the most frequent keywords by time period.

BMI: body mass index, EMR: electronic medical record, ETS: environmental tobacco smoking.


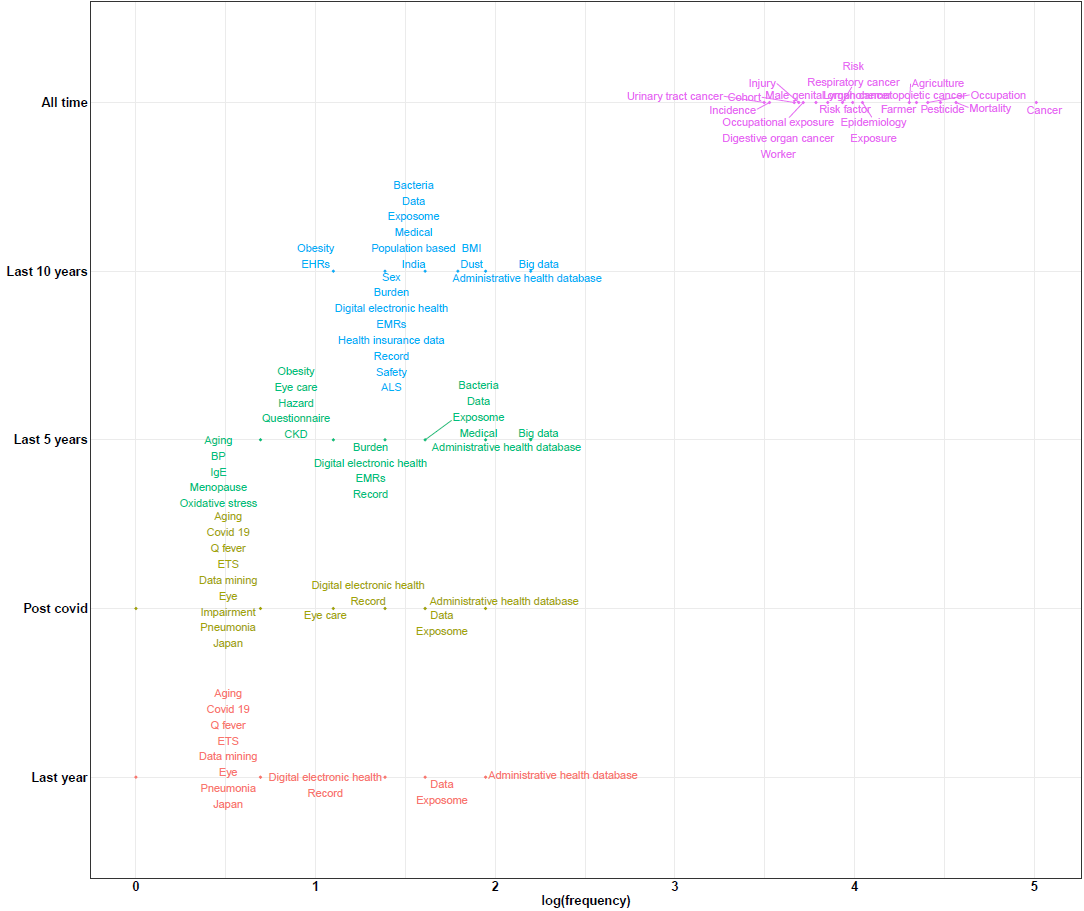


# **Multimedia appendix 3**. Co-occurrence between exposome-related keywords and health event-related keywords.

Please refer to the MS Excel file entitled “Multimedia appendix 3”.

# **Figure S18**. Keyword co-occurrence network between potential risk factor and mental health disorder keywords.

BMI: body mass index, SES: socio-economic status. Larger the node size, higher the number of co-occurrence. Red nodes refer to health events while blue nodes refer to potential risk factors.


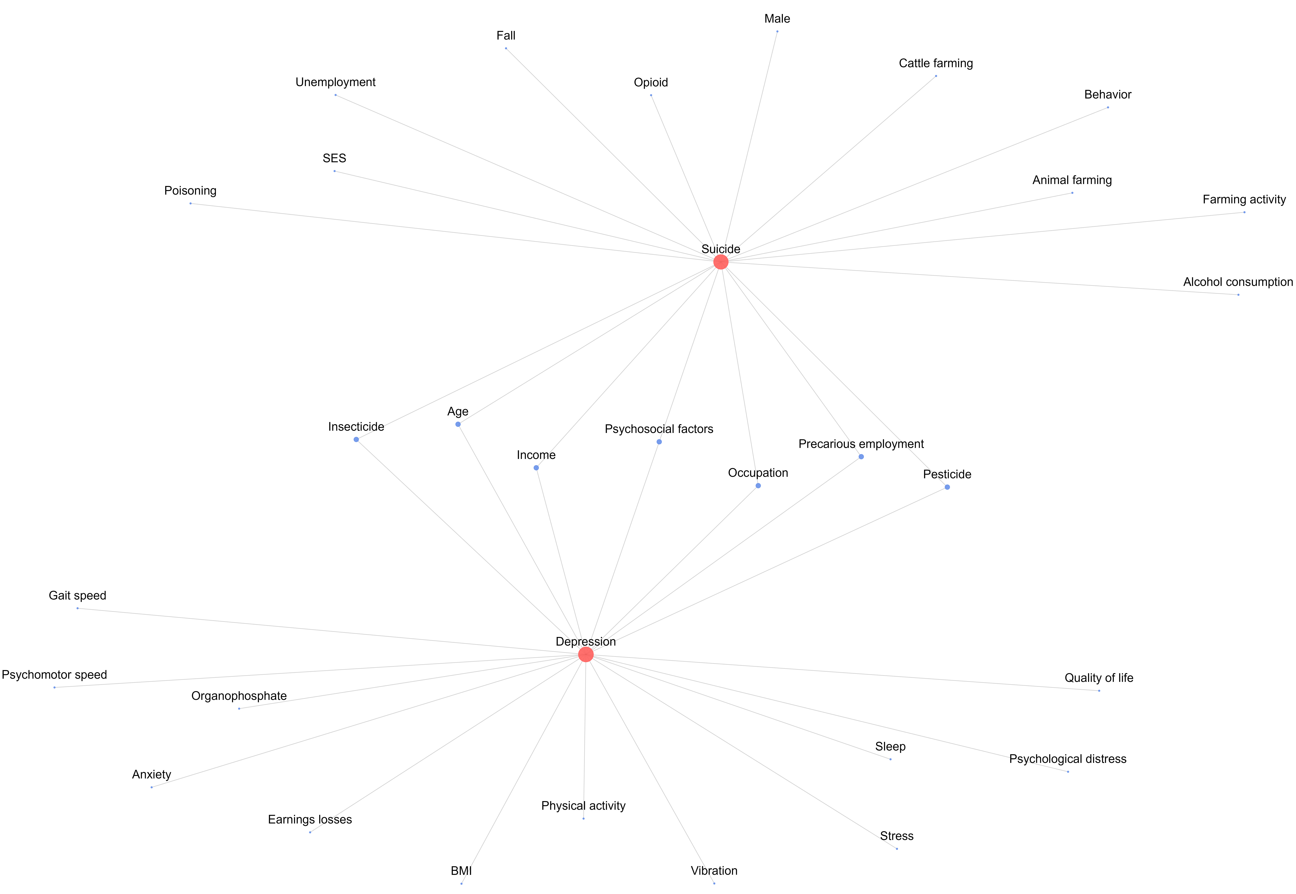


# **Figure S19**. Chord diagram of keyword co-occurrence between potential risk factor and neurodegenerative disease keywords.

ALS: amyotrophic lateral sclerosis, SES: socio-economic status, VOC: volatile organic compound.


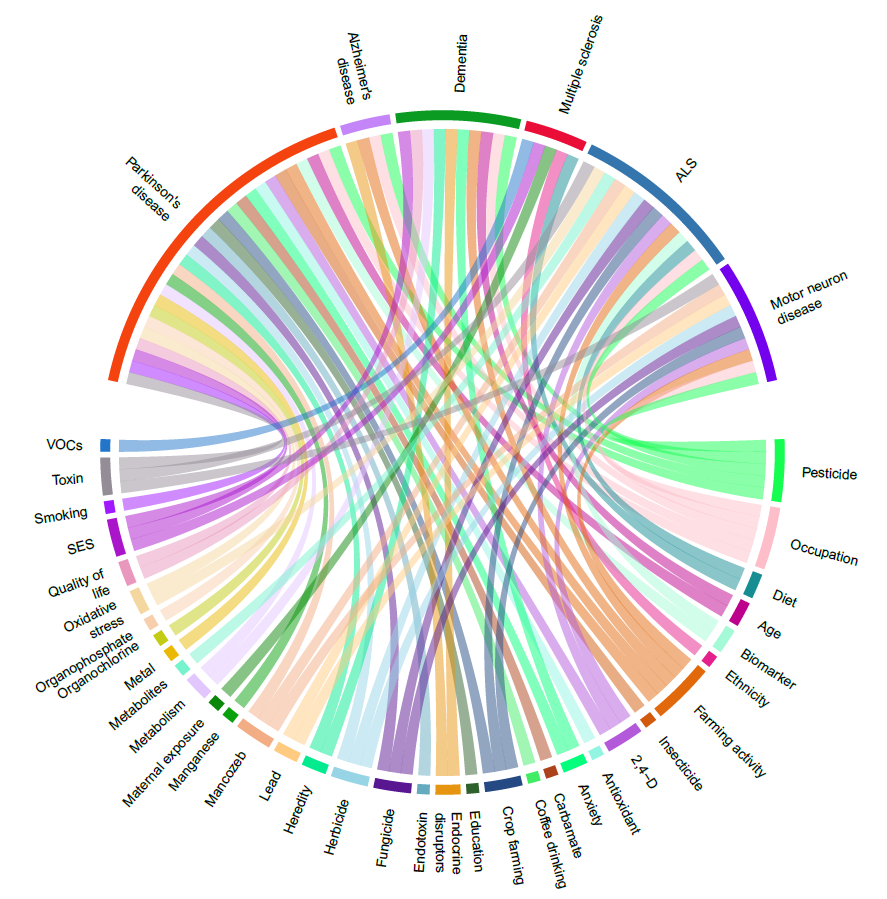


# **Figure S20**. Keyword co-occurrence network between potential risk factor and neurodegenerative disease keywords.

ALS: amyotrophic lateral sclerosis, SES: socio-economic status, VOC: volatile organic compound. Larger the node size, higher the number of co-occurrence. Red nodes refer to health events while blue nodes refer to potential risk factors.


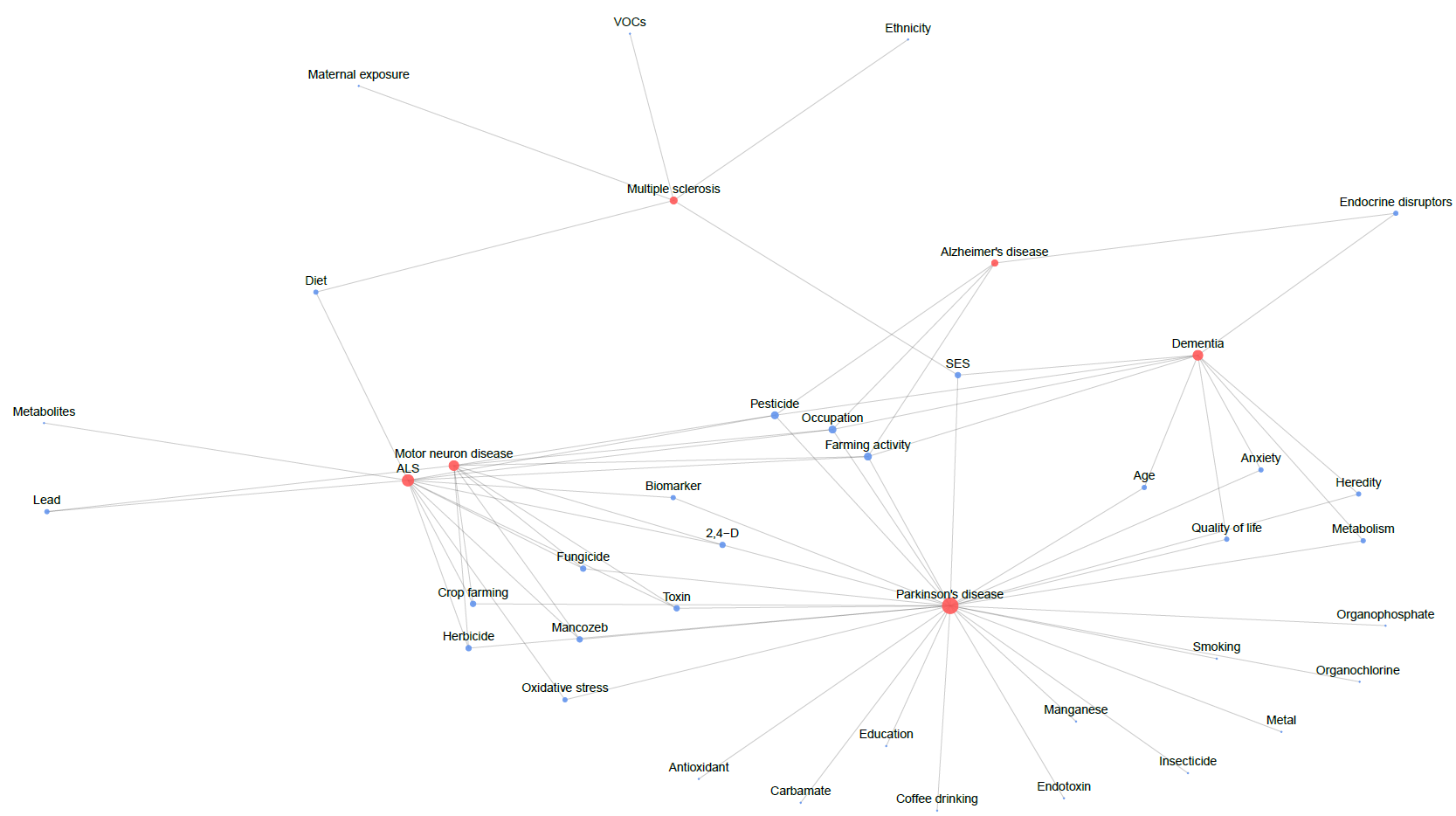


# **Figure S21**. Chord diagram of keyword co-occurrence between potential risk factor and autoimmune disease keywords.

BMI: body mass index, BP: blood pressure, IBD: inflammatory bowel disease, SES: socio-economic status, VOC: volatile organic compound.


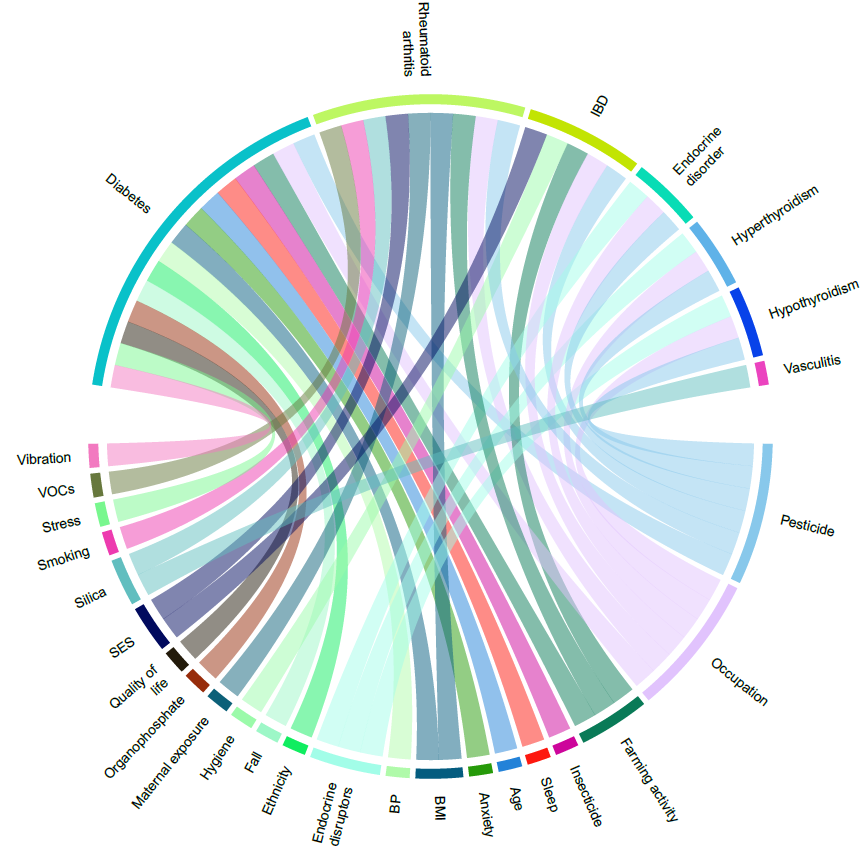


# **Figure S22**. Keyword co-occurrence network between potential risk factor and autoimmune disease keywords.

BMI: body mass index, BP: blood pressure, IBD: inflammatory bowel disease, SES: socio-economic status, VOC: volatile organic compound. Larger the node size, higher the number of co-occurrence. Red nodes refer to health events while blue nodes refer to potential risk factors.


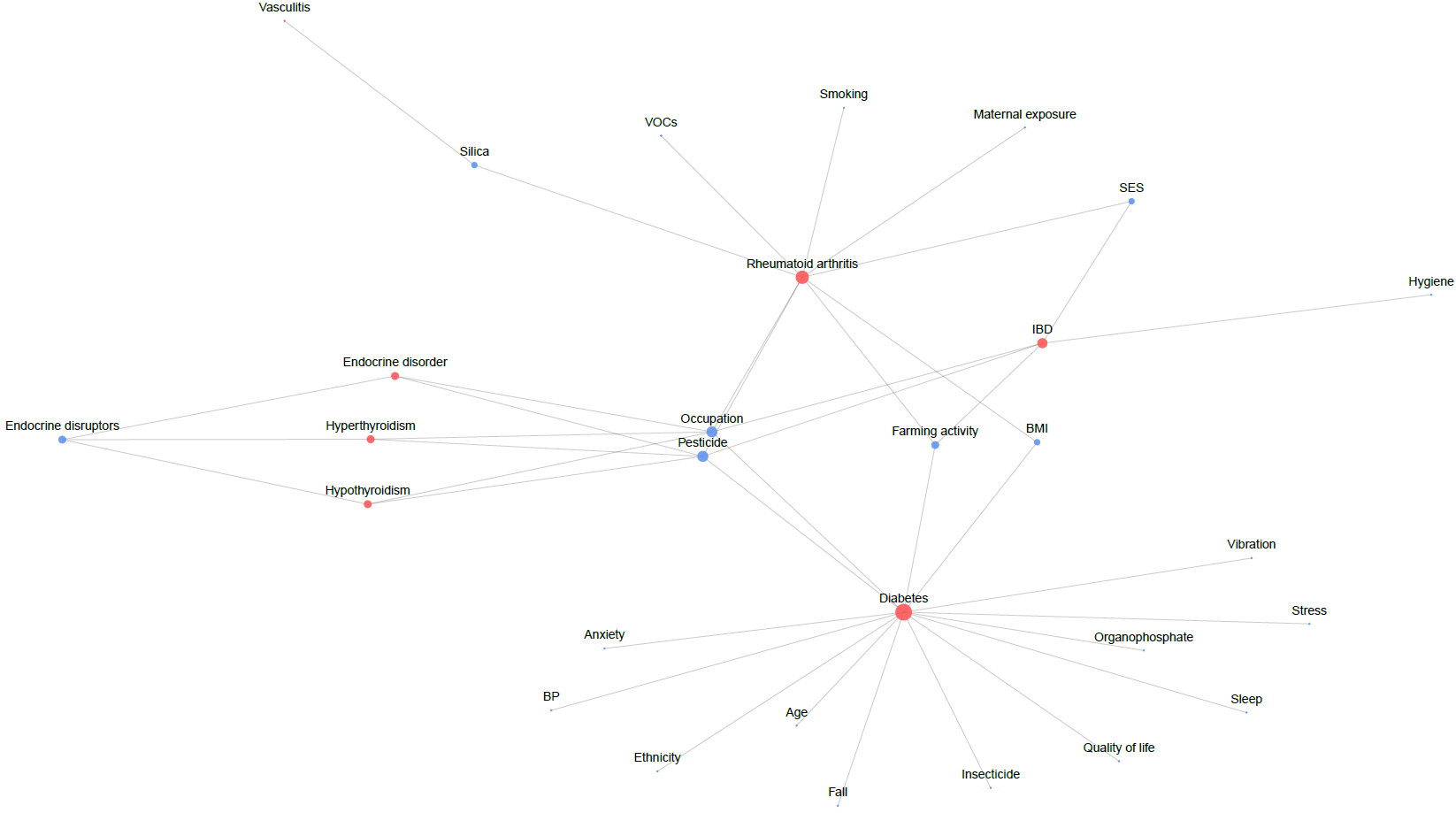


# **Figure S23**. Chord diagram of keyword co-occurrence between potential risk factor and breast and genital organ cancer keywords.

2,4-D: 2,4-dichlorophenoxyacetic acid, BMI: body mass index, DDT: dichlorodiphenyltrichloroethane, EMF: electromagnetic field, PAH: polycyclic aromatic hydrocarbon, PCB: polychlorinated biphenyls, SES: socio-economic status.


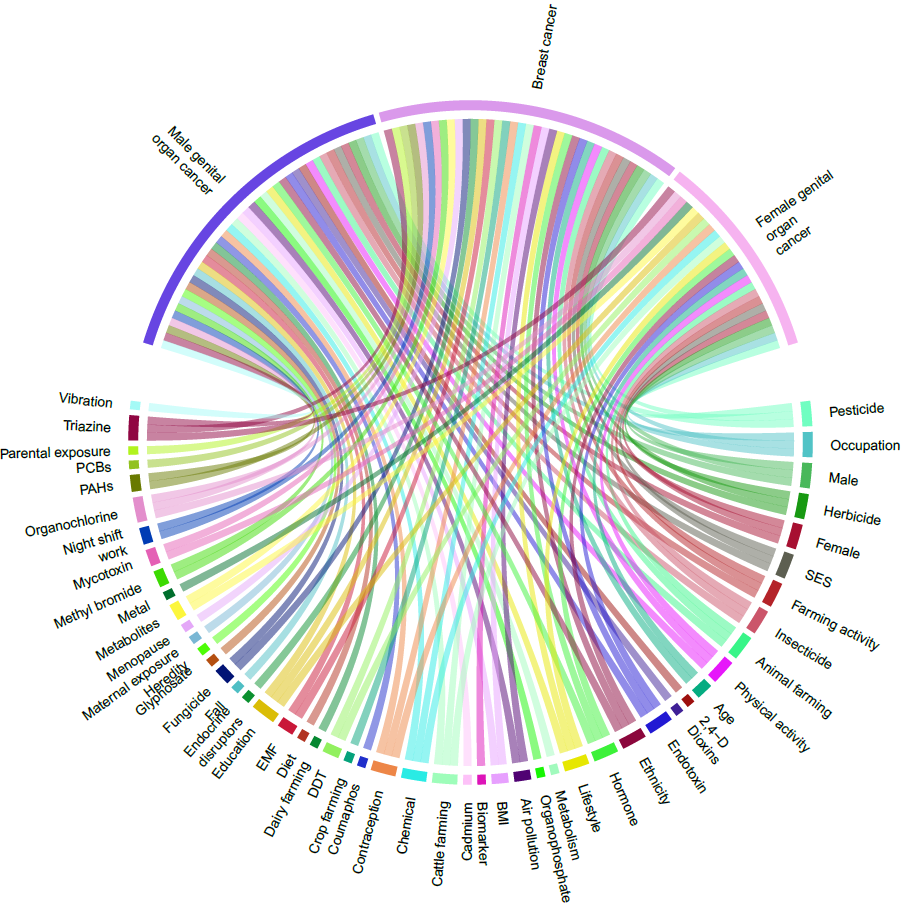


# **Figure S24**. Keyword co-occurrence network between potential risk factor and breast and genital organ cancer keywords.

2,4-D: 2,4-dichlorophenoxyacetic acid, BMI: body mass index, DDT: dichlorodiphenyltrichloroethane, EMF: electromagnetic field, PAH: polycyclic aromatic hydrocarbon, PCB: polychlorinated biphenyls, SES: socio-economic status. Larger the node size, higher the number of co-occurrence. Red nodes refer to health events while blue nodes refer to potential risk factors.


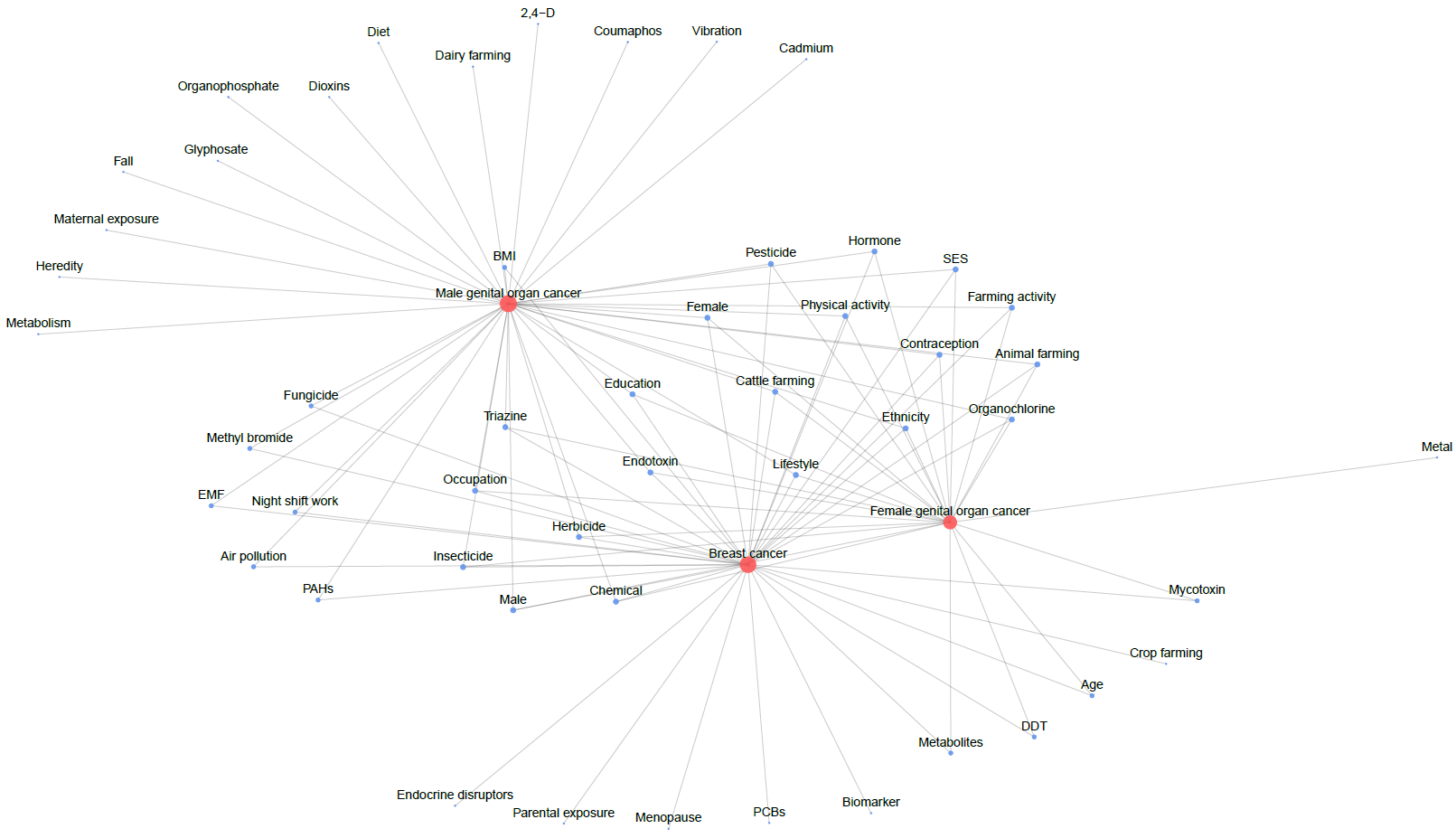


# **Figure S25**. Chord diagram of keyword co-occurrence between potential risk factor and digestive organ, lip, oral cavity and pharynx cancer keywords.

BMI: body mass index, DDT: dichlorodiphenyltrichloroethane, EMF: electromagnetic field, ETS: environmental tobacco smoking, PAH: polycyclic aromatic hydrocarbon, SES: socio-economic status, VOC: volatile organic compound, UV: ultraviolet.


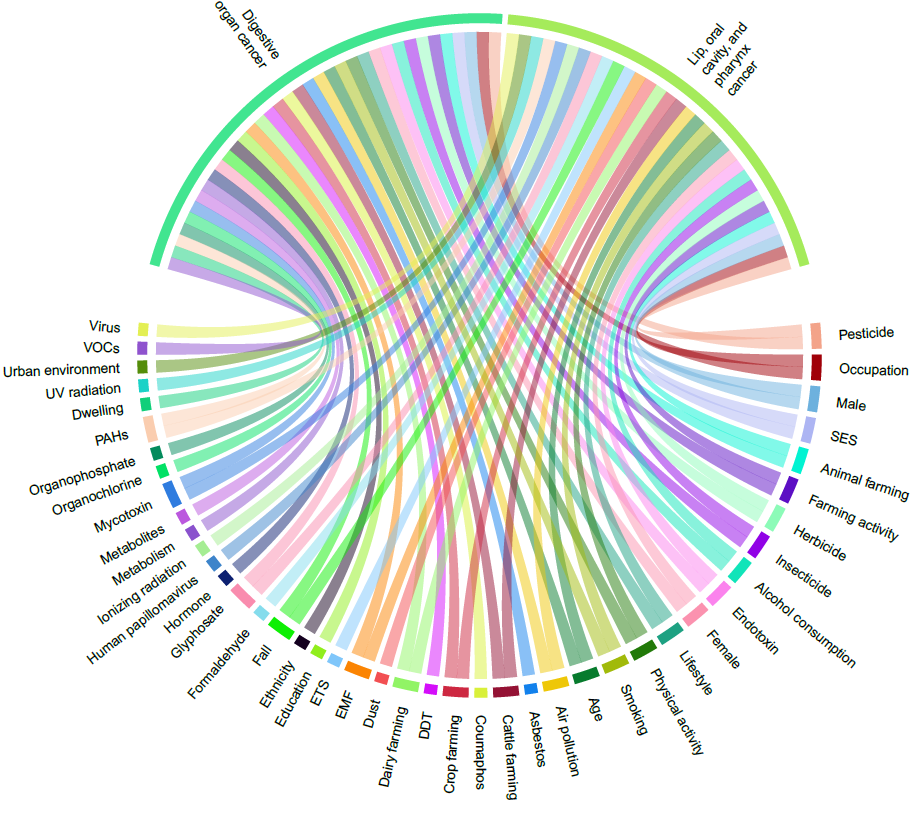


# **Figure S26**. Keyword co-occurrence network between potential risk factor and digestive organ, lip, oral cavity and pharynx cancer keywords.

BMI: body mass index, DDT: dichlorodiphenyltrichloroethane, EMF: electromagnetic field, ETS: environmental tobacco smoking, PAH: polycyclic aromatic hydrocarbon, SES: socio-economic status, VOC: volatile organic compound, UV: ultraviolet. Larger the node size, higher the number of co-occurrence. Red nodes refer to health events while blue nodes refer to potential risk factors.


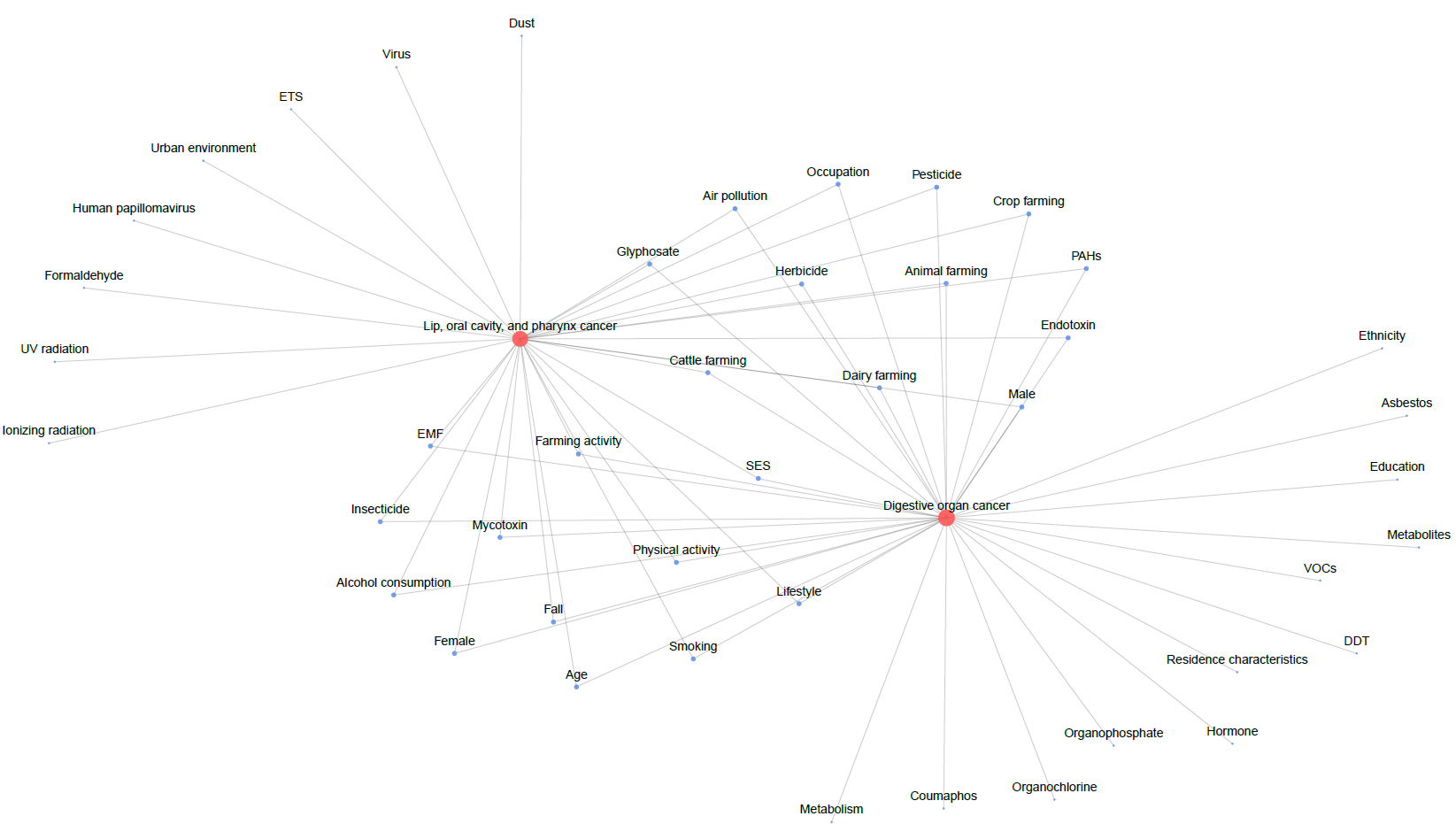


# **Figure S27**. Chord diagram of keyword co-occurrence between potential risk factor and lymphohematopoietic and mesenchymal cancer keywords.

2,4-D: 2,4-dichlorophenoxyacetic acid, BMI: body mass index, DDT: dichlorodiphenyltrichloroethane, SES: socio-economic status, VOC: volatile organic compound.


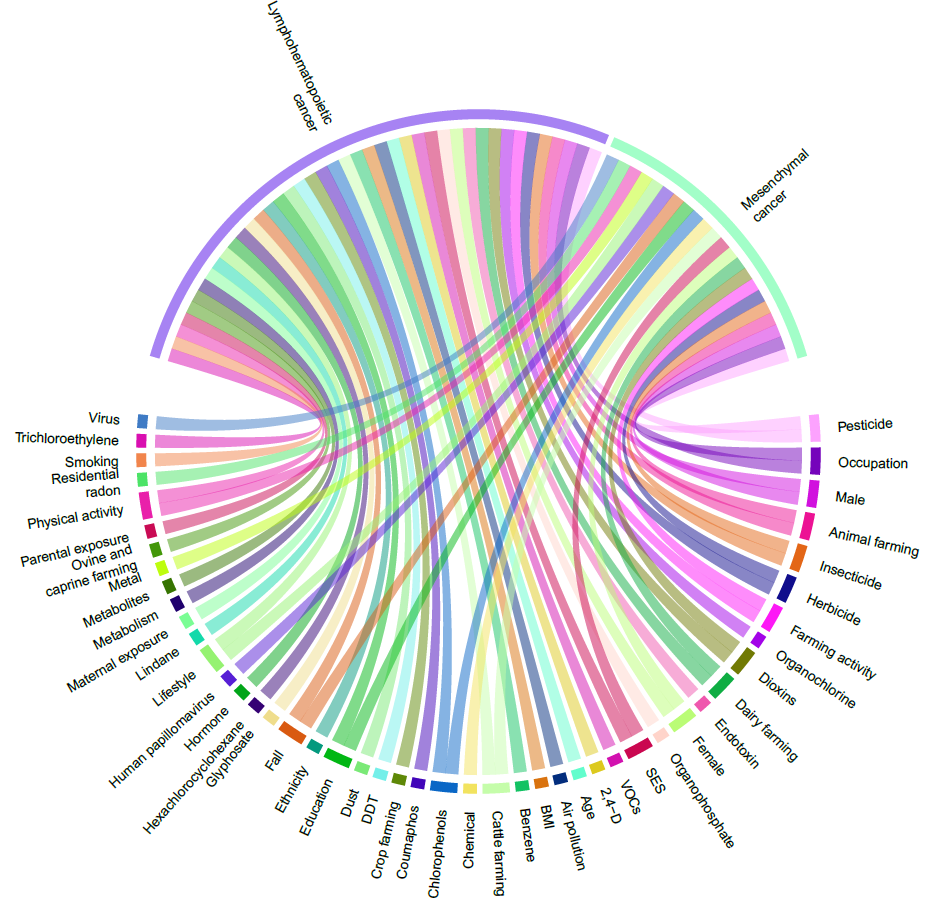


# **Figure S28**. Keyword co-occurrence network between potential risk factor and lymphohematopoietic and mesenchymal cancer keywords.

2,4-D: 2,4-dichlorophenoxyacetic acid, BMI: body mass index, DDT: dichlorodiphenyltrichloroethane, SES: socio-economic status, VOC: volatile organic compound. Larger the node size, higher the number of co-occurrence. Red nodes refer to health events while blue nodes refer to potential risk factors.


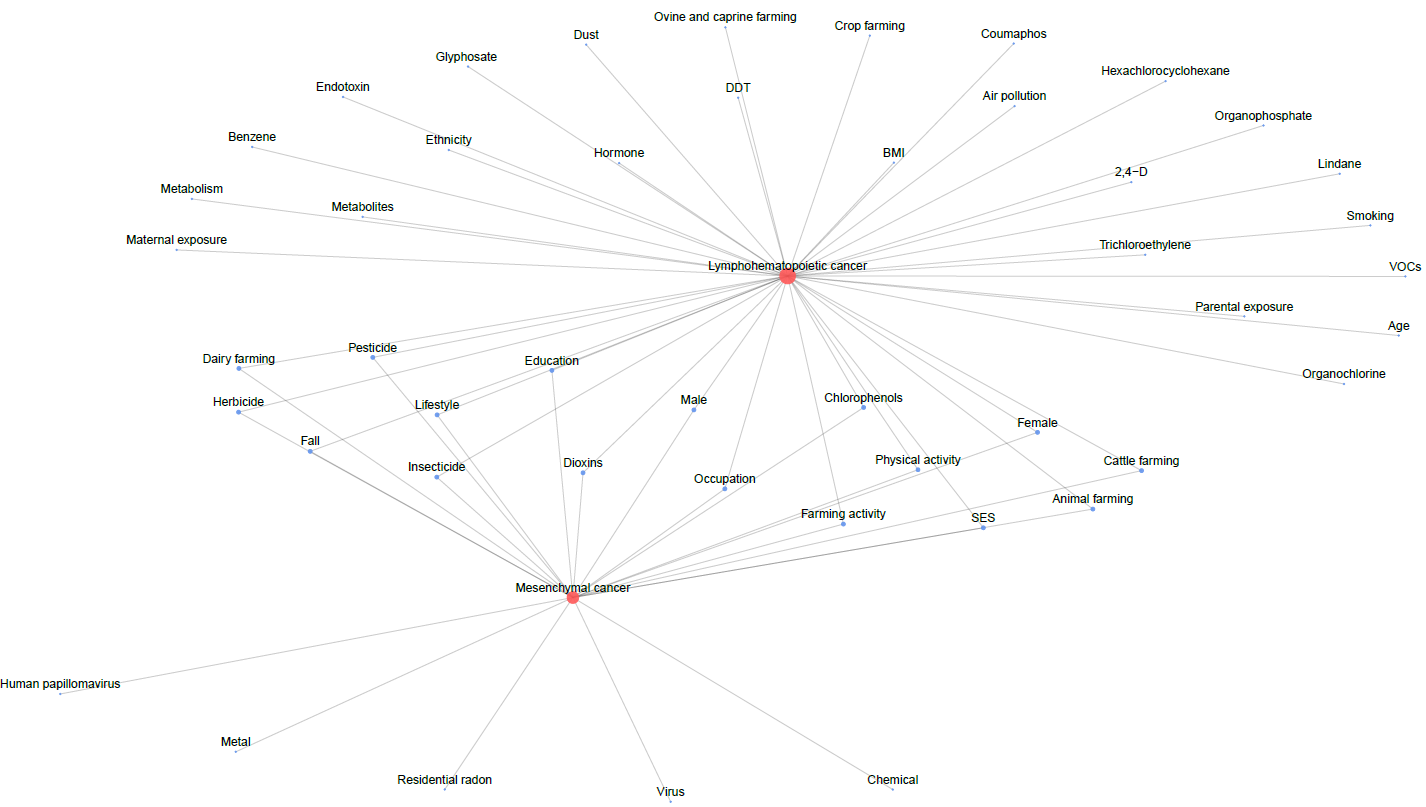


# **Figure S29**. Chord diagram of keyword co-occurrence between potential risk factor and brain, skin, ocular and endocrine gland cancer keywords.

DDT: dichlorodiphenyltrichloroethane, EMF: electromagnetic field, PAH: polycyclic aromatic hydrocarbon, SES: socio-economic status, UV: ultraviolet.


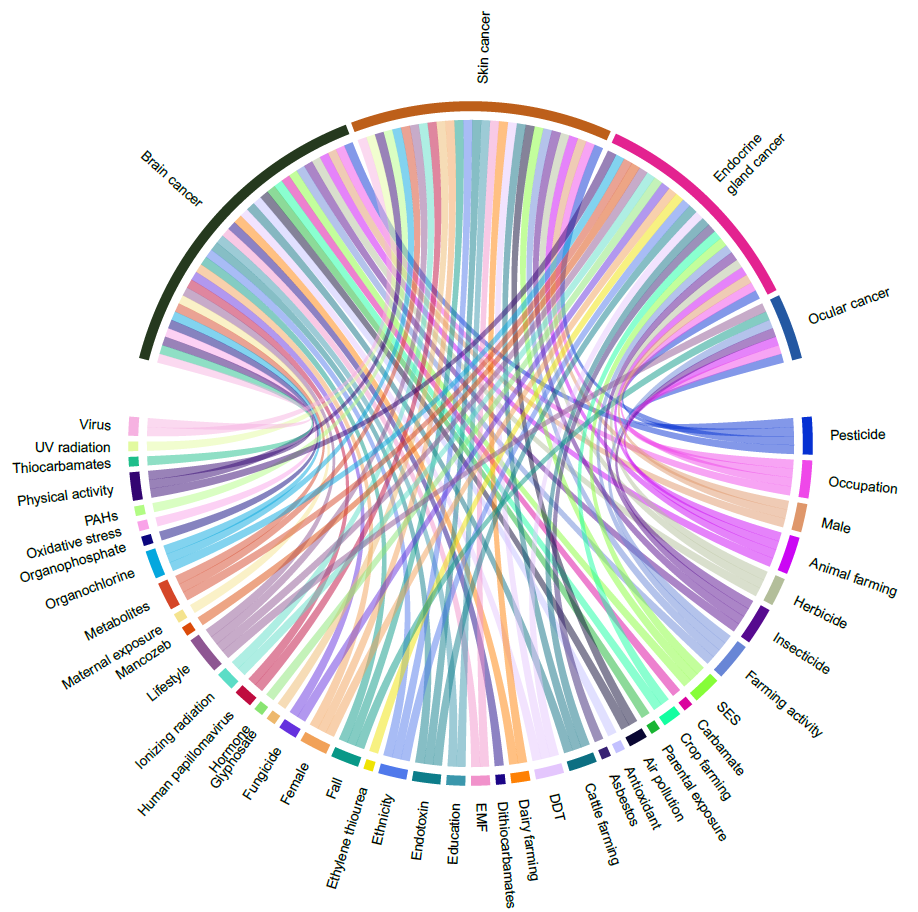


# **Figure S30**. Keyword co-occurrence network between potential risk factor and brain, skin, ocular and endocrine gland cancer keywords.

DDT: dichlorodiphenyltrichloroethane, EMF: electromagnetic field, PAH: polycyclic aromatic hydrocarbon, SES: socio-economic status, UV: ultraviolet. Red nodes refer to health events while blue nodes refer to potential risk factors.


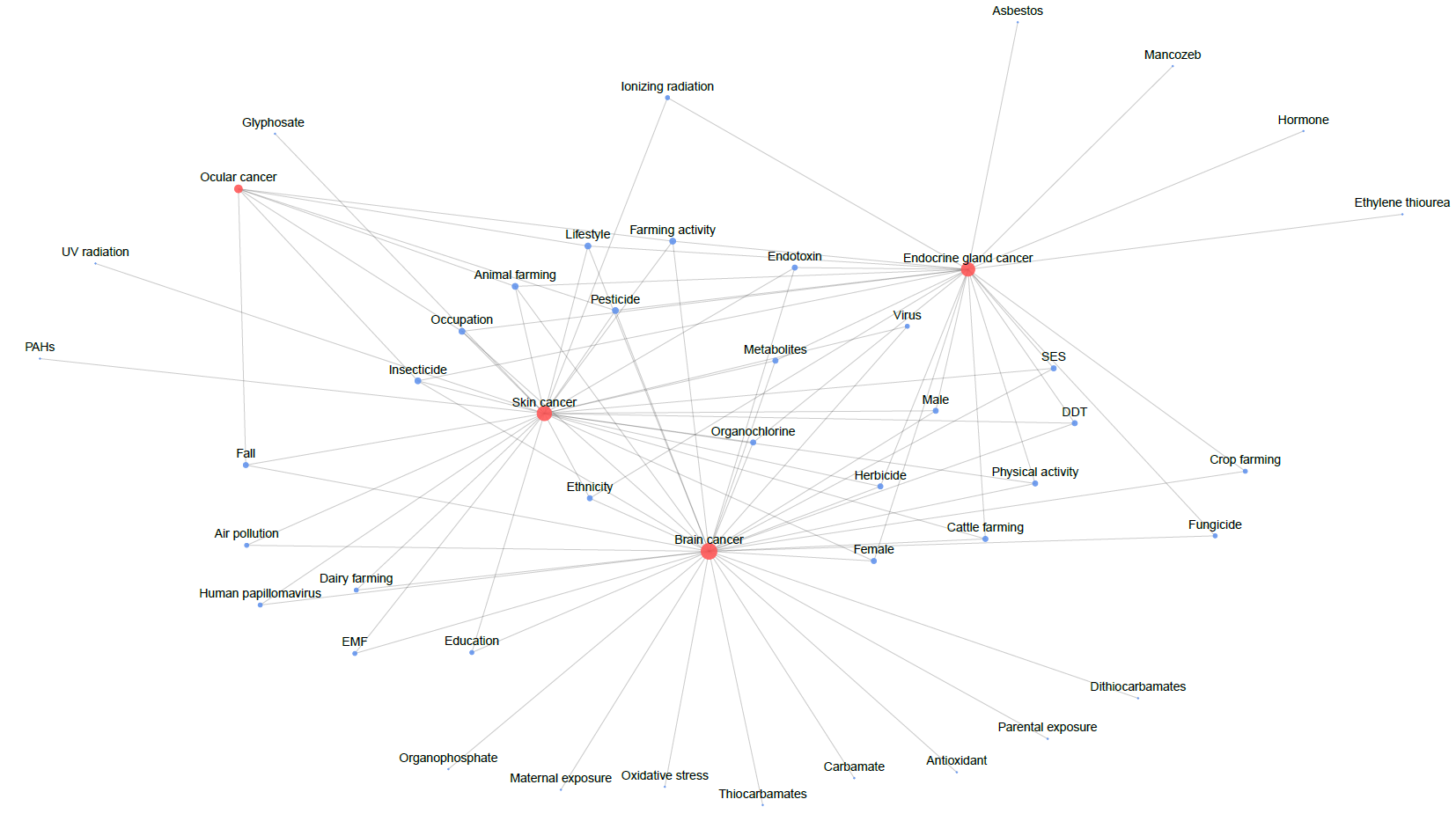


# **Figure S31**. Chord diagram of keyword co-occurrence between potential risk factor and respiratory and urinary tract cancer keywords.

DDT: dichlorodiphenyltrichloroethane, EMF: electromagnetic field, ETS: environmental tobacco smoking, PAH: polycyclic aromatic hydrocarbon, SES: socio-economic status.


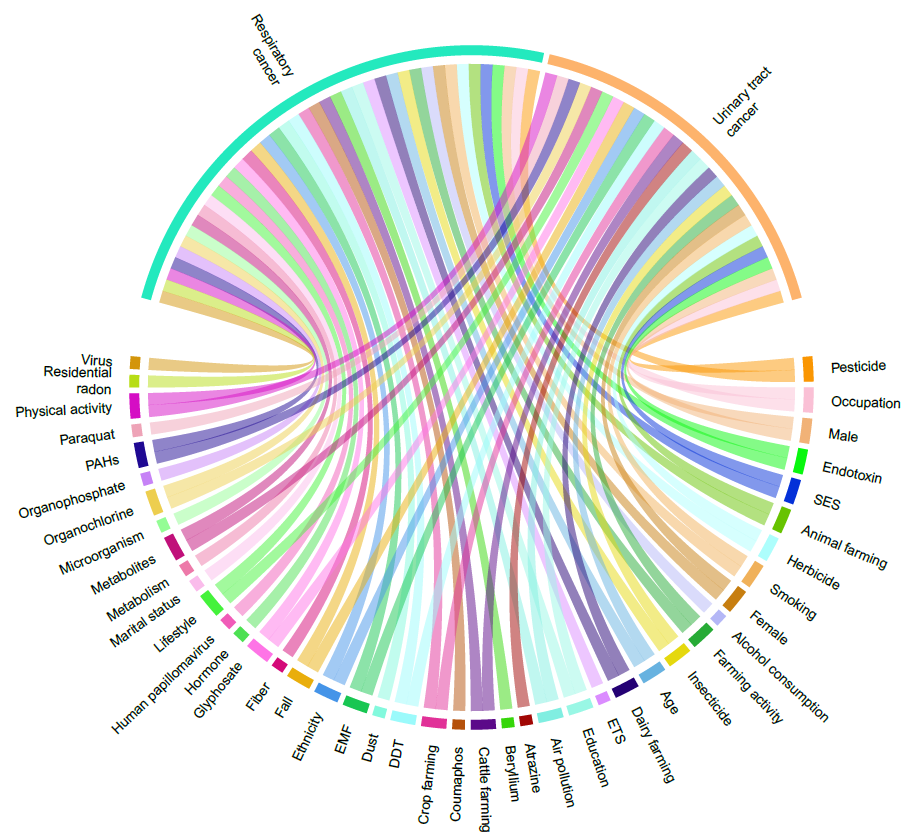


# **Figure S32**. Keyword co-occurrence network between potential risk factor and respiratory and urinary tract cancer keywords.

DDT: dichlorodiphenyltrichloroethane, EMF: electromagnetic field, ETS: environmental tobacco smoking, PAH: polycyclic aromatic hydrocarbon, SES: socio-economic status. Larger the node size, higher the number of co-occurrence. Red nodes refer to health events while blue nodes refer to potential risk factors.


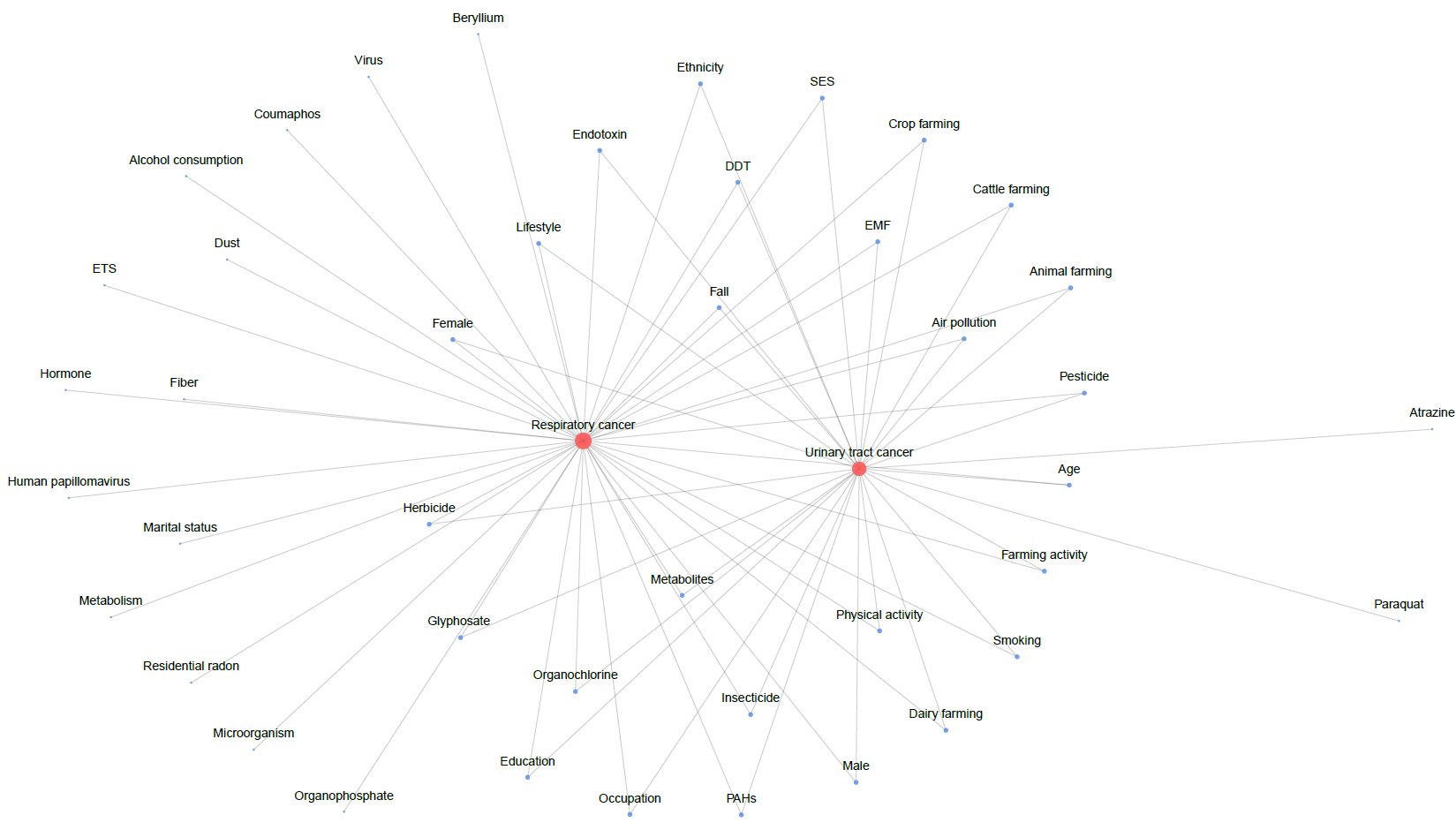


# **Figure S33**. Chord diagram of keyword co-occurrence between potential risk factor and infectious disease keywords.

SES: socio-economic status.


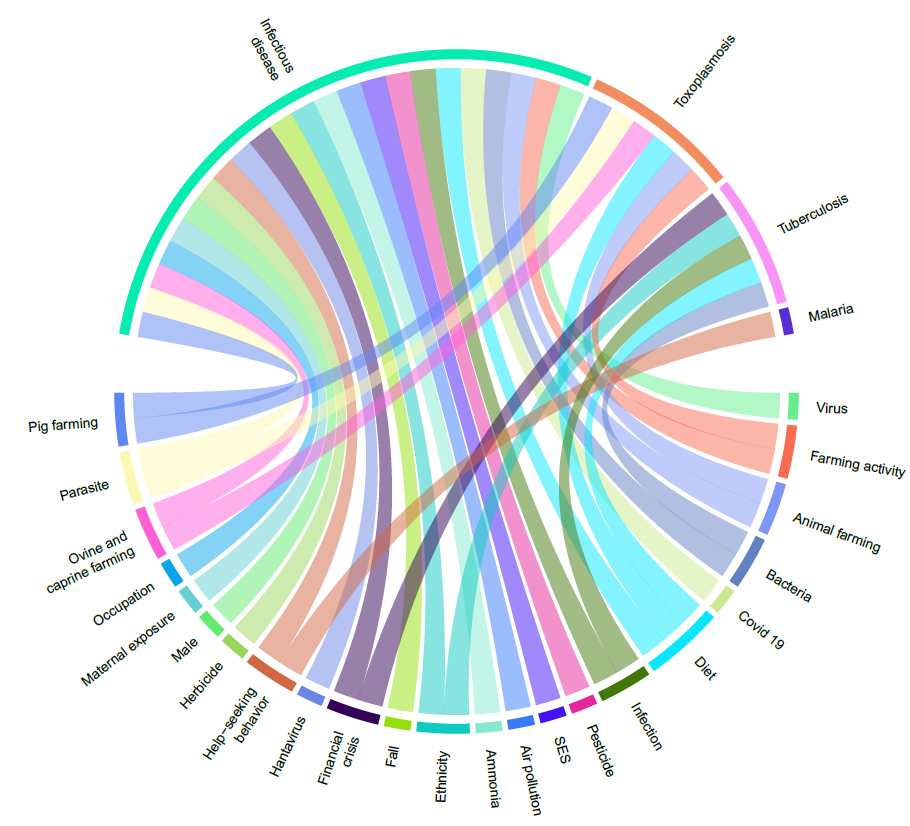


# **Figure S34**. Keyword co-occurrence network between potential risk factor and infectious disease keywords.

SES: socio-economic status. Larger the node size, higher the number of co-occurrence. Red nodes refer to health events while blue nodes refer to potential risk factors.


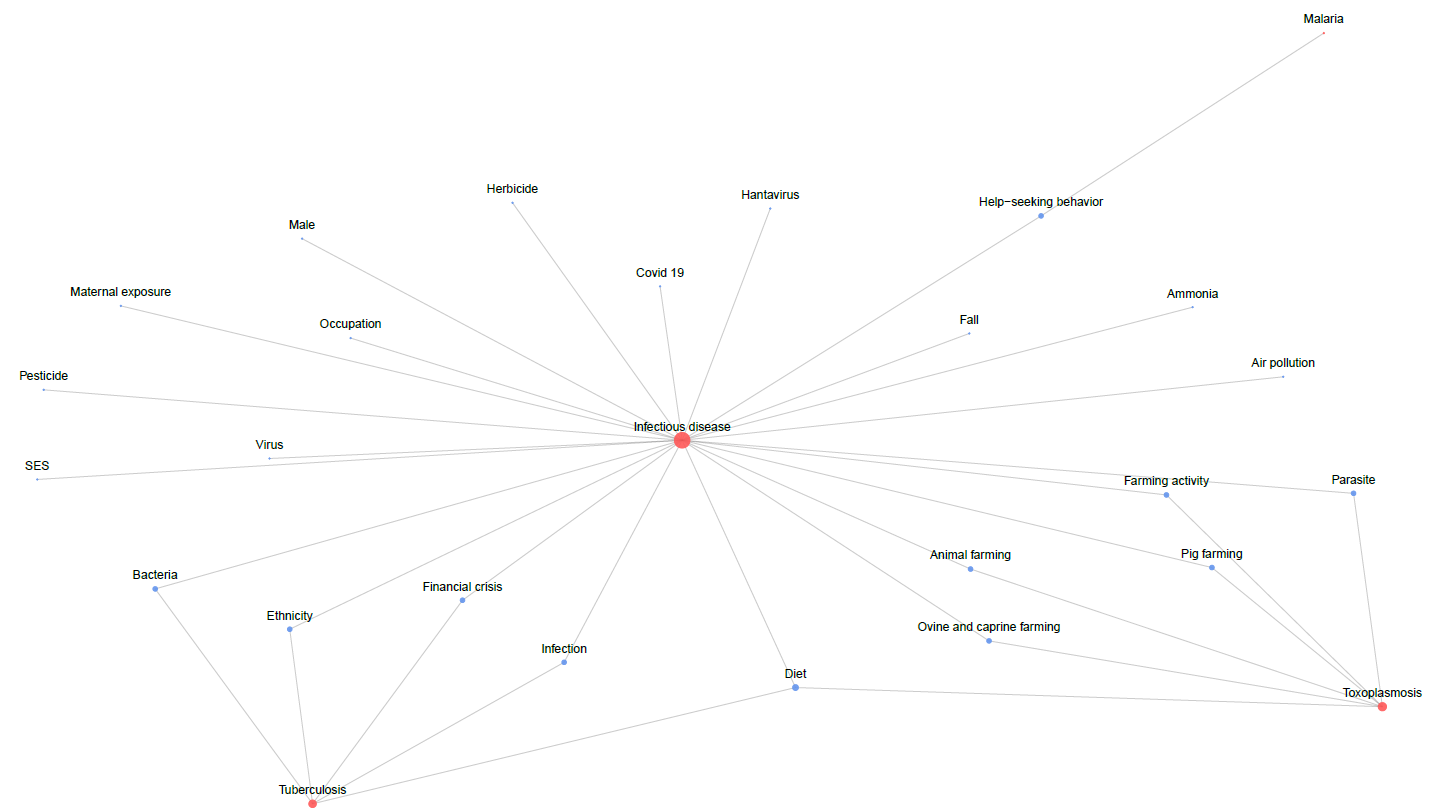


# **Figure S35**. Chord diagram of keyword co-occurrence between potential risk factor and respiratory disorder keywords.

BMI: body mass index, BP: blood pressure, COPD: chronic obstructive pulmonary disease, IgE: immunoglobulin E, IgG: immunoglobulin G, psy: psychosocial, SES: socio-economic status.


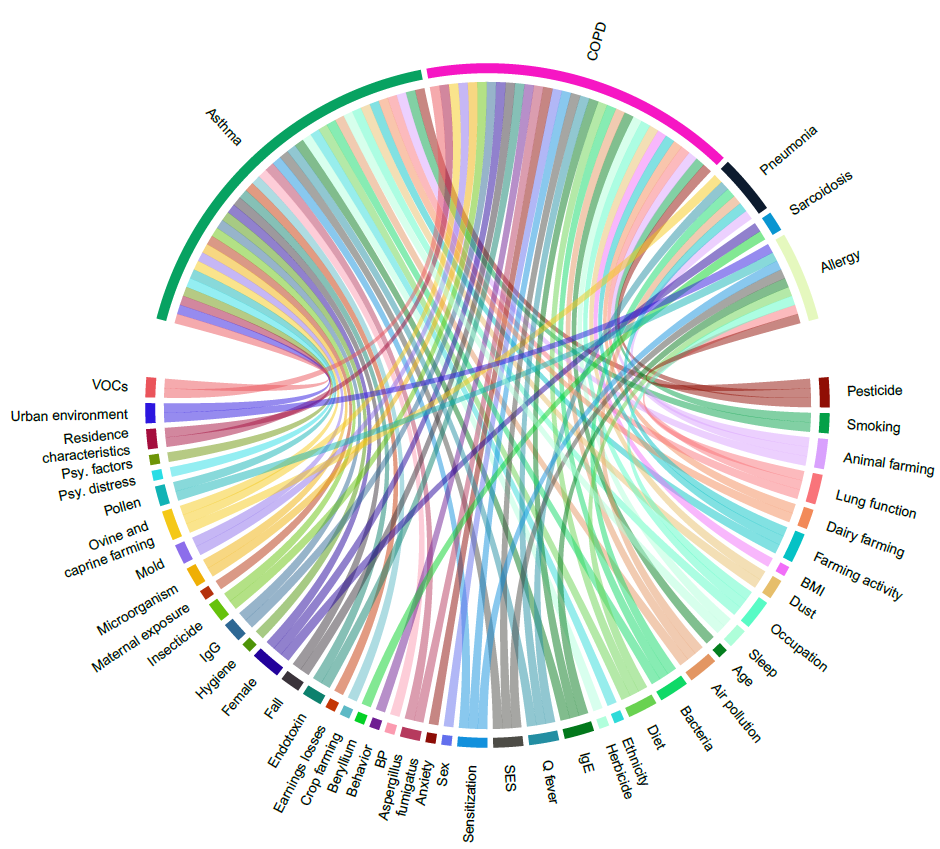


# **Figure S36**. Keyword co-occurrence network between potential risk factor and respiratory disorder keywords.

BMI: body mass index, BP: blood pressure, COPD: chronic obstructive pulmonary disease, IgE: immunoglobulin E, IgG: immunoglobulin G, psy: psychosocial, SES: socio-economic status. Larger the node size, higher the number of co-occurrence. Red nodes refer to health events while blue nodes refer to potential risk factors.


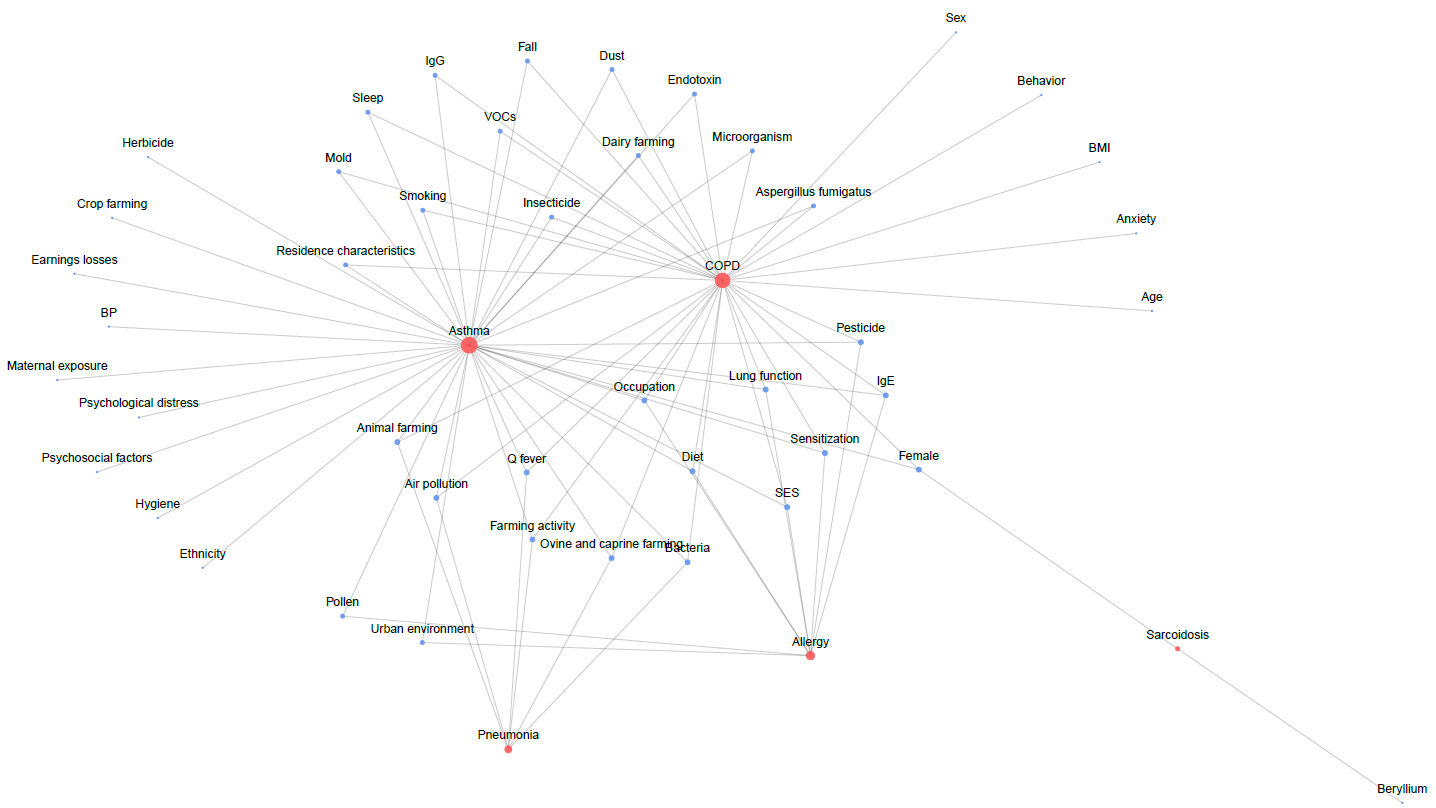


# **Figure S37**. Chord diagram of keyword co-occurrence between potential risk factor and reproductive disorder keywords.

BMI: body mass index, SES: socio-economic status.


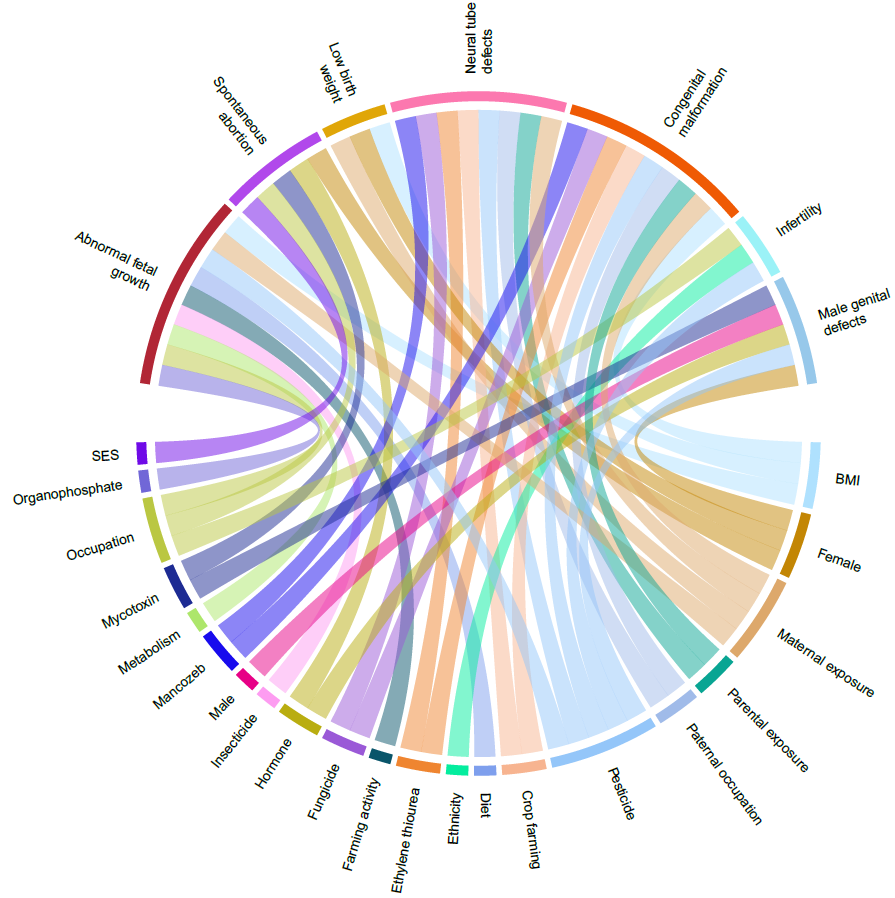


# **Figure S38**. Keyword co-occurrence network between potential risk factor and reproductive disorder keywords.

BMI: body mass index, SES: socio-economic status. Larger the node size, higher the number of co-occurrence. Red nodes refer to health events while blue nodes refer to potential risk factors.


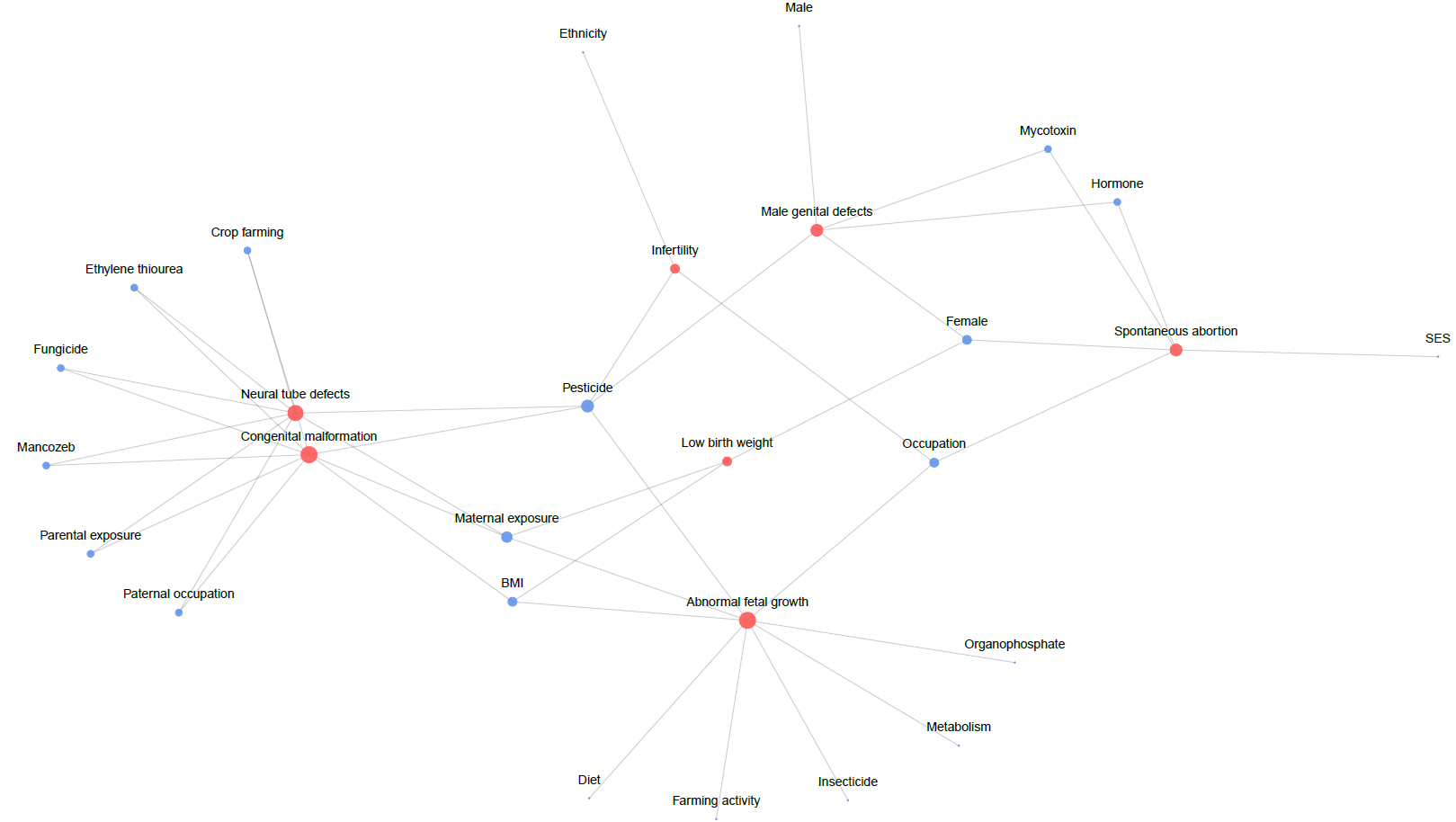


# **Figure S39**. Chord diagram of keyword co-occurrence between potential risk factor and injury and work-related disease keywords.

AAW: workplace accident, BMI: body mass index, MSD: musculoskeletal disorder, psy: psychological, SES: socio-economic status, WRD: work-related disease.


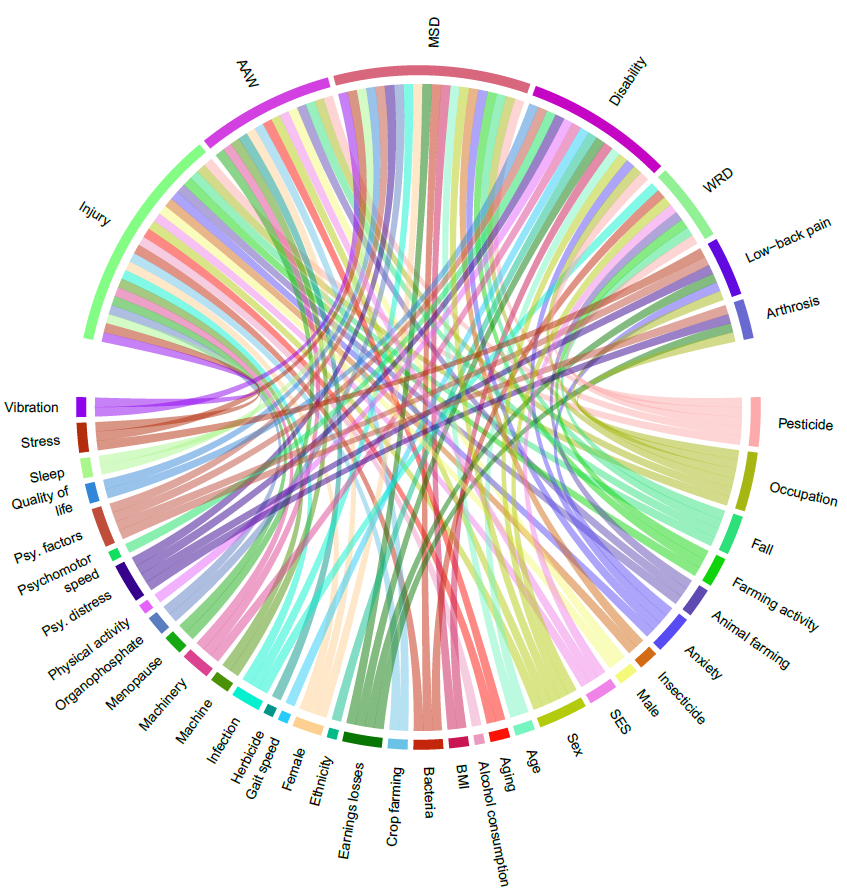


# **Figure S40**. Keyword co-occurrence network between potential risk factor and injury and work-related disease keywords.

AAW: workplace accident, BMI: body mass index, MSD: musculoskeletal disorder, psy: psychological, SES: socio-economic status, WRD: work-related disease. Larger the node size, higher the number of co-occurrence. Red nodes refer to health events while blue nodes refer to potential risk factors.


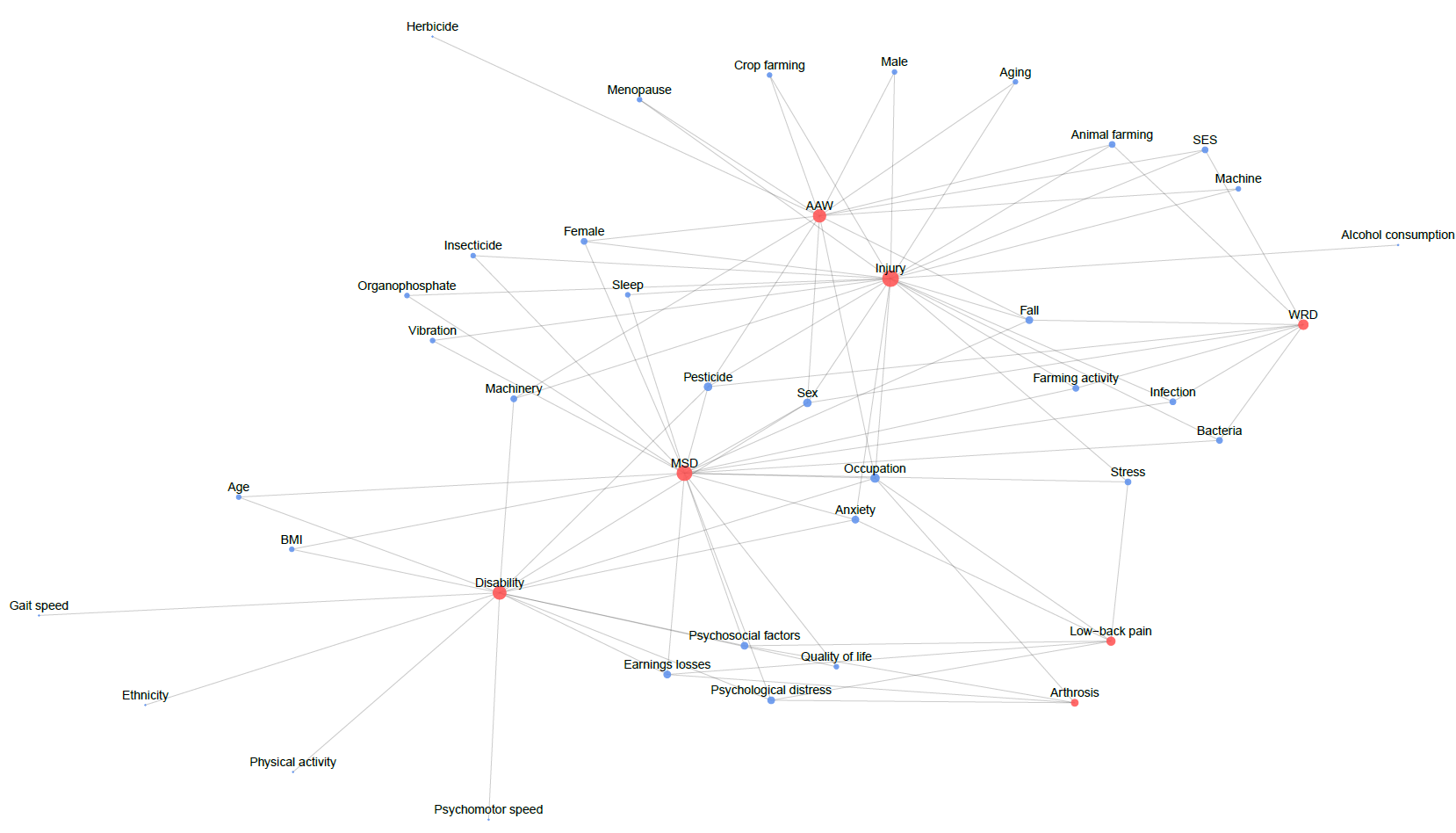


# **Figure S41**. Chord diagram of keyword co-occurrence between potential risk factor and other health event keywords.

BMI: body mass index, BP: blood pressure, CKD: chronic kidney disease, CVD: cardiovascular disease, psy: psychological, SES: socio-economic status.


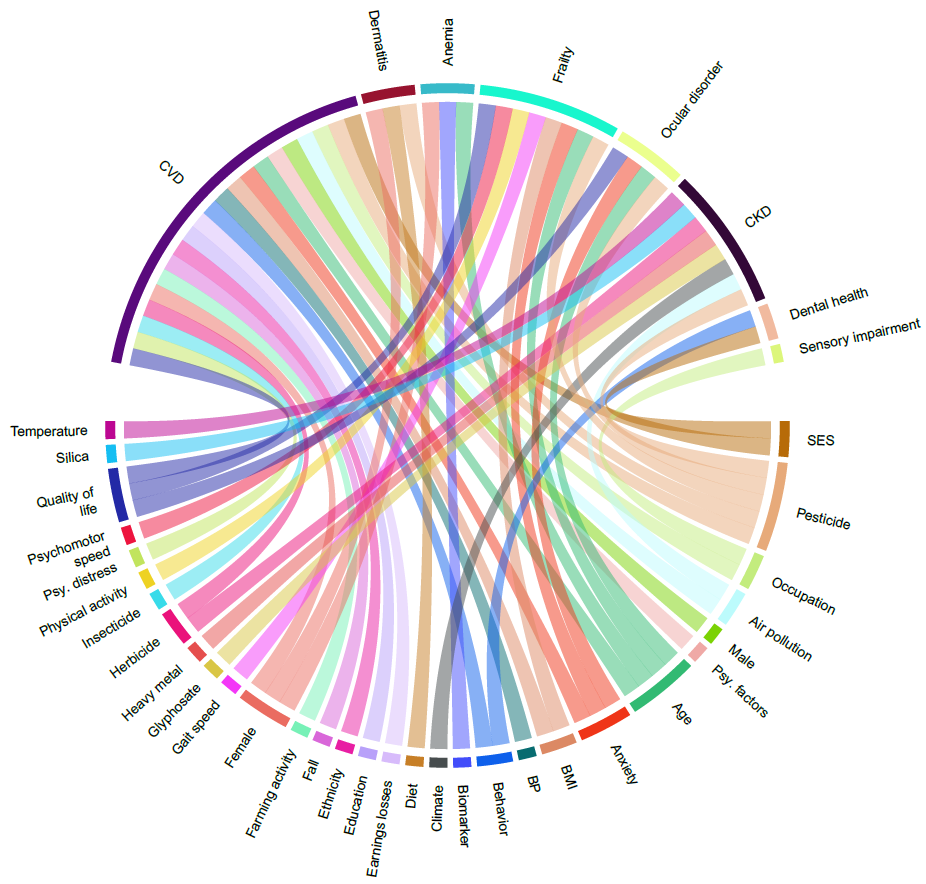


# **Figure S42**. Keyword co-occurrence network between potential risk factor and other health event keywords.

BMI: body mass index, BP: blood pressure, CKD: chronic kidney disease, CVD: cardiovascular disease, psy: psychological, SES: socio-economic status. Larger the node size, higher the number of co-occurrence. Red nodes refer to health events while blue nodes refer to potential risk factors.


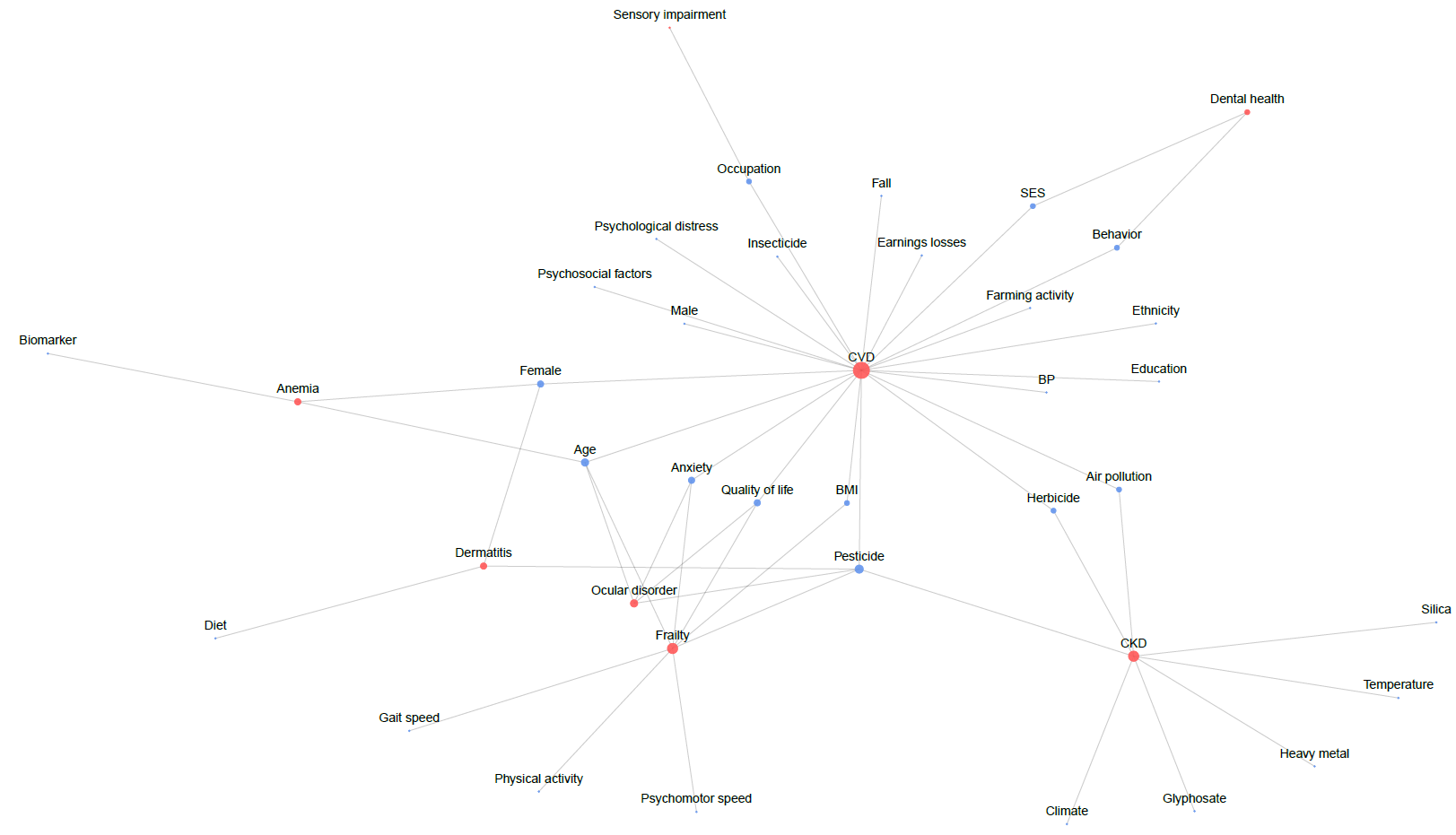


# **Figure S43**. Keyword co-occurrence network between potential risk factor and mortality keywords.

DBCP: 1,2-dibromo-3-chloropropane, EMF: electromagnetic field, ETS: environmental tobacco smoking, PAH: polycyclic aromatic hydrocarbon, SES: socio-economic status, VOC: volatile organic compound. Larger the node size, higher the number of co-occurrence. Red nodes refer to health events while blue nodes refer to potential risk factors.


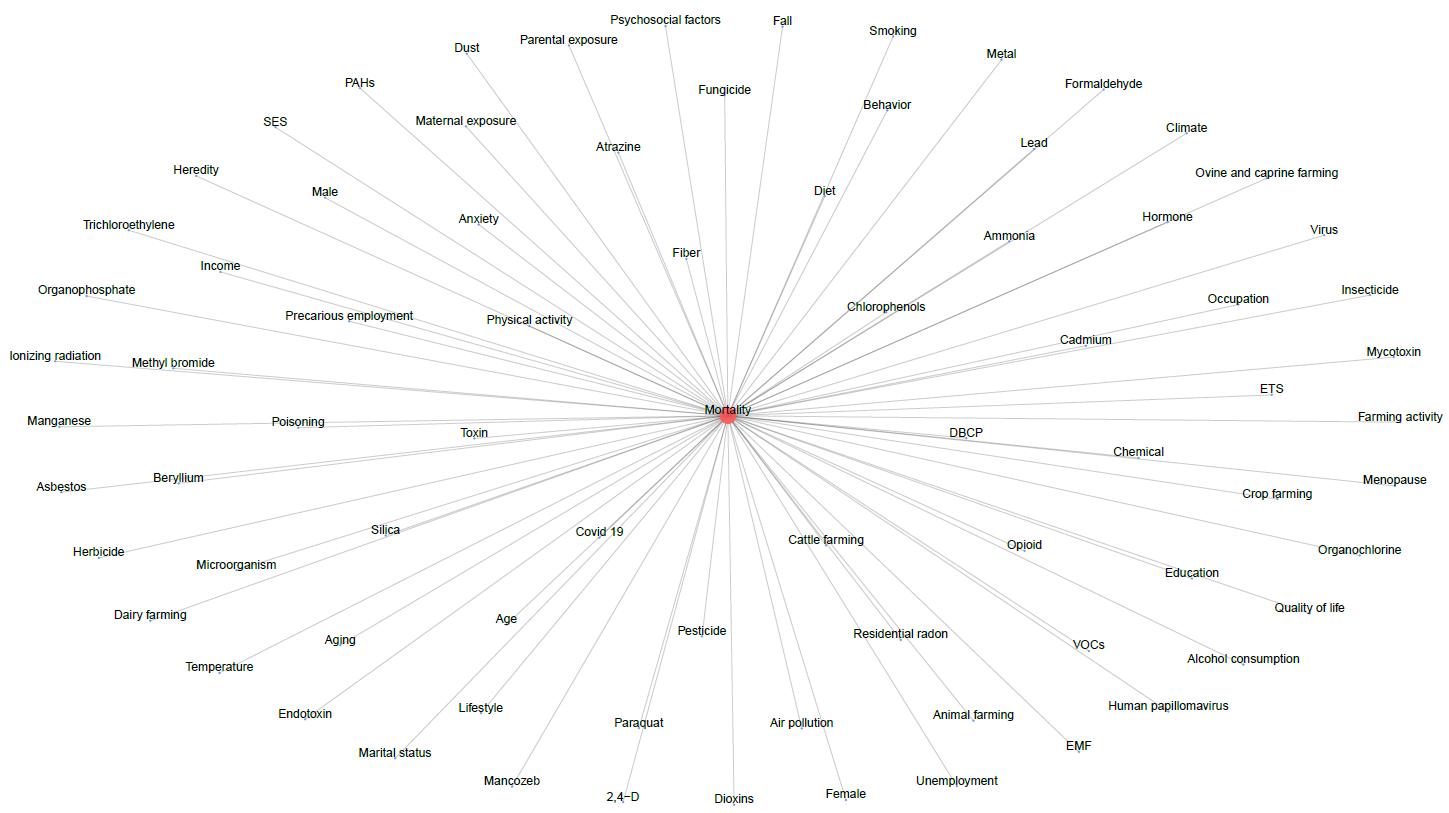


# **Abbreviations**

90%CI: 90% confidence interval

AAW: workplace accident

AGEIS: Autonomy, Gerontology, E-health, Imaging & Society (Autonomie, Gérontologie, E-santé, Imagerie et Société in French)

AGR: annual growth rate

AGRICAN: Agriculture and Cancer

AHD: administrative health database

AHS: Agricultural Health Study

AI: artificial intelligence

AMI: Aging Multidisciplinary Investigation

ANR: French National Research Agency (Agence Nationale de la Recherche in French)

AS: association strength

BIBLIO: preliminary guideline for reporting bibliometric reviews of the biomedical literature

BMI: body mass index

CHU: university hospital (centre hospitalier universitaire in French)

COPD: chronic obstructive pulmonary disease

CNAP: Cancer in the Norwegian Agricultural population cohort

CVD: cardiovascular disorder

DDT: dichlorodiphenyltrichloroethane

EHR: electronic health record

EMR: electronic medical record

FAIR: findable, accessible, interoperable and reusable

FERMA: risk factors of the rural environment and the allergic and respiratory disease

FF: fractional frequency

FINJEM: Finnish information system on occupational exposure

GDP: gross domestic product

IARC: International Agency for Research on Cancer

IBD: inflammatory bowel disease

ICMJE: International Committee of Medical Journal Editors

INCa: French national cancer institute (Institut National du Cancer in French)

INSERM: French National Institute of Health and Medical Research (Institut national de la santé et de la recherche médicale in French)

IQR: interquartile range

JEM: job-exposure matrix

mHealth: mobile health

MIAI: Multidisciplinary Institute in Artificial Intelligence

ML: machine learning

MSA: National Health Insurance Fund for Agricultural Workers and Farmers (Mutualité Sociale Agricole in French)

MSD: musculoskeletal disorder

NAWS: National Agricultural Workers Survey

NCI: National Cancer Institute

NIH: National Institutes of Health

NOCCA: Nordic Occupational Cancer Study

ORCID: Open Researcher and Contributor ID

PFAS: per- and polyfluoroalkyl substances

PRISMA-ScR: Preferred Reporting Items for Systematic Reviews and Meta-Analyses extension for Scoping Reviews and evidence maps

Q1: first quartile

R&D: research and development

SES: socio-economic status

TEDI: toxicological and exposure database inventory

TRACTOR: Tracking and monitoring occupational risks in agriculture

UFW: United Farm Workers of America

UGA: Université Grenoble Alpes

UK: United Kingdom

US: United States

UV: ultraviolet

# **References**

1. Bienert IR, Oliveira RC, Andrade PB, Caramori CA. Bibliometric indexes, databases and impact factors in cardiology. Rev Bras Cir Cardiovasc 2015;30(2):254–259. doi:[10.5935/1678-9741.20150019](https://doi.org/10.5935/1678-9741.20150019).
2. Bihari A, Tripathi S, Deepak A. A review on h-index and its alternative indices. J Inf Sci 2023;49(3):624–665 (2023). doi:[10.1177/0165551521101447](https://doi.org/10.1177/0165551521101447).
3. Shao Y, Chien TW, Jang FL. The use of radar plots with the Yk-index to identify which authors contributed the most to the journal of Medicine in 2020 and 2021: A bibliometric analysis. Medicine (Baltimore) 2022;101(45):e31033. doi:[10.1097%2FMD.0000000000031033](https://doi.org/10.1097%2FMD.0000000000031033).
4. Kumar S, Kumar S. Trends of collaborative research in journals of oilseeds research (India), 1993-2004. Indian J Agric Library Inf Services 2008;24:80–90.
5. Wu T, Duan Y, Zhang T, Tian W, Liu H, Deng Y. Research Trends in the Application of Artificial Intelligence in Oncology: A Bibliometric and Network Visualization Study. Front Biosci (Landmark Ed). 2022;27(9):254 doi:10.31083/j.fbl2709254.
6. Pukkala E, Martinsen JI, Lynge E, et al. Occupation and cancer - follow-up of 15 million people in five Nordic countries. Acta Oncol. 2009;48(5):646-790 doi:10.1080/02841860902913546.
7. Alavanja MC, Samanic C, Dosemeci M, et al. Use of agricultural pesticides and prostate cancer risk in the Agricultural Health Study cohort. Am J Epidemiol 2003;157(9):800-814. doi:10.1093/aje/kwg040.
8. Elbaz A, Clavel J, Rathouz PJ, et al. Professional exposure to pesticides and Parkinson disease. Ann Neurol 2009;66(4):494-504. doi:10.1002/ana.21717.
9. Andersen A, Barlow L, Engeland A, Kjaerheim K, Lynge E, Pukkala E. Work-related cancer in the Nordic countries. Scand J Work Environ Health 1999;25 Suppl 2:1-116. PMID: 10507118.
10. Karjalainen A, Kurppa K, Martikainen R, Klaukka T, Karjalainen J. Work is related to a substantial portion of adult-onset asthma incidence in the Finnish population. Am J Respir Crit Care Med 2001;164(4):565-568. doi:10.1164/ajrccm.164.4.2012146.
11. Vingård E, Alfredsson L, Goldie I, Hogstedt C. Occupation and osteoarthrosis of the hip and knee: a register-based cohort study. Int J Epidemiol 1991;20(4):1025-1031. doi:10.1093/ije/20.4.1025.
12. Alavanja MC, Dosemeci M, Samanic C, et al. Pesticides and lung cancer risk in the agricultural health study cohort. Am J Epidemiol 2004;160(9):876-885. doi:10.1093/aje/kwh290.
13. Andreotti G, Koutros S, Hofmann JN, et al. Glyphosate Use and Cancer Incidence in the Agricultural Health Study. J Natl Cancer Inst 2018;110(5):509-516. doi:10.1093/jnci/djx233.
14. Wigle DT, Semenciw RM, Wilkins K, et al. Mortality study of Canadian male farm operators: non-Hodgkin's lymphoma mortality and agricultural practices in Saskatchewan. J Natl Cancer Inst 1990;82(7):575-582. doi:10.1093/jnci/82.7.575.
15. Engel LS, Hill DA, Hoppin JA, et al. Pesticide use and breast cancer risk among farmers' wives in the agricultural health study. Am J Epidemiol 2005;161(2):121-135. doi:10.1093/aje/kwi022.
16. Hemminki K, Saloniemi I, Salonen T, Partanen T, Vainio H. Childhood cancer and parental occupation in Finland. J Epidemiol Community Health 1981;35(1):11-15. doi:10.1136/jech.35.1.11.
17. Weichenthal S, Villeneuve PJ, Burnett RT, et al. Long-term exposure to fine particulate matter: association with nonaccidental and cardiovascular mortality in the agricultural health study cohort. Environ Health Perspect 2014;122(6):609-615. doi:10.1289/ehp.1307277.
18. Levecque C, Elbaz A, Clavel J, et al. Association between Parkinson's disease and polymorphisms in the nNOS and iNOS genes in a community-based case-control study. Hum Mol Genet 2003;12(1):79-86. doi:10.1093/hmg/ddg009.
19. Morrison H, Savitz D, Semenciw R, et al. Farming and prostate cancer mortality. Am J Epidemiol 1993;137(3):270-280. doi:10.1093/oxfordjournals.aje.a116674.
20. Kristensen P, Andersen A, Irgens LM, Laake P, Bye AS. Incidence and risk factors of cancer among men and women in Norwegian agriculture. Scand J Work Environ Health 1996;22(1):14-26. doi:10.5271/sjweh.104.
21. Orton SM, Wald L, Confavreux C, et al. Association of UV radiation with multiple sclerosis prevalence and sex ratio in France. Neurology 2011;76(5):425-431. doi:10.1212/WNL.0b013e31820a0a9f.
22. Stokes L, Stark A, Marshall E, Narang A. Neurotoxicity among pesticide applicators exposed to organophosphates. Occup Environ Med 1995;52(10):648-653. doi:10.1136/oem.52.10.648.
23. Vukusic S, Van Bockstael V, Gosselin S, Confavreux C. Regional variations in the prevalence of multiple sclerosis in French farmers. J Neurol Neurosurg Psychiatry 2007;78(7):707-709. doi:10.1136/jnnp.2006.101196.
24. Mills PK, Yang R. Prostate cancer risk in California farm workers. J Occup Environ Med 2003;45(3):249-258. doi:10.1097/01.jom.0000058339.05741.0c.
25. Eriksson M, Karlsson M. Occupational and other environmental factors and multiple myeloma: a population based case-control study. Br J Ind Med 1992;49(2):95-103. doi:10.1136/oem.49.2.95.
26. Wiklund K, Dich J, Holm LE, Eklund G. Risk of cancer in pesticide applicators in Swedish agriculture. Br J Ind Med 1989;46(11):809-814. doi:10.1136/oem.46.11.809.
27. Pouchieu C, Piel C, Carles C, et al. Pesticide use in agriculture and Parkinson's disease in the AGRICAN cohort study. Int J Epidemiol 2018;47(1):299-310. doi:10.1093/ije/dyx225.
28. Karjalainen A, Martikainen R, Klaukka T, Saarinen K, Uitti J. Risk of asthma among Finnish patients with occupational rhinitis. Chest 2003;123(1):283-288. doi:10.1378/chest.123.1.283.
29. Leon ME, Schinasi LH, Lebailly P, et al. Pesticide use and risk of non-Hodgkin lymphoid malignancies in agricultural cohorts from France, Norway and the USA: a pooled analysis from the AGRICOH consortium. Int J Epidemiol 2019;48(5):1519-1535. doi:10.1093/ije/dyz017.
30. Lee WJ, Sandler DP, Blair A, Samanic C, Cross AJ, Alavanja MC. Pesticide use and colorectal cancer risk in the Agricultural Health Study. Int J Cancer 2007;121(2):339-346. doi:10.1002/ijc.22635.
31. Reif J, Pearce N, Fraser J. Cancer risks in New Zealand farmers. Int J Epidemiol 1989;18(4):768-774. doi:10.1093/ije/18.4.768.
